# Global burden associated with 85 pathogens in 2019: a systematic analysis for the Global Burden of Disease Study 2019

**DOI:** 10.1016/S1473-3099(24)00158-0

**Published:** 2024-08

**Authors:** IHME Pathogen Core Group, Mohsen Naghavi, Tomislav Mestrovic, Authia Gray, Anna Gershberg Hayoon, Lucien R Swetschinski, Gisela Robles Aguilar, Nicole Davis Weaver, Kevin S Ikuta, Erin Chung, Eve E Wool, Chieh Han, Daniel T Araki, Samuel B Albertson, Rose Bender, Greg Bertolacci, Annie J Browne, Ben S Cooper, Matthew W Cunningham, Christiane Dolecek, Matthew Doxey, Susanna J Dunachie, Sama Ghoba, Georgina Haines-Woodhouse, Simon I Hay, Rebecca L Hsu, Kenneth C Iregbu, Hmwe H Kyu, Jorge R Ledesma, Jianing Ma, Catrin E Moore, Jonathan F Mosser, Vincent Mougin, Pirouz Naghavi, Amanda Novotney, Victor Daniel Rosenthal, Benn Sartorius, Andy Stergachis, Christopher Troeger, Avina Vongpradith, Magdalene K Walters, Han Yong Wunrow, Christopher JL Murray

## Abstract

**Background:**

Despite a global epidemiological transition towards increased burden of non-communicable diseases, communicable diseases continue to cause substantial morbidity and mortality worldwide. Understanding the burden of a wide range of infectious diseases, and its variation by geography and age, is pivotal to research priority setting and resource mobilisation globally.

**Methods:**

We estimated disability-adjusted life-years (DALYs) associated with 85 pathogens in 2019, globally, regionally, and for 204 countries and territories. The term pathogen included causative agents, pathogen groups, infectious conditions, and aggregate categories. We applied a novel methodological approach to account for underlying, immediate, and intermediate causes of death, which counted every death for which a pathogen had a role in the pathway to death. We refer to this measure as the burden associated with infection, which was estimated by combining different sources of information. To compare the burden among all pathogens, we used pathogen-specific ratios to incorporate the burden of immediate and intermediate causes of death for pathogens modelled previously by the GBD. We created the ratios by using multiple cause of death data, hospital discharge data, linkage data, and minimally invasive tissue sampling data to estimate the fraction of deaths coming from the pathway to death chain. We multiplied the pathogen-specific ratios by age-specific years of life lost (YLLs), calculated with GBD 2019 methods, and then added the adjusted YLLs to age-specific years lived with disability (YLDs) from GBD 2019 to produce adjusted DALYs to account for deaths in the chain. We used standard GBD methods to calculate 95% uncertainty intervals (UIs) for final estimates of DALYs by taking the 2·5th and 97·5th percentiles across 1000 posterior draws for each quantity of interest. We provided burden estimates pertaining to all ages and specifically to the under 5 years age group.

**Findings:**

Globally in 2019, an estimated 704 million (95% UI 610–820) DALYs were associated with 85 different pathogens, including 309 million (250–377; 43·9% of the burden) in children younger than 5 years. This burden accounted for 27·7% (and 65·5% in those younger than 5 years) of the previously reported total DALYs from all causes in 2019. Comparing super-regions, considerable differences were observed in the estimated pathogen-associated burdens in relation to DALYs from all causes, with the highest burden observed in sub-Saharan Africa (314 million [270–368] DALYs; 61·5% of total regional burden) and the lowest in the high-income super-region (31·8 million [25·4–40·1] DALYs; 9·8%). Three leading pathogens were responsible for more than 50 million DALYs each in 2019: tuberculosis (65·1 million [59·0–71·2]), malaria (53·6 million [27·0–91·3]), and HIV or AIDS (52·1 million [46·6–60·9]). Malaria was the leading pathogen for DALYs in children younger than 5 years (37·2 million [17·8–64·2]). We also observed substantial burden associated with previously less recognised pathogens, including *Staphylococcus aureus* and specific Gram-negative bacterial species (ie, *Klebsiella pneumoniae, Escherichia coli, Pseudomonas aeruginosa, Acinetobacter baumannii*, and *Helicobacter pylori*). Conversely, some pathogens had a burden that was smaller than anticipated.

**Interpretation:**

Our detailed breakdown of DALYs associated with a comprehensive list of pathogens on a global, regional, and country level has revealed the magnitude of the problem and helps to indicate where research funding mismatch might exist. Given the disproportionate impact of infection on low-income and middle-income countries, an essential next step is for countries and relevant stakeholders to address these gaps by making targeted investments.

**Funding:**

Bill & Melinda Gates Foundation, Wellcome Trust, and Department of Health and Social Care using UK aid funding managed by the Fleming Fund.

## Introduction

The Global Burden of Diseases, Injuries, and Risk Factors Study (GBD) documents the impact of communicable diseases as underlying causes of death throughout each cycle of the study.^1^, ^2^ Although this information is important to understand the relative magnitude of burden of these causes, it might not capture the full impact of pathogens, which is potentially useful to consider for drug and vaccine development efforts. Notably, the International Classification of Diseases (ICD) highlights that pathogens have a role in the pathway to death for many individuals with non-communicable diseases or injuries. More specifically, the chain of death consists of underlying causes (which initiate the chain) and several intermediate causes. By effectively understanding and managing these intermediate causes, the risk of death can be substantially reduced, particularly in cases in which non-communicable diseases are the underlying cause. For instance, individuals with diabetes mellitus and kidney failure that necessitates dialysis might die from *Staphylococcus aureus* sepsis.^3^, ^4^ Quantifying a comprehensive disease burden associated with pathogens can provide a valuable supplement to the analyses of underlying causes of death.

**Research in context**

**Evidence before this study**

We previously reported the mortality associated with 33 bacterial pathogens in 2019 and showed that five relatively infrequently highlighted pathogens (S*taphylococcus aureus, Escherichia coli, Streptococcus pneumoniae, Klebsiella pneumoniae,* and *Pseudomonas aeruginosa*) were responsible for more than half of all bacterial-caused deaths. We did not previously incorporate the other important pathogen groups of viruses, fungi, and parasites. Furthermore, we did not calculate the full burden of disease expressed in disability-adjusted life-years (DALYs), or the sum of the years of life lost (YLL) due to premature mortality and years lived with disability (YLD) due to the occurrence of disease. A literature search between Nov 1, 2019 and Dec 31, 2023, in PubMed, Medline, Web of Science, and Scopus (with key words “burden”, “pathogens”, “infectious diseases”, “morbidity”, “disability-adjusted life-years”, and “mortality”), showed that the existing literature is infrequent, and no studies have pursued such estimations in all pathogen groups. This limited audit, in terms of pathogen range, and the omission of associated non-fatal burden in our previous work and other studies complicates global strategising around the development of antimicrobial agents and vaccines. Additional details of our literature review are available in the appendix (pp 4–6).

**Added value of this study**

This is the first study, to our knowledge, to document the DALY burden of 85 pathogens globally, representing all communicable disease groups, including bacteria, viruses, fungi, and parasites. With respect to death certificate terminology, this study presents the burden of infectious diseases as underlying, immediate, and intermediate causes of death, constituting what we call the pathway to death burden, producing the most inclusive measure of infectious disease burden to date. This approach is in contrast with some previous results, which have documented the burden of infections as solely the underlying cause of death. Our more comprehensive assessment of infectious disease provides valuable context for many opportunistic pathogens that afflict those with comorbidities that would take precedent as the underlying cause; the burden of pathogens in such cases has not been previously calculated, and addressing these infections could reduce disability and prolong life. These burden estimates were calculated for all ages and for children younger than 5 years, at the global, regional, and national levels. We provide a geographically detailed assessment of the most burdensome pathogens in 2019, using DALYs to enable comprehensive and comparable assessments of their impact on both mortality and morbidity. Such an extensive analysis in the year before the onset of the COVID-19 pandemic will facilitate future comparisons on the topic.

**Implications of all the available evidence**

We estimated that 85 pathogens were associated with more than 700 million DALYs in 2019. Around three-quarters (n=63) of these infectious causes have no vaccine available, and the associated burden of these infectious causes without vaccines was disproportionately encountered by children younger than 5 years and people in resource-poor settings. The global dominance of tuberculosis, HIV or AIDS, and malaria in communicable disease burden remains. Our study also shows the under-recognised impact of less recognised pathogens (such as specific Gram-negative bacterial species, *S aureus,* and *Helicobacter pylori*) and indicates substantial between-country variation in the rankings of the most burdensome pathogens. Consequently, we show that many pathogens, and the diseases they cause, do not receive attention that is proportionate to their burden and the collective global need. This mismatch might be cause for reflection on the targets for development of new vaccines, therapeutics, and other public health interventions, especially for low-income settings and the under-5 age group.

Previous studies have attempted to quantify the burden associated with specific pathogens. Among these, our recent global analysis calculated deaths associated with 33 clinically significant bacterial pathogens (ie, regardless of underlying cause of death).^5^ The findings highlighted the years of life lost associated with bacterial infections but did not produce any non-fatal burden estimates. Existing work might therefore underestimate the full burden of these infections while over-representing the burden of bacteria that lead to fatal infection. An essential aspect is to consider outcomes beyond mortality when assessing the full magnitude of impact of communicable diseases. Thus, a comprehensive global estimate, with a consistent approach to estimating associated burden across all pathogens such as viruses, fungi, and parasites, is needed to inform public health policy, and guide drug and vaccine development priorities. For example, a new highly efficacious vaccine for *Klebsiella pneumoniae* could prevent deaths when sepsis is the direct cause, but also reduce the probability of death for many patients who might be at increased risk of such infection (through dialysis or other causes). These indirect effects might substantially alter the calculus for stakeholders considering the cost versus benefit of potential new interventions against a finite research and development budget.

In this study, we provide a comprehensive global analysis of disability-adjusted life-years (DALYs) associated with 85 pathogens in 2019. DALYs consider the impact of years lived with disability (YLDs) following disease onset, and years of life lost (YLLs) due to premature mortality in comparison with a standardised life expectancy.^6^ This inclusive estimation of burden should help to inform effective control measures, optimise patient care, and contribute to the overall management of communicable diseases. Herein, we estimate the distribution of DALYs globally, by GBD super-region, and by country, with a focus on all age groups combined, and children younger than 5 years. We subsequently discuss findings with respect to prioritising therapeutics. This Article was produced as part of the GBD Collaborator Network and in accordance with the GBD Protocol.^7^

## Methods

### Overview and sources

In this study, we estimate DALYs associated with communicable diseases caused by 85 pathogens, spanning bacteria, viruses, fungi, and parasites across 204 countries and territories. We also consider not only single pathogens, but also pathogen groups and specific infectious conditions. More specifically, some pathogens were grouped due to small sample sizes and data availability, and we also wanted to address well established infectious conditions caused by a single or multiple microbial agents. The approach in this study was to combine three sources of information to estimate associated burden, which represents a burden for which a pathogen is on the pathway to death (as explained in the estimation process section). These sources were (1) capstone GBD 2019 estimates of underlying burden for 52 pathogens;^1^ (2) the ratio of associated burden to underlying burden according to multiple cause of death data, hospital discharge data, linkage data, and minimally invasive tissue sampling (MITS) data (datasets and data sources reported previously^1^); and (3) the associated burden of 33 bacterial pathogens previously studied.^5^ Our approach did not have any overlap between the pathogens obtained from sources (1) and (3). In estimating the burden associated with specific pathogens, we also included several aggregate categories: (1) fungi, (2) polymicrobial infections, (3) other neglected tropical diseases, and (4) other unspecified infectious diseases. For simplicity and readability, we use the term pathogens throughout the manuscript as a collective reference for all causative agents, pathogen groups, infectious conditions, and aggregate categories. This study included data on more than 21 million isolates and 140 790 sources for pathogens previously studied in the two GBD publications^1^, ^5^ (the data from Ikuta et al^5^ is presented in the Institute for Health Metrics and Evaluation (IHME) MICROBE tool). Full source counts by pathogen are available in the appendix (pp 36–38). Multiple cause of death data refers to information collected on death certificates that lists all causes, both underlying and associated (intermediate), contributing to an individual's death. Hospital discharge data provides information about the causes for which individuals are admitted to hospitals. Linkage data link individual-based hospital data to individual-based multiple cause of death data and offer a wider dataset that includes main diagnosis, other diagnoses, underlying cause of death, and intermediate causes of death in the chain. MITS is also known as a pathology-based autopsy which improves the understanding of mortality surveillance for children younger than 5 years, especially in low-income and middle-income settings. The appendix (pp 3–7) provides detailed information on the data sources.

We have primarily provided burden estimates pertaining to all ages and the younger than 5 years age group; however, we also showcase estimates specifically for the 5 years and older age group. The presentation in these formats aimed to offer a comprehensive analysis of the data across different age categories. Specifically, burden estimates for all age groups encompass the entire population under study, whereas estimates for the younger than 5 years group focus on the early childhood demographic.

This work complies with the Guidelines for Accurate and Transparent Health Estimates Reporting (GATHER) recommendations.^8^ The completed GATHER checklist is presented in the appendix (pp 55–56).

### Estimation process

Age-specific deaths were obtained for all ages, children younger than 5 years, and individuals aged 5 years and older, as detailed in the appendix (pp 25–30).

To convert the obtained age-specific deaths into age-specific YLLs, we used previously published GBD methods^1^ and referred to the standard counterfactual life expectancy at each age. The age-conditional life expectancy defined by the GBD 2019 reference life table assigns the same values to both male and female sexes. We calculated age-specific DALYs by adding the age-specific YLLs and age-specific YLDs (from GBD 2019)^1^ to present the burden associated with each pathogen, as explained herein.

To expand our analysis, we first needed to define the differences between three views on pathogen burden: (1) the pathway to death view, (2) the underlying cause view, and (3) the attributable cause view. The pathway to death analysis counts every death for which a pathogen had a role on the pathway to death. In ICD terms, these would include deaths for which the pathogen is listed on Part 1 of the WHO death certificate (immediate or intermediate causes), and deaths for which the pathogen was a direct cause. We refer to this burden as the “associated with” burden, which is the most inclusive measure and the one used in this manuscript. The underlying cause view counts every death for which the pathogen was the initiating event leading to death. In ICD terms, these would be deaths for which the pathogen is also listed on Part 1 of the WHO death certificate, but only as an underlying cause. This is the canonical ICD view encapsulated in the capstone GBD papers. Finally, the attributable cause view compares the deaths (and other morbid events) that occurred minus the deaths (and the events) that would have occurred in the absence of the pathogen. This counterfactual view would be the direct burden amenable to a specific intervention and thus of major interest to researchers hoping to develop the most impactful vaccine and therapeutic compounds.

The global burden of antimicrobial resistance^9^ and the burden of bacterial pathogens^5^ studies both used a pathway to death framework, in which events were included in the analysis if a pathogen was on the pathway to death (eg, a patient with diabetes in the intensive care unit who died from *E coli* sepsis would be included). Conversely, capstone GBD analyses by pathogen have been based on the ICD construct of underlying cause of death, focusing on the event initiating the series of events leading to death. For example, in the case of a patient with diabetes who died after developing Gram-negative sepsis, the GBD analysis would assign this death to diabetes. This approach ensures that deaths are not assigned to multiple causes.^1^

To adjust DALYs for the 52 pathogens in the GBD^1^ to account for the pathway to death, we used all available multiple cause of death data, hospital data, and MITS data with deaths as the outcome. To determine the ratio of associated burden to underlying burden, we divided the total number of deaths for which the pathogen occurred anywhere in the cause of death chain (as an underlying, intermediate, or immediate cause of death) by the total number of deaths for which the pathogen was the underlying cause of death to generate a ratio. A ratio of 1 indicated that no pathogen burden was lost by considering only the underlying cause of death estimates provided by the GBD 2019. We multiplied this ratio by the age-specific YLLs, and then added the adjusted YLLs to age-specific YLDs to produce adjusted age-specific DALYs, accounting for the complete pathway of death. We then used these adjusted DALYs to rank pathogens (globally and at the country level), and used the adjusted DALYs to estimate the fraction of the total number of DALYs from all causes (reported previously^1^) due to DALYs for the estimated pathogens (globally and for the seven GBD super-regions). More details on our approach are provided in the appendix (pp 7–30).

We calculated age-standardised DALY rates using GBD methods.^1^ First, age-specific DALY rates were calculated by dividing the number of DALYs for a specific age group by the population size of that age group (per previous demographic data^10^) and then multiplying by a constant factor to express the result per 100 000 population. Age-standardisation was done to adjust for differences in the age distribution of populations being compared, which allowed for increased accuracy in the comparison of disease burden between populations with different age structures. Consequently, age-standardised DALY rates were calculated by applying age-specific DALY rates from the study population to the GBD standard population with a specified age distribution,^10^ and are expressed per 100 000 population to facilitate comparison between populations. We used a direct method by multiplying age-specific rates in the study population by the corresponding age-specific proportions in the standard population, summing the products, and then dividing by the total standard population size. We also ranked pathogens by DALY rates, which allowed us to identify which pathogens contributed most substantially to disease burden. In this ranking, our focus was directed towards countries, specifically analysing the relative burden of pathogens, to gain insights into the differential impact of analysed pathogens, and to identify patterns and disparities.

We also estimated death rates for all ages and children younger than 5 years according to GBD super-region, as described in the appendix (p 35).

### Modelling

The data preparation involved mapping underlying causes of death or main diagnoses to causes listed in the GBD cause list, which is a mutually exclusive and collectively exhaustive list of diseases and injuries. This hierarchy categorises causes into broader groups at level 1 (eg, communicable diseases, non-communicable diseases, or injuries) and further refines them at level 2 into 22 cause groupings. More detailed disaggregation is provided at levels 3 and 4 for the finest level of detail. Subsequently, an infectious syndrome mapping hierarchy, termed the “AMR, sepsis, and infectious syndrome map”, was developed to link underlying and intermediate causes of death to infectious syndromes, facilitating nuanced analysis. The hierarchy included four levels, ensuring internal consistency across various metrics, and each level was mutually exclusive and collectively exhaustive, enabling effective aggregation and analysis. The process involved mapping underlying and intermediate causes of death and hospital diagnoses to specific infectious syndromes, such as bacterial infections of the skin and bloodstream infections, within this hierarchy. Furthermore, to address potential overlap in syndrome assignments due to multiple diagnoses associated with each record, an informative ranking hierarchy was implemented to prioritise the most informative infectious syndrome assignment on the basis of distinct pathogen distributions. Finally, two separate modelling pathways were used to estimate sepsis-related mortality fractions and infectious syndromes' contributions to sepsis-related mortality, ensuring accuracy and comprehensiveness in estimating pathogen burdens within the GBD framework. Further details on this approach are provided in the appendix (pp 8–11).

A brief overview of the modelling tools are provided herein; detailed descriptions have been published previously.^1^, ^5^ Premodelling bias adjustments were made with use of the Meta-Regression–Bayesian Regularised Trimmed tool (known as MR-BRT), a meta-regression tool that allows for Bayesian priors, regularisation, and trimming.^11^ Using these bias-adjusted data, we calculated an estimate of incidence or prevalence for every cause using the DisMod-MR 2.1 modelling framework.^1^ Spatiotemporal gaussian process regression was used to borrow strength between locations and over time for individual metrics of interest, and cause of death ensemble modelling was used to estimate the cause fraction for each underlying cause of death by age, sex, year, and location. In the pathogen distribution analysis, we implemented specific network meta-analyses using a previously established multinomial estimation with partial and composite observations modelling tool (known as MEPCO).^5^ This approach yielded pathogen-specific cause proportions (estimated by minimising the sum of the residuals between log-transformed observations and our predictions with use of the Gauss–Newton method). For pathogens associated with malignancy, we used specific diseases as a proxy for burden estimation. For example, to estimate the burden of human papillomavirus (HPV) we used cervical cancer as a proxy, as HPV is estimated to cause more than 99% of all cervical cancers (and there is no evidence of genetic predisposition).^12^ For *Helicobacter pylori*, on the basis of estimates from the International Agency for Research on Cancer, we removed cardia stomach cancer from the stomach cancer envelope, and attributed most of the remaining non-cardia cancer (mean attributable fraction 89%) to *H pylori*, preserving quantified uncertainty.^12^, ^13^, ^14^, ^15^

We used standard GBD methods^1^ to propagate the uncertainty from each analytical step into the final number of DALYs associated with each pathogen by generating 1000 draws from the posterior distribution of each quantity of interest and calculating the 2·5th and 97·5th percentiles.

### Role of the funding source

Co-authors affiliated with the funding organisations provided feedback on the initial maps and drafts of this manuscript. Otherwise, the funders of the study had no role in study design, data collection, data analysis, data interpretation, or the writing of the report.

## Results

**Figure 1:**
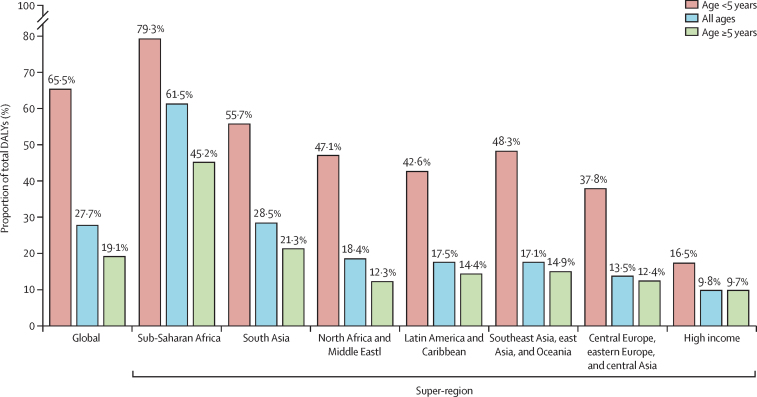
Proportion of total global and regional DALYs associated with 85 pathogens in 2019 Pathogen-associated DALY counts by super-region and all-cause DALY burden by super-region in 2019,^1^ for all ages and for the <5 years group, are provided in the appendix (pp 39–53). DALYs=disability-adjusted life-years.

Globally in 2019, an estimated 704 million (95% uncertainty interval [UI] 610–820) DALYs were associated with 85 pathogens, with 309 million (250–377; 43·9% of the burden) occurring in children younger than 5 years (table 1). The pathogen-associated burden in all age groups comprised 27·7% of the total number of DALYs from all causes in 2019 (2·54 billion DALYs;^1^ percentages are presented for unrounded values). In children younger than 5 years, the fraction of DALYs associated with these infectious causes was substantially greater at 65·5% of the overall DALY burden in 2019 (471 million;^1^ figure 1). Of the total 704 million DALYs, bacterial infections were associated with 415 million (58·9%), viral infections with 178 million (25·3%), parasitic infections with 72 million (10·2%), and fungal infections with 18·5 million (2·6%) in 2019, when summed for the individual pathogens (table 1).

**Table 1:** Distribution of DALYs associated with specific pathogens globally for all ages and children younger than 5 years, 2019

	**DALYs, count**		**DALYs, age-standardised rate per 100 000 population**	
	All ages	Age <5 years	All ages	Age <5 years
**Pathogens**				
*Acinetobacter baumannii*	16 700 000 (11 000 000–24 300 000)	6 580 000 (4 200 000–9 880 000)	215·8 (141·0–313·5)	992·7 (626·2–1489·9)
Adenovirus	5 960 000 (3 300 000–9 930 000)	5 050 000 (2 600 000–8 910 000)	77·0 (42·8–128·3)	761·8 (390·8–1344·1)
*Aeromonas* spp	1 590 000 (700 000–2 980 000)	1 240 000 (500 000–2 480 000)	20·6 (8·9–38·5)	186·9 (74·7–374·7)
African trypanosomiasis	82 600 (40 000–156 000)	4750 (810–19 400)	1·1 (0·5–2·0)	0·7 (0·1–2·9)
Ascariasis	794 000 (500 000–1 180 000)	216 000 (160 000–280 000)	10·3 (6·6–15·3)	32·6 (24·9–42·3)
*Bordetella* spp (pertussis)	11 500 000 (5 000 000–20 800 000)	10 200 000 (4 700 000–18 400 000)	148·7 (69·1–268·4)	1544·4 (711·9–2782·3)
*Campylobacter* spp	6 270 000 (2 000 000–12 500 000)	4 110 000 (1 700 000–8 160 000)	81·1 (31·2–161·3)	620·8 (253·1–1231·7)
Chagas disease	287 000 (190 000–481 000)	163 (85·0–367)	3·7 (2·5–6·2)	0·0 (0·0–0·1)
*Chlamydia* spp	5 580 000 (4 300 000–7 050 000)	4 250 000 (3 200 000–5 550 000)	72·1 (56·1–91·1)	641·0 (483·8–837·8)
*Citrobacter* spp	1 940 000 (1 200 000–2 870 000)	867 000 (530 000–1 360 000)	25·1 (15·6–37·1)	130·8 (79·4–204·6)
*Clostridioides difficile*	2 130 000 (1 300 000–3 460 000)	211 000 (120 000–333 000)	27·5 (16·7–44·7)	31·8 (18·1–50·3)
*Cryptosporidium* spp	6 310 000 (1 000 000–16 000 000)	5 040 000 (1 100 000–12 000 000)	81·5 (18·1–206·8)	759·7 (165·5–1814·3)
Cutaneous and mucocutaneous leishmaniasis	293 000 (190 000–437 000)	4830 (3000–7290)	3·8 (2·4–5·6)	0·7 (0·5–1·1)
Cystic echinococcosis	160 000 (120 000–211 000)	17 200 (5200–32 500)	2·1 (1·6–2·7)	2·6 (0·8–4·9)
Cysticercosis	1 390 000 (900 000–1 970 000)	1730 (17·0–4800)	17·9 (11·5–25·5)	0·3 (0·0–0·7)
Dengue virus	2 520 000 (900 000–3 440 000)	628 000 (160 000–904 000)	32·5 (11·2–44·4)	94·8 (24·1–136·4)
Diphtheria	859 000 (600 000–1 250 000)	723 000 (450 000–1 100 000)	11·1 (7·4–16·2)	109·1 (68·3–166·2)
Ebola virus	195 000 (160 000–231 000)	28 000 (23 000–33 000)	2·5 (2·1–3·0)	4·2 (3·5–5·0)
*Entamoeba histolytica*	2 290 000 (700 000–5 290 000)	1 430 000 (390 000–3 610 000)	29·6 (8·5–68·4)	215·0 (58·4–544·0)
*Enterobacter* spp	11 100 000 (7 000 000–16 000 000)	4 530 000 (3 200 000–6 410 000)	143·4 (96·5–207·2)	683·6 (478·1–966·6)
*Enterococcus faecalis*	6 980 000 (5 000 000–10 200 000)	2 000 000 (1 400 000–2 920 000)	90·2 (58·6–131·4)	301·4 (204·6–440·0)
*Enterococcus faecium*	6 000 000 (3 700 000–9 160 000)	996 000 (670 000–1 500 000)	77·6 (47·5–118·4)	150·3 (100·4–226·8)
Enteropathogenic *E coli*	1 300 000 (600 000–2 370 000)	1 040 000 (460 000–2 010 000)	16·8 (7·9–30·6)	156·2 (70·0–302·5)
Enterotoxigenic *E coli*	1 500 000 (600 000–2 930 000)	939 000 (370 000–1 990 000)	19·4 (8·4–37·9)	141·7 (56·3–300·1)
E coli*	28 500 000 (21 000 000–37 500 000)	10 600 000 (8 100 000–13 900 000)	367·9 (272·5–484·7)	1593·1 (1219·3–2096·4)
Food-borne trematodiases	780 000 (400 000–1450 000)	1670 (960–2720)	10·1 (5·0–18·7)	0·3 (0·1–0·4)
Fungi	18 500 000 (11 000 000–28 500 000)	14 300 000 (8 300 000–22 600 000)	239·5 (145·6–368·8)	2151·4 (1258·6–3412·1)
Genital herpes	253 000 (80 000–628 000)	0 (0–0)	3·3 (1·1–8·1)	0·0 (0·0–0·0)
Group A *Streptococcus* (*Streptococcus pyogenes*)	6 690 000 (4 000 000–10 900 000)	2 440 000 (1 700 000–3 490 000)	86·4 (54·1–140·3)	367·9 (251·3–527·3)
Group B *Streptococcus* (*Streptococcus agalactiae*)	11 200 000 (8 000 000–14 800 000)	7 000 000 (5 300 000–9 160 000)	144·8 (108·0–191·1)	1056·0 (795·3–1382·0)
Guinea worm disease	1·00 (0·00–1·00)	0 (0–0)	0·0 (0·0–0·0)	0·0 (0·0–0·0)
*Haemophilus influenzae*	5 100 000 (4 100 000–6 290 000)	3 740 000 (2 900 000–4 780 000)	65·9 (53·1–81·3)	565·0 (434·4–720·4)
*Helicobacter pylori*	16 400 000 (14 000 000–18 400 000)	0 (0–0)	211·8 (182·8–237·4)	0·0 (0·0–0·0)
Hepatitis A virus	3 310 000 (2 400 000–4 350 000)	737 000 (450 000–1130 000)	42·8 (31·2–56·3)	111·2 (68·2–170·2)
Hepatitis B virus	23 900 000 (21 000 000–27 000 000)	429 000 (260 000–652 000)	309·0 (271·5–348·9)	64·7 (38·8–98·3)
Hepatitis C virus	15 300 000 (13 000 000–17 500 000)	61 500 (29 000–99 600)	197·6 (171·9–225·9)	9·3 (4·3–15·0)
Hepatitis E virus	178 000 (110 000–264 000)	38 000 (19 000–64 500)	2·3 (1·4–3·4)	5·7 (2·9–9·7)
HIV or AIDS	52 100 000 (47 000 000–60 900 000)	4 890 000 (3 900 000–6 110 000)	673·4 (602·3–786·7)	737·3 (585·1–922·5)
Hookworm disease	984 000 (600 000–1470 000)	72 000 (46 000–107 000)	12·7 (8·1–19·0)	10·9 (7·0–16·1)
Human papillomavirus	9 600 000 (8 000 000–10 700 000)	0 (0–0)	124·1 (104·6–138·3)	0·0 (0·0–0·0)
Influenza virus	16 700 000 (14 000 000–20 100 000)	10 900 000 (8 500 000–13 900 000)	215·8 (179·0–259·9)	1646·8 (1275·3–2103·5)
Invasive non-typhoidal *Salmonella*	14 900 000 (9 000 000–22 900 000)	10 200 000 (6 400 000–15 300 000)	193·0 (115·5–296·3)	1545·1 (967·4–2308·2)
*Klebsiella pneumoniae*	31 100 000 (23 000 000–41 100 000)	17 200 000 (13 000 000–22 600 000)	401·8 (300·2–531·7)	2593·2 (1964·6–3402·1)
*Legionella* spp	3 220 000 (2 400 000–4 310 000)	1 710 000 (1 000 000–2 740 000)	41·6 (31·3–55·8)	257·9 (154·2–412·7)
Leprosy	28 800 (19 000–42 000)	0 (0–0)	0·4 (0·2–0·5)	0·0 (0·0–0·0)
*Listeria monocytogenes*	922 000 (600 000–1340 000)	544 000 (330 000–865 000)	11·9 (7·9–17·3)	82·1 (49·2–130·4)
Lymphatic filariasis	1 630 000 (1 000 000–2 710 000)	0 (0–0)	21·1 (12·4–35·1)	0·0 (0·0–0·0)
Malaria	53 600 000 (27 000 000–91 300 000)	37 200 000 (18 000 000–64 200 000)	693·3 (351·3–1180·0)	5606·4 (2686·1–9682·7)
Measles	9 440 000 (3 000 000–20 800 000)	7 940 000 (2 800 000–17 600 000)	122·0 (43·7–269·1)	1198·4 (417·2–2657·7)
*Morganella* spp	109 000 (70 000–164 000)	5040 (2500–9130)	1·4 (0·9–2·1)	0·8 (0·4–1·4)
*Mycoplasma* spp	4 950 000 (3 900 000–6 180 000)	3 490 000 (2 600 000–4 530 000)	64·0 (50·9–79·8)	526·6 (395·3–682·7)
*Neisseria gonorrhoeae*	231 000 (190 000–270 000)	0 (0–0)	3·0 (2·4–3·5)	0·0 (0·0–0·0)
*Neisseria meningitidis*	9 390 000 (6 000 000–13 500 000)	6 060 000 (4 200 000–8 700 000)	121·4 (83·9–174·2)	914·9 (629·4–1313·1)
Norovirus	6 560 000 (2 000 000–13 800 000)	3 090 000 (880 000–6 300 000)	84·8 (19·5–178·8)	466·1 (133·2–950·5)
Onchocerciasis	1 230 000 (800 000–1 820 000)	0 (0–0)	15·9 (9·9–23·5)	0·0 (0·0–0·0)
Other *Enterococcus* spp	2 930 000 (1 900 000–4 340 000)	1 140 000 (700 000–1 780 000)	37·9 (25·1–56·1)	172·2 (105·9–269·1)
Other *Klebsiella* spp	1 550 000 (800 000–2 770 000)	87 300 (34 000–180 000)	20·1 (10·3–35·8)	13·2 (5·2–27·2)
Other neglected tropical diseases	3 060 000 (2 100 000–5 110 000)	1 230 000 (790 000–2 870 000)	39·6 (27·0–66·0)	185·8 (118·5–432·9)
Other unspecified infectious diseases	5 080 000 (3 800 000–6 290 000)	1 820 000 (1 300 000–2 390 000)	65·7 (49·4–81·3)	274·1 (192·5–361·0)
Polymicrobial infections	11 900 000 (8 000 000–17 100 000)	8 210 000 (5 400 000–12 000 000)	153·7 (100·8–220·7)	1238·2 (807·9–1804·6)
*Proteus* spp	2 670 000 (1 800 000–3 880 000)	469 000 (310 000–693 000)	34·6 (22·6–50·1)	70·8 (46·5–104·5)
*Providencia* spp	120 000 (80 000–184 000)	7700 (3200–15 600)	1·6 (1·0–2·4)	1·2 (0·5–2·4)
*Pseudomonas aeruginosa*	18 300 000 (13 000 000–25 000 000)	8 210 000 (6 100 000–10 800 000)	236·4 (167·6–323·3)	1238·6 (913·0–1627·0)
Rabies	895 000 (400 000–1 230 000)	177 000 (59 000–297 000)	11·6 (4·7–15·8)	26·6 (8·8–44·8)
Respiratory syncytial virus	13 500 000 (10 000 000–17 000 000)	13 100 000 (10 000 000–16 600 000)	174·1 (135·5–220·2)	1972·4 (1525·8–2506·6)
Rotavirus	14 400 000 (7 000 000–24 100 000)	10 300 000 (5 200 000–16 700 000)	186·0 (94·6–311·2)	1558·4 (787·7–2519·1)
*Salmonella* Paratyphi	1 640 000 (700 000–3 230 000)	235 000 (73 000–568 000)	21·2 (8·8–41·7)	35·5 (11·0–85·8)
*Salmonella* Typhi	13 200 000 (9 000 000–19 800 000)	5 730 000 (3 700 000–8 520 000)	171·2 (111·0–255·3)	864·5 (562·0–1284·8)
Schistosomiasis	1 670 000 (1 100 000–2 670 000)	32 200 (20 000–52 200)	21·6 (13·9–34·5)	4·9 (3·0–7·9)
*Serratia* spp	4 010 000 (2 600 000–6 030 000)	2 010 000 (1 300 000–2 980 000)	51·9 (33·6–77·9)	302·9 (194·6–449·6)
*Shigella* spp	7 470 000 (3 000 000–13 400 000)	5 590 000 (2 300 000–10 400 000)	96·6 (43·4–172·6)	843·5 (353·9–1563·2)
*Staphylococcus aureus*	34 500 000 (26 000 000–45 500 000)	12 100 000 (9 400 000–15 400 000)	446·1 (336·6–588·6)	1822·2 (1423·3–2328·0)
*Streptococcus pneumoniae*	38 100 000 (32 000 000–46 500 000)	23 000 000 (18 000 000–28 900 000)	492·7 (408·3–601·6)	3471·8 (2779·4–4357·2)
Syphilis	9 540 000 (3 000 000–19 400 000)	9 240 000 (3 200 000–19 000 000)	123·3 (43·9–250·4)	1394·6 (478·6–2861·0)
Tetanus	2 610 000 (2 000 000–3 710 000)	1 870 000 (1 400 000–2 730 000)	33·8 (25·7–48·0)	281·4 (207·5–411·2)
Trachoma	181 000 (110 000–274 000)	0 (0–0)	2·3 (1·5–3·5)	0·0 (0·0–0·0)
Trichomoniasis	287 000 (110 000–592 000)	0 (0–0)	3·7 (1·5–7·7)	0·0 (0·0–0·0)
Trichuriasis	236 000 (130 000–402 000)	13 300 (7300–22 100)	3·0 (1·6–5·2)	2·0 (1·1–3·3)
Tuberculosis	65 100 000 (59 000 000–71 200 000)	6 250 000 (4 900 000–7 840 000)	840·8 (766·9–920·8)	943·1 (743·4–1183·0)
Varicella-zoster virus	1 430 000 (1 200 000–1 650 000)	537 000 (430 000–661 000)	18·5 (16·1–21·4)	81·0 (65·0–99·7)
*Vibrio cholerae*	6 750 000 (4 000 000–11 600 000)	3 420 000 (1 700 000–5 840 000)	87·3 (47·1–149·5)	515·6 (259·1–880·5)
Viral meningitis	1 950 000 (1 400 000–2 640 000)	840 000 (570 000–1 210 000)	25·2 (18·6–34·1)	126·8 (86·1–183·1)
Visceral leishmaniasis	436 000 (100 000–1420 000)	139 000 (48 000–411 000)	5·6 (1·8–18·4)	20·9 (62·1–7·2)
Yellow fever	301 000 (110 000–618 000)	44 000 (15 000–98 300)	3·9 (1·4–8·0)	6·6 (2·3–14·8)
Zika virus	347 (260–455)	119 (72·0–195)	0·0 (0·0–0·0)	0·0 (0·0–0·0)
**Total**	**704 000 000 (610 000 000–820 000 000)**	**309 000 000 (250 000 000–377 000 000)**	**9103·9 (7885·4–10 599·6)**	**46 542·6 (38 431·1–56 888·1)**

95% uncertainty intervals are shown in parentheses. Counts are shown to three significant figures and rates are shown to one decimal place. DALYs=disability-adjusted life-years. *E coli*=*Escherichia coli*. *Salmonella* Typhi=*Salmonella enterica* serotype Typhi. *Salmonella* Paratyphi=*Salmonella enterica* serotype Paratyphi.

* Excluding enteropathogenic and enterotoxigenic *E coli*.

Comparing super-regions, we observed substantial differences in estimated pathogen-associated burdens in relation to DALYs from all causes in 2019.^1^ The highest fraction of pathogen-associated DALYs among the overall DALY burden per region was observed in sub-Saharan Africa (314 million [95% UI 270–368; 61·5% of 511 million total DALYs), and the lowest was observed in the high-income super-region (31·8 million [25·4–40·1; 9·8% of 324 million total DALYs; figure 1, appendix pp 39–46). The ranking of regions according to fraction of overall DALY burden that was associated with the 85 pathogens (from highest to lowest) was: sub-Saharan Africa; south Asia; north Africa and the Middle East; Latin America and the Carribean; southeast Asia, east Asia, and Oceania; central Europe, eastern Europe, and central Asia; and high-income (figure 1).

In children younger than 5 years, the highest fraction of pathogen-associated DALYs among the overall DALY burden per region was observed in sub-Saharan Africa (193 million [95% UI 158–237]; 79·3% of 244 million total DALYs in under 5s), and the lowest was observed in the high-income super-region (1·06 million [0·754–1·46]; 16·5% of 6·41 million total DALYs in under 5s; figure 1, appendix pp 39, 47–53). The ranking of regions according to fraction of overall DALY burden associated with the 85 pathogens in children younger than 5 years (from highest to lowest) was: sub-Saharan Africa; south Asia; southeast Asia, east Asia, and Oceania; north Africa and the Middle East; Latin America and the Carribean; central Europe, eastern Europe, and central Asia; and high-income (figure 1).

**Figure 2:**
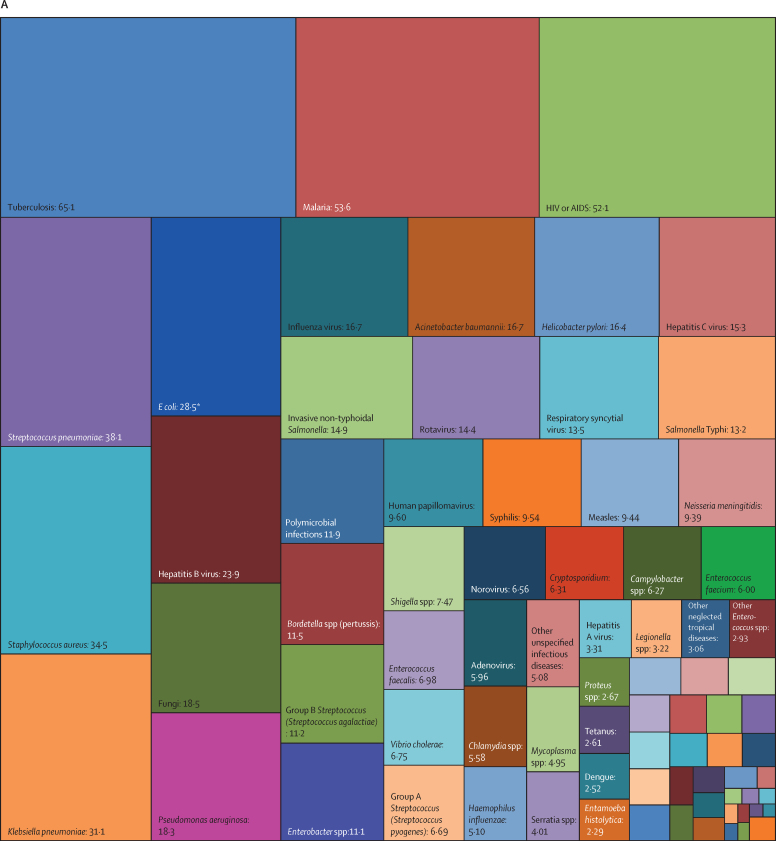

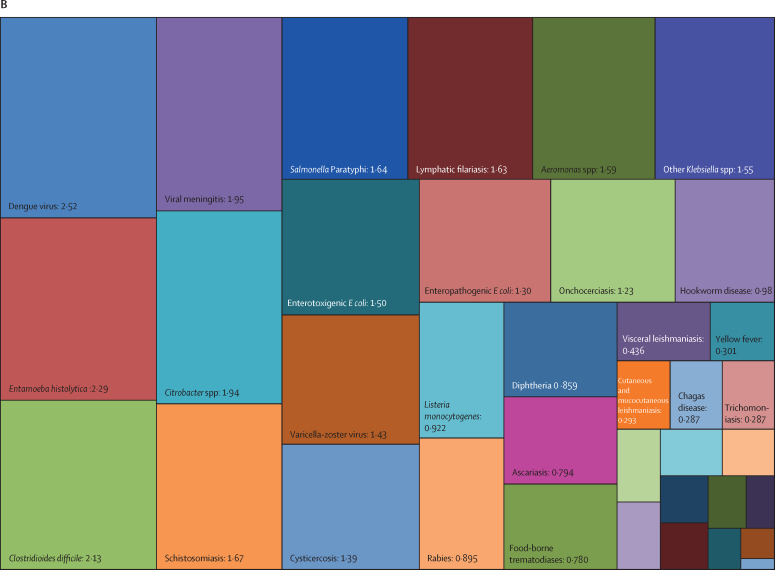

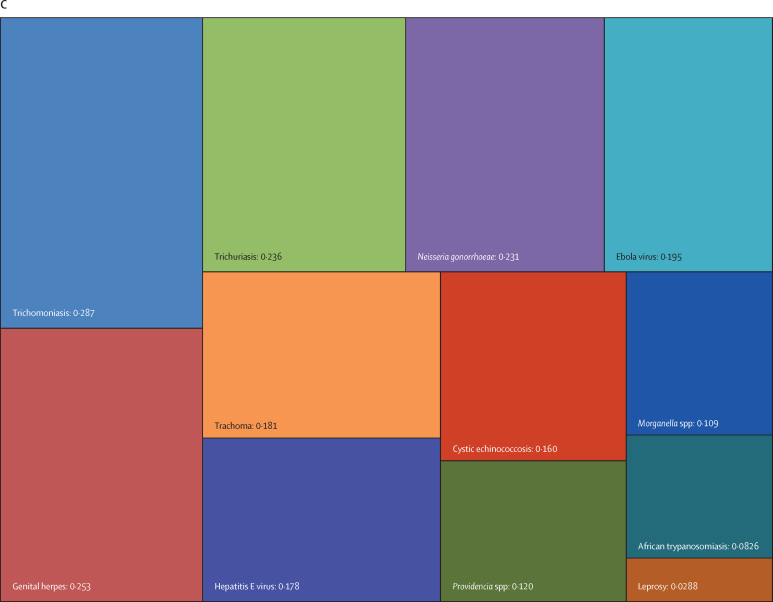
Treemap of global DALYs associated with specific pathogens for all age groups, 2019 DALYs are shown as counts in units of a million, presented to three significant figures. Panel A shows all components; panels B and C represent an enlarged view of the lower-right portion of panels A and B, respectively. Colour schemes across panels A–C are independent of each other. 95% UIs are presented in table 1. DALYs=disability-adjusted life-years. *E coli*=Escherichia coli. *Salmonella* Typhi=*Salmonella enterica* serotype Typhi. *Salmonella* Paratyphi=*Salmonella enterica* serotype Paratyphi. *Excluding enteropathogenic and enterotoxigenic *E coli*.

Overall, three pathogens were responsible for more than 50 million DALYs each in 2019: tuberculosis (65·1 million [95% UI 59·0–71·2]), malaria (53·6 million [27·0–91·3]), and HIV or AIDS (52·1 million [47·0–60·9]). The next most burdensome pathogens (all bacteria), with more than 30 million DALYs each in 2019, were: *Streptococcus pneumoniae* (38·1 million [32·0–46·5]), *S aureus* (34·5 million [26·0–45·5]), and *K pneumoniae* (31·1 million [23·0–41·1]; table 1, figure 2). Others among the top 20 most burdensome pathogens were *Escherichia coli*, hepatitis B and C virus, *Pseudomonas aeruginosa*, influenza virus, *Acinetobacter baumannii, H pylori*, invasive non-typhoidal *Salmonella*, rotavirus, respiratory syncytial virus, *Salmonella enterica* serovar Typhi, *Bordetella* spp (pertussis), group B *Streptococcus* (*Streptococcus agalactiae*), and fungi as a group (table 1, figure 2).

**Figure 3:**
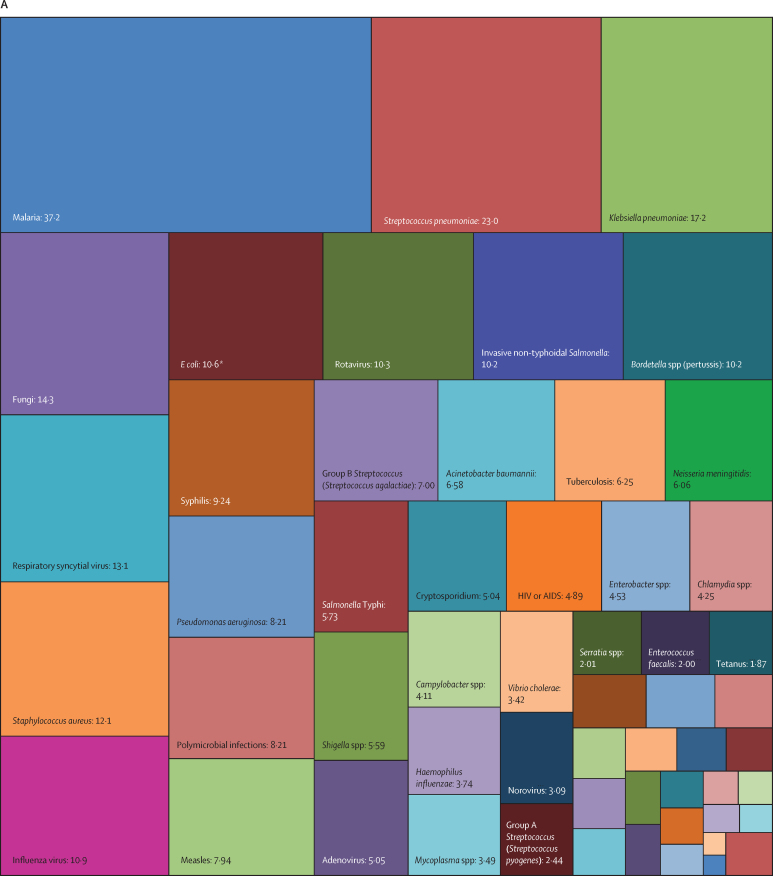

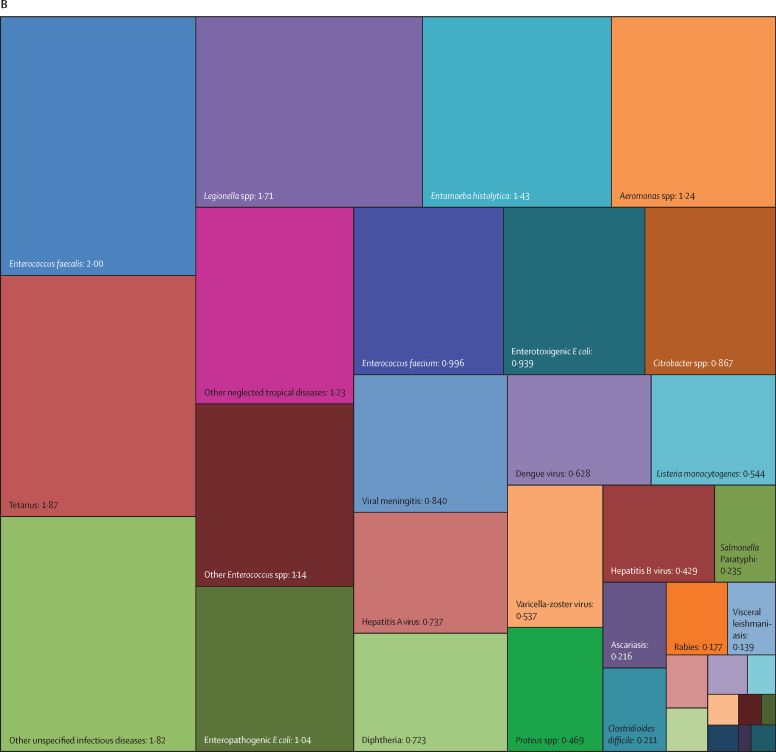

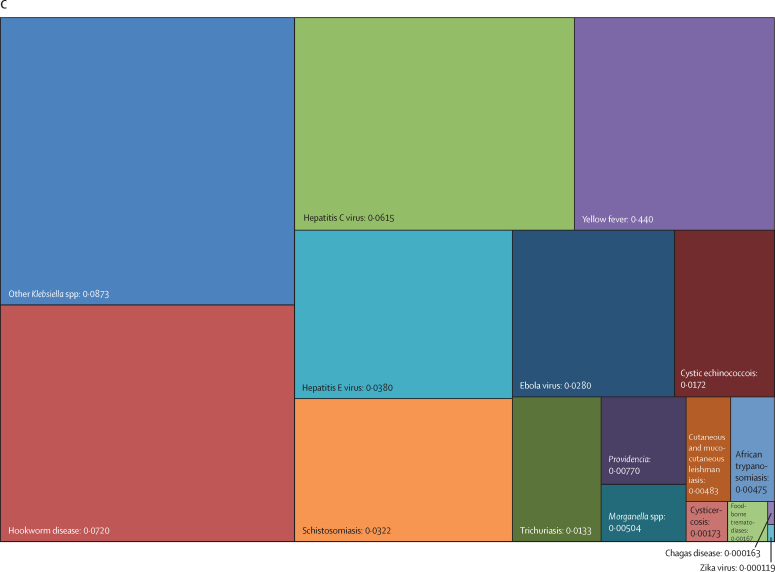
Treemap of global DALYs associated with specific pathogens for children younger than 5 years, 2019 DALYs are shown as counts in units of a million, presented to three significant figures. Panel A shows all components; panels B and C represent an enlarged view of the lower-right portion of panels A and B, respectively. Colour schemes across panels A–C are independent of each other. 95% UIs are presented in table 1. DALYs=disability-adjusted life-years. *E coli*=Escherichia coli. *Salmonella* Typhi=*Salmonella enterica* serotype Typhi. *Salmonella* Paratyphi=*Salmonella enterica* serotype Paratyphi. *Excluding enteropathogenic and enterotoxigenic *E coli*.

In children younger than 5 years, malaria (which includes *Plasmodium falciparum* and other *Plasmodium* spp) was responsible for the highest burden (37·2 million [95% UI 18·0–64·2] DALYs), followed by infections due to *S pneumoniae* and *K pneumoniae*, which accounted for 23·0 million (18·0–28·9) DALYs and 17·2 million (13·0–22·6) DALYs, respectively (table 1, figure 3). For the remaining top 10 pathogens according to respective associated burden in children younger than 5 years, fungi as a group ranked fourth, followed by respiratory syncytial virus, *S aureus*, influenza virus, *E coli*, rotavirus, and invasive non-typhoidal *Salmonella*. Of note, the burden of syphilis surpassed the burden of tuberculosis and HIV or AIDS, with syphilis ranked 12th, and tuberculosis and HIV or AIDS ranked 18th and 24th, respectively.

We observed the greatest age-standardised DALY rates for tuberculosis (840·8 [95% UI 766·9–920·8] per 100 000 population), malaria (693·3 [351·3–1180·0] per 100 000 population), and HIV or AIDS (673·4 [602·3–786·7] per 100 000 population). In children younger than 5 years, the highest rates were seen for malaria (5606·4 [2686·1–9682·7] per 100 000 population) and infections due to *S pneumoniae* (3471·8 [2779·4–4357·2] per 100 000 population) and *K pneumoniae* (2593·2 [1964·6–3402·1] per 100 000 population; table 1). For all ages, the lowest age-standardised DALY rate was observed for Guinea worm disease globally and across all super-regions (table 1, table 2), which has almost been eradicated worldwide. The age-standardised rates were low for many other well known parasitic diseases, such as cystic echinococcosis and African trypanosomiasis (table 1).

**Table 2:** Age-standardised DALY rate per 100 000 population associated with specific pathogens in each GBD super-region for all ages and children younger than 5 years, 2019

	**Central Europe, eastern Europe, and central Asia**		**High income**		**Latin America and Caribbean**		**North Africa and Middle East**		**South Asia**		**Southeast Asia, east Asia, and Oceania**		**Sub-Saharan Africa**	
	All ages	Age <5 years	All ages	Age <5 years	All ages	Age <5 years	All ages	Age <5 years	All ages	Age <5 years	All ages	Age <5 years	All ages	Age <5 years
*Acinetobacter baumannii*	100·9 (59·6–160·0)	197·6 (124·7–304·5)	71·8 (42·4–113·2)	53·3 (30·8–83·6)	146·3 (90·7–219·4)	458·6 (294·2–680·3)	160·2 (97·7–244·9)	630·6 (383·8–972·1)	278·4 (182·5–408·5)	1432·1 (864·1–2202·8)	200·7 (120·4–304·6)	413·0 (271·8–607·5)	399·3 (274·5–568·6)	1787·8 (1136·9–2724·9)
Adenovirus	2·5 (1·4–4·4)	19·7 (9·3–40·3)	2·8 (1·6–4·6)	17·8 (8·2–33·9)	12·1 (6·7–22·4)	120·6 (59·3–238·9)	32·1 (14·2–67·5)	292·4 (118·9–632·2)	75·7 (41·8–130·3)	563·0 (274·6–1067·5)	8·4 (4·7–14·8)	97·0 (47·3–185·9)	380·4 (199·2–646·8)	2256·0 (1126·4–3944·4)
*Aeromonas* spp	1·4 (0·7–2·5)	10·4 (3·9–21·6)	0·2 (0·1–0·3)	0·7 (0·3–1·5)	2·6 (1·2–5·0)	19·7 (7·3–43·2)	9·2 (3·4–19·3)	74·7 (24·3–172·3)	26·5 (12·1–47·6)	199·4 (81·8–373·4)	2·0 (0·8–3·8)	11·1 (4·0–23·3)	92·0 (36·9–178·2)	505·5 (187·6–1034·1)
African trypanosomiasis	0·0 (0·0–0·0)	0·0 (0·0–0·0)	0·0 (0·0–0·0)	0·0 (0·0–0·0)	0·0 (0·0–0·0)	0·0 (0·0–0·0)	0·0 (0·0–0·0)	0·0 (0·0–0·0)	0·0 (0·0–0·0)	0·0 (0·0–0·0)	0·0 (0·0–0·0)	0·0 (0·0–0·0)	7·7 (3·5–14·4)	2·9 (0·5–11·7)
Ascariasis	0·2 (0·1–0·3)	0·6 (0·3–1·0)	0·0 (0·0–0·0)	0·1 (0·0–0·1)	6·3 (3·8–10·2)	11·6 (7·9–17·1)	4·0 (2·5–6·2)	9·9 (6·7–14·2)	20·6 (11·7–34·3)	28·7 (18·5–44·0)	4·2 (2·5–6·7)	8·7 (6·1–12·7)	24·9 (18·3–33·0)	87·3 (64·6–115·2)
*Bordetella* spp (pertussis)	8·0 (3·1–16·7)	107·4 (42·7–227·6)	1·1 (0·7–1·6)	18·8 (12·6–27·4)	34·8 (15·8–68·0)	377·5 (169·5–729·1)	120·8 (45·2–259·0)	1074·0 (403·8–2329·5)	143·2 (36·8–329·4)	1360·0 (356·3–3088·1)	46·5 (19·2–93·0)	623·1 (260·7–1240·5)	643·2 (261·8–1275·7)	3780·1 (1546·6–7582·8)
*Campylobacter* spp	13·7 (3·9–30·4)	55·7 (20·2–123·3)	6·9 (1·6–15·3)	12·4 (4·6–26·3)	23·1 (8·6–47·3)	164·5 (64·1–338·8)	12·8 (4·1–29·7)	94·8 (28·4–236·2)	139·1 (53·6–285·5)	895·6 (387·5–1684·2)	14·1 (4·5–33·9)	88·8 (32·3–200·0)	288·7 (110·7–593·4)	1423·3 (541·9–2952·5)
Chagas disease	0·0 (0·0–0·0)	0·0 (0·0–0·0)	2·8 (1·8–6·6)	0·1 (0·0–0·2)	44·0 (29·1–72·2)	0·2 (0·1–0·6)	0·0 (0·0–0·0)	0·0 (0·0–0·0)	0·0 (0·0–0·0)	0·0 (0·0–0·0)	0·0 (0·0–0·0)	0·0 (0·0–0·0)	0·0 (0·0–0·0)	0·0 (0·0–0·0)
*Chlamydia* spp	22·0 (18·0–27·2)	128·0 (98·6–167·3)	8·8 (7·1–11·2)	10·8 (6·0–17·8)	30·7 (23·6–39·9)	180·7 (121·1–256·3)	47·7 (35·0–62·9)	366·9 (257·3–504·1)	101·3 (77·1–129·5)	868·0 (630·5–1161·8)	24·6 (18·7–31·8)	177·5 (129·8–232·6)	237·4 (184·5–307·1)	1342·3 (1020·6–1767·0)
*Citrobacter* spp	23·5 (12·5–40·8)	38·0 (19·6–67·5)	10·7 (6·8–15·9)	11·2 (6·1–18·7)	19·7 (12·7–29·0)	84·8 (53·4–131·0)	18·2 (10·4–28·7)	84·6 (47·7–136·9)	32·5 (19·4–49·8)	184·1 (107·1–299·5)	21·5 (12·6–33·5)	76·5 (47·2–117·5)	42·1 (25·4–64·6)	210·4 (123·7–336·0)
*Clostridioides difficile*	59·7 (35·2–88·6)	40·6 (21·0–68·9)	59·4 (42·3–79·3)	65·2 (40·5–101·5)	32·7 (19·6–49·4)	115·8 (58·1–202·6)	18·2 (6·9–42·9)	17·3 (7·2–35·2)	6·2 (2·3–14·4)	8·6 (3·6–18·0)	32·3 (15·2–69·6)	39·5 (22·0–67·2)	11·1 (5·5–20·4)	16·2 (7·2–32·1)
*Cryptosporidium* spp	3·8 (0·7–12·4)	32·6 (5·1–104·0)	1·3 (0·2–4·2)	2·7 (0·4–9·1)	8·8 (1·4–28·4)	82·3 (12·8–271·4)	30·5 (5·1–88·8)	291·2 (48·9–834·6)	72·4 (12·3–234·3)	411·4 (72·3–1203·9)	3·9 (0·6–13·5)	32·5 (5·0–109·5)	430·8 (100·0–1024·7)	2467·7 (555·5–5641·7)
Cutaneous and mucocutaneous leishmaniasis	0·1 (0·0–0·1)	0·1 (0·0–0·1)	0·0 (0·0–0·0)	0·0 (0·0–0·0)	2·2 (1·4–3·3)	1·1 (0·6–1·8)	39·9 (25·1–59·6)	6·7 (4·0–10·2)	1·6 (1·0–2·4)	0·1 (0·1–0·2)	0·0 (0·0–0·0)	0·0 (0·0–0·0)	0·8 (0·5–1·1)	0·1 (0·0–0·1)
Cystic echinococcosis	9·9 (5·8–16·2)	0·5 (0·1–1·3)	0·3 (0·2–0·4)	0·0 (0·0–0·1)	0·2 (0·1–0·2)	0·1 (0·0–0·2)	5·6 (4·1–7·4)	2·5 (0·7–4·9)	2·0 (1·5–2·6)	1·6 (0·3–3·4)	0·5 (0·3–0·6)	0·2 (0·0–0·5)	3·2 (2·0–4·6)	7·6 (2·5–13·9)
Cysticercosis	24·1 (14·1–37·2)	0·0 (0·0–0·0)	11·4 (6·7–17·7)	0·0 (0·0–0·0)	49·7 (31·8–70·3)	0·2 (0·0–0·6)	0·1 (0·1–0·2)	0·0 (0·0–0·0)	21·6 (13·1–31·5)	0·0 (0·0–0·0)	9·8 (5·5–15·2)	0·0 (0·0–0·0)	25·0 (16·1–35·6)	1·0 (0·0–2·7)
Dengue virus	0·0 (0·0–0·0)	0·0 (0·0–0·0)	0·3 (0·1–0·5)	0·2 (0·0–0·5)	20·7 (15·5–24·8)	44·8 (31·2–55·4)	2·0 (0·8–3·9)	4·4 (3·1–6·2)	69·8 (15·6–105·7)	111·3 (21·8–178·5)	49·4 (21·5–61·5)	294·1 (73·2–428·3)	4·9 (1·2–10·6)	4·9 (0·7–10·4)
Diphtheria	0·2 (0·1–0·2)	0·5 (0·2–0·9)	0·0 (0·0–0·0)	0·1 (0·1–0·2)	0·4 (0·2–0·5)	2·3 (1·3–4·1)	1·7 (1·1–2·6)	9·4 (4·7–17·0)	1·7 (1·2–2·2)	9·3 (5·8–14·1)	0·6 (0·5–0·8)	4·4 (3·0–6·2)	74·3 (48·2–109·6)	419·2 (258·2–644·4)
Ebola virus	0·0 (0·0–0·0)	0·0 (0·0–0·0)	0·0 (0·0–0·0)	0·0 (0·0–0·0)	0·0 (0·0–0·0)	0·0 (0·0–0·0)	0·0 (0·0–0·0)	0·0 (0·0–0·0)	0·0 (0·0–0·0)	0·0 (0·0–0·0)	0·0 (0·0–0·0)	0·0 (0·0–0·0)	18·1 (14·8–21·4)	16·9 (13·8–19·9)
*Entamoeba histolytica*	3·6 (1·1–8·7)	16·7 (4·1–44·0)	0·5 (0·1–1·1)	1·5 (0·4–4·2)	15·0 (4·6–34·2)	121·6 (34·0–288·1)	22·2 (5·9–57·6)	182·8 (44·7–495·6)	50·5 (15·0–124·7)	252·2 (66·3–618·3)	1·5 (0·4–3·9)	11·4 (2·7–30·9)	102·0 (28·4–243·3)	495·6 (124·1–1276·1)
*Enterobacter* spp	97·8 (62·3–143·2)	177·2 (120·5–263·6)	64·9 (43·3–92·3)	58·8 (36·1–91·8)	113·0 (76·3–162·1)	425·1 (280·6–617·3)	109·7 (68·7–165·3)	467·0 (296·7–694·7)	178·6 (118·1–258·8)	1065·6 (712·9–1569·3)	147·3 (91·4–223·8)	413·5 (276·1–599·1)	208·5 (151·3–288·2)	985·2 (700·4–1374·7)
*Enterococcus faecalis*	127·5 (76·8–192·9)	89·3 (58·7–136·7)	75·5 (46·6–113·1)	36·3 (22·7–56·6)	93·9 (61·0–137·2)	217·0 (151·7–307·7)	62·1 (36·1–97·8)	178·9 (110·7–277·7)	98·7 (63·3–145·7)	368·3 (232·4–561·9)	61·8 (36·7–95·5)	105·7 (72·9–153·6)	146·8 (97·0–209·4)	595·7 (409·9–857·7)
*Enterococcus faecium*	123·4 (73·8–188·7)	56·5 (36·2–88·7)	88·3 (55·2–131·4)	32·7 (19·8–51·5)	89·3 (55·5–132·5)	148·9 (99·9–220·1)	59·7 (34·7–94·7)	111·5 (65·8–177·3)	70·5 (40·9–112·4)	164·1 (102·8–255·7)	64·9 (38·3–103·3)	67·2 (44·4–100·5)	90·0 (56·2–138·3)	277·5 (183·3–413·8)
Enteropathogenic *E coli*	0·9 (0·4–1·6)	5·5 (2·5–10·7)	0·3 (0·1–0·7)	1·5 (0·6–3·2)	1·3 (0·6–2·4)	11·0 (4·7–21·4)	8·3 (3·7–16·1)	74·7 (31·0–148·7)	16·3 (7·5–29·7)	110·1 (47·8–215·4)	2·5 (1·1–4·6)	26·5 (11·2–53·7)	82·4 (38·7–156·8)	461·6 (205·9–906·0)
Enterotoxigenic *E coli*	3·6 (1·6–6·9)	20·5 (8·2–44·7)	2·1 (1·0–3·9)	6·9 (2·7–14·0)	1·8 (0·8–3·7)	14·7 (5·5–32·6)	13·6 (5·4–30·3)	110·5 (40·5–263·6)	43·2 (18·5–84·9)	228·9 (89·1–482·4)	3·8 (1·6–7·5)	32·8 (11·4–73·4)	46·7 (18·6–99·9)	262·0 (99·0–596·6)
E coli*	478·4 (308·4–703·6)	453·5 (344·2–599·3)	318·6 (217·6–451·9)	132·1 (86·6–197·1)	321·3 (230·2–440·2)	715·5 (496·9–989·4)	244·0 (167·4–345·1)	993·5 (712·9–1355·1)	403·9 (302·3–523·5)	1853·9 (1369·0–2470·5)	201·3 (135·9–289·1)	488·6 (369·6–634·9)	743·1 (569·4–971·0)	3431·6 (2569·5–4621·0)
Food-borne trematodiases	4·2 (1·8–7·6)	0·1 (0·1–0·2)	2·2 (1·4–3·2)	0·1 (0·0–0·2)	6·0 (2·0–11·6)	0·1 (0·0–0·1)	2·7 (0·6–5·5)	0·0 (0·0–0·1)	0·0 (0·0–0·0)	0·0 (0·0–0·0)	31·8 (14·6–60·4)	1·1 (0·6–1·8)	0·0 (0·0–0·0)	0·0 (0·0–0·0)
Fungi	60·9 (38·6–93·2)	214·8 (120·8–353·6)	25·2 (18·1–35·0)	39·7 (21·8–67·0)	113·3 (74·2–163·5)	763·9 (458·8–1205·7)	138·0 (77·7–219·7)	999·4 (524·0–1676·8)	301·2 (171·4–475·3)	2473·3 (1306·0–4200·5)	69·2 (44·2–101·6)	478·0 (289·9–755·3)	887·2 (540·9–1364·8)	5114·5 (3036·7–7991·5)
Genital herpes	2·9 (0·9–7·2)	0·0 (0·0–0·0)	3·4 (1·1–8·3)	0·0 (0·0–0·0)	5·9 (1·9–14·5)	0·0 (0·0–0·0)	2·5 (0·8–6·1)	0·0 (0·0–0·0)	1·8 (0·6–4·5)	0·0 (0·0–0·0)	3·3 (1·0–8·2)	0·0 (0·0–0·0)	4·7 (1·6–11·4)	0·0 (0·0–0·0)
Group A *Streptococcus* (*Streptococcus pyogenes*)	98·9 (55·0–168·2)	166·8 (109·5–251·7)	63·6 (37·9–105·0)	78·5 (49·6–116·4)	91·8 (59·4–143·0)	343·2 (234·5–483·2)	68·5 (39·0–114·7)	280·2 (172·2–423·7)	88·4 (54·0–146·2)	395·4 (260·3–597·3)	60·8 (32·8–109·2)	155·5 (105·4–223·9)	159·8 (106·8–236·5)	692·0 (466·2–999·5)
Group B *Streptococcus* (*Streptococcus pyogenes*)	85·3 (57·9–122·3)	274·0 (203·0–363·7)	65·7 (44·3–94·2)	76·1 (49·0–112·4)	101·6 (70·3–140·5)	509·2 (345·4–705·2)	106·1 (70·1–152·1)	669·8 (447·5–934·9)	169·0 (123·0–223·0)	1346·2 (965·0–1791·9)	74·6 (49·3–107·4)	353·7 (257·6–470·3)	392·7 (302·1–510·5)	2127·1 (1609·7–2805·4)
Guinea worm disease	0·0 (0·0–0·0)	0·0 (0·0–0·0)	0·0 (0·0–0·0)	0·0 (0·0–0·0)	0·0 (0·0–0·0)	0·0 (0·0–0·0)	0·0 (0·0–0·0)	0·0 (0·0–0·0)	0·0 (0·0–0·0)	0·0 (0·0–0·0)	0·0 (0·0–0·0)	0·0 (0·0–0·0)	0·0 (0·0–0·0)	0·0 (0·0–0·0)
*Haemophilus influenzae*	30·7 (25·7–36·9)	159·9 (127·8–199·9)	15·0 (12·4–18·6)	15·5 (11·5–20·7)	33·4 (26·0–42·3)	190·0 (138·7–250·8)	42·2 (32·5–54·7)	309·2 (231·1–412·7)	75·2 (58·1–94·6)	623·8 (455·8–815·5)	26·2 (20·8–33·0)	195·7 (154·2–244·0)	225·5 (173·8–290·5)	1276·4 (961·1–1670·5)
*Helicobacter pylori*	324·2 (282·0–361·1)	0·0 (0·0–0·0)	201·0 (175·7–220·3)	0·0 (0·0–0·0)	198·3 (168·9–228·8)	0·0 (0·0–0·0)	122·8 (103·8–141·5)	0·0 (0·0–0·0)	114·5 (95·4–134·2)	0·0 (0·0–0·0)	378·1 (309·9–445·6)	0·0 (0·0–0·0)	66·2 (55·0–77·5)	0·0 (0·0–0·0)
Hepatitis A virus	4·3 (3·2–5·6)	7·4 (4·1–11·1)	2·8 (2·0–3·8)	1·2 (0·9–1·7)	6·7 (5·2–8·2)	11·1 (8·2–14·6)	28·6 (12·5–52·2)	62·5 (23·0–120·7)	129·4 (85·4–188·0)	379·4 (214·6–606·6)	10·9 (6·7–16·1)	5·9 (4·2–8·0)	44·2 (26·6–76·4)	36·0 (18·5–69·5)
Hepatitis B virus	289·1 (233·1–352·1)	21·0 (15·4–28·4)	112·7 (98·0–129·5)	1·1 (0·8–1·4)	86·3 (73·6–100·5)	8·0 (5·0–10·9)	222·7 (165·4–292·1)	38·2 (14·8–80·4)	367·3 (304·9–443·9)	167·9 (57·6–305·9)	444·6 (378·4–513·7)	12·1 (9·1–14·9)	314·5 (251·4–388·8)	62·2 (39·5–100·0)
Hepatitis C virus	254·3 (209·6–308·2)	1·8 (1·2–2·8)	245·5 (220·5–273·1)	0·4 (0·3–0·5)	176·6 (149·9–210·0)	1·9 (1·3–2·9)	251·5 (184·5–321·8)	3·1 (1·7–6·0)	178·8 (148·9–212·2)	26·3 (7·8–47·8)	196·6 (168·5–227·3)	1·4 (1·0–2·1)	142·0 (114·0–174·9)	7·8 (3·5–14·1)
Hepatitis E virus	0·5 (0·4–0·7)	1·5 (0·4–3·1)	0·3 (0·2–0·4)	0·2 (0·1–0·3)	0·6 (0·4–0·8)	1·2 (0·7–1·8)	1·1 (0·7–2·5)	2·0 (0·6–4·5)	5·8 (3·1–9·8)	18·4 (8·6–34·5)	1·7 (1·0–2·2)	0·9 (0·6–1·3)	2·1 (1·3–3·8)	2·6 (1·0–5·5)
HIV or AIDS	404·4 (390·4–422·5)	160·2 (156·7–163·7)	77·2 (66·9–91·3)	14·1 (13·4–15·0)	414·5 (386·1–463·6)	355·7 (248·0–596·9)	91·8 (52·6–184·7)	108·0 (33·3–422·4)	177·4 (141·1–283·4)	157·8 (85·8–474·4)	205·2 (177·9–240·4)	231·0 (160·0–335·4)	3613·5 (3105·8–4398·0)	2423·9 (1860·6–3119·8)
Hookworm disease	0·8 (0·5–1·2)	0·4 (0·2–0·6)	0·2 (0·1–0·3)	0·1 (0·0–0·2)	7·8 (4·8–11·7)	3·9 (2·3–6·2)	3·9 (2·4–6·1)	2·4 (1·4–3·6)	12·8 (7·8–19·7)	6·7 (4·0–10·4)	6·3 (3·8–10·1)	4·3 (2·5–7·0)	50·2 (31·8–75·1)	31·1 (19·8–46·1)
Human papillomavirus	158·8 (137·1–180·1)	0·0 (0·0–0·0)	78·9 (69·3–84·1)	0·0 (0·0–0·0)	192·0 (168·6–222·0)	0·0 (0·0–0·0)	39·1 (29·8–47·2)	0·0 (0·0–0·0)	109·0 (87·1–140·9)	0·0 (0·0–0·0)	125·6 (88·1–150·6)	0·0 (0·0–0·0)	189·8 (145·9–233·9)	0·0 (0·0–0·0)
Influenza virus	111·7 (99·7–126·4)	566·0 (455·4–708·9)	96·0 (85·2–104·9)	50·2 (37·3–67·8)	151·9 (131·0–176·6)	674·1 (489·8–888·2)	145·6 (116·2–177·9)	988·3 (738·2–1284·9)	247·8 (195·3–308·3)	1973·3 (1474·8–2574·7)	86·4 (73·4–102·1)	545·5 (427·5–681·2)	656·5 (517·0–833·8)	3503·1 (2661·9–4569·2)
Invasive non-typhoidal *Salmonella*	38·4 (17·4–72·2)	115·0 (58·7–208·0)	14·7 (3·3–38·4)	35·2 (10·8–85·6)	49·0 (22·5–95·6)	314·6 (171·9–526·2)	92·7 (47·9–178·4)	546·3 (288·4–989·4)	140·3 (85·1–223·1)	852·0 (547·3–1272·3)	47·9 (25·2–89·3)	300·5 (174·9–485·4)	945·5 (558·8–1476·9)	4760·5 (2808·9–7321·0)
*Klebsiella pneumoniae*	285·8 (195·3–404·4)	642·0 (487·2–854·4)	170·4 (118·7–239·7)	138·6 (89·7–204·3)	307·0 (217·6–418·9)	1261·0 (878·2–1747·9)	281·7 (191·7–402·0)	1543·3 (1083·6–2158·0)	467·4 (340·6–632·0)	2993·2 (2169·4–4061·6)	195·6 (136·8–272·6)	812·1 (610·8–1056·9)	1101·1 (850·8–1426·3)	5637·3 (4254·7–7317·9)
*Legionella* spp	33·3 (28·8–38·9)	83·4 (56·5–120·8)	29·3 (25·1–34·5)	12·4 (6·2–22·5)	30·9 (25·2–38·5)	106·9 (61·8–172·2)	30·6 (21·8–42·3)	173·6 (99·0–282·9)	51·2 (35·1–73·9)	387·3 (225·1–627·8)	23·0 (18·2–29·6)	94·7 (58·6–146·8)	90·3 (60·7–134·3)	455·2 (264·7–736·6)
Leprosy	0·0 (0·0–0·0)	0·0 (0·0–0·0)	0·0 (0·0–0·0)	0·0 (0·0–0·0)	0·5 (0·3–0·7)	0·0 (0·0–0·0)	0·1 (0·1–0·1)	0·0 (0·0–0·0)	0·9 (0·6–1·3)	0·0 (0·0–0·0)	0·1 (0·1–0·2)	0·0 (0·0–0·0)	0·6 (0·4–0·8)	0·0 (0·0–0·0)
*Listeria monocytogenes*	2·9 (1·9–4·9)	10·0 (5·8–15·9)	1·4 (0·9–2·4)	4·1 (2·5–6·2)	4·1 (2·6–6·2)	23·0 (13·3–35·8)	6·7 (3·7–12·1)	35·3 (16·2–68·1)	11·3 (7·8–16·1)	60·1 (36·6–93·9)	3·3 (2·2–5·1)	21·9 (13·6–32·9)	51·4 (32·1–78·7)	227·7 (131·5–381·1)
Lymphatic filariasis	0·0 (0·0–0·0)	0·0 (0·0–0·0)	0·0 (0·0–0·0)	0·0 (0·0–0·0)	3·1 (1·9–5·0)	0·0 (0·0–0·0)	3·7 (2·0–6·8)	0·0 (0·0–0·0)	48·3 (28·4–80·0)	0·0 (0·0–0·0)	13·7 (8·1–22·7)	0·0 (0·0–0·0)	38·9 (23·0–65·2)	0·0 (0·0–0·0)
Malaria	0·0 (0·0–0·0)	0·0 (0·0–0·0)	0·0 (0·0–0·0)	0·0 (0·0–0·0)	16·7 (6·5–35·7)	33·9 (12·1–77·6)	90·2 (27·9–207·6)	203·8 (73·4–445·0)	159·2 (64·1–367·5)	653·1 (269·2–1390·8)	7·4 (2·9–16·9)	21·5 (6·9–52·8)	4634·3 (2291·5–8051·2)	21681·7 (10325·5–37819·1)
Measles	0·3 (0·2–0·4)	3·9 (2·9–5·0)	0·0 (0·0–0·1)	0·4 (0·3–0·5)	0·0 (0·0–0·0)	0·1 (0·1–0·2)	65·4 (21·2–147·7)	544·0 (176·7–1222·5)	55·7 (18·8–125·8)	499·0 (169·5–1133·0)	32·2 (12·1–68·2)	414·6 (155·2–874·6)	680·3 (245·8–1480·6)	3751·2 (1318·2–8261·1)
*Morganella* spp	2·0 (1·3–3·1)	0·3 (0·1–0·6)	1·3 (0·9–1·8)	0·1 (0·0–0·2)	2·0 (1·4–2·8)	0·9 (0·5–1·6)	0·8 (0·4–1·4)	0·7 (0·2–1·7)	2·1 (1·3–3·1)	1·4 (0·7–2·5)	1·1 (0·6–1·9)	0·3 (0·1–0·7)	0·8 (0·4–1·2)	0·8 (0·4–1·5)
*Mycoplasma* spp	34·9 (29·8–41·4)	172·9 (138·4–217·0)	14·2 (11·4–18·0)	15·3 (10·7–21·7)	35·5 (28·3–44·3)	191·5 (138·5–257·6)	45·5 (34·9–58·3)	314·5 (225·8–422·9)	77·9 (59·9–99·7)	642·7 (458·0–859·1)	25·7 (20·1–33·0)	167·7 (129·0–213·6)	204·6 (156·2–262·7)	1123·2 (833·8–1492·3)
*Neisseria gonorrhoeae*	2·8 (2·4–3·3)	0·0 (0·0–0·0)	1·1 (1·0–1·3)	0·0 (0·0–0·0)	2·9 (2·5–3·3)	0·0 (0·0–0·0)	1·3 (0·9–1·7)	0·0 (0·0–0·0)	5·4 (3·9–6·8)	0·0 (0·0–0·0)	1·5 (1·1–1·8)	0·0 (0·0–0·0)	4·9 (3·8–6·1)	0·0 (0·0–0·0)
*Neisseria meningitidis*	42·6 (24·5–68·4)	161·1 (103·2–247·1)	11·0 (6·2–17·4)	41·1 (25·5–61·2)	68·2 (43·4–100·5)	490·7 (323·2–709·9)	86·8 (52·0–134·9)	562·8 (347·5–873·0)	144·4 (96·2–214·8)	934·2 (625·1–1380·7)	46·4 (29·7–70·2)	316·4 (217·5–447·7)	422·7 (300·3–594·2)	2078·1 (1423·2–2994·8)
Norovirus	35·1 (5·9–70·4)	72·2 (20·1–151·9)	18·5 (2·8–36·2)	29·2 (7·5–59·2)	54·1 (13·5–104·0)	260·9 (78·4–512·7)	52·3 (13·4–116·6)	338·1 (90·8–755·7)	90·1 (18·1–204·5)	342·7 (97·9–737·3)	32·4 (5·7–77·9)	107·8 (30·9–228·7)	301·4 (70·6–627·9)	1213·3 (316·8–2556·3)
Onchocerciasis	0·0 (0·0–0·0)	0·0 (0·0–0·0)	0·0 (0·0–0·0)	0·0 (0·0–0·0)	0·1 (0·0–0·2)	0·0 (0·0–0·0)	0·7 (0·5–1·1)	0·0 (0·0–0·0)	0·0 (0·0–0·0)	0·0 (0·0–0·0)	0·0 (0·0–0·0)	0·0 (0·0–0·0)	113·7 (70·6–168·3)	0·0 (0·0–0·0)
Other *Enterococcus* spp	36·4 (21·4–58·5)	45·7 (26·6–74·3)	25·6 (17·3–36·8)	19·0 (11·2–30·2)	36·5 (25·5–51·6)	129·0 (83·4–194·0)	26·0 (15·3–41·3)	108·6 (61·5–175·7)	45·7 (29·9–68·9)	232·0 (135·8–371·7)	30·0 (18·3–44·4)	73·3 (46·7–109·1)	61·1 (39·2–93·5)	305·5 (185·6–480·4)
Other *Klebsiella* spp	29·8 (15·8–50·7)	4·9 (1·9–10·4)	15·1 (8·7–24·8)	2·9 (1·0–7·0)	23·1 (12·3–39·1)	13·4 (5·2–28·5)	14·4 (7·3–25·5)	10·3 (3·8–23·2)	23·5 (10·8–43·3)	8·0 (2·9–17·5)	15·5 (7·8–27·2)	9·2 (3·7–19·1)	26·3 (11·6–48·6)	27·5 (10·2–56·2)
Other neglected tropical diseases	13·0 (8·9–18·5)	46·2 (31·4–65·3)	4·7 (3·2–6·7)	15·7 (10·5–23·0)	20·5 (14·9–28·1)	86·8 (62·2–119·4)	18·9 (12·7–27·5)	72·0 (48·1–103·9)	60·6 (42·6–82·8)	137·1 (95·0–191·7)	9·9 (6·8–14·0)	31·7 (21·8–44·4)	131·3 (81·4–315·9)	516·3 (295·1–1483·5)
Other unspecified infectious diseases	26·9 (17·1–35·9)	170·1 (62·1–268·5)	29·5 (23·3–38·5)	72·7 (55·5–95·3)	40·6 (32·1–53·2)	129·1 (94·5–184·8)	49·5 (38·9–67·4)	155·7 (96·4–310·9)	108·5 (74·0–141·2)	344·1 (193·3–517·9)	34·6 (25·0–41·6)	125·2 (79·9–160·2)	130·4 (92·3–165·6)	502·0 (337·3–669·4)
Polymicrobial infections	52·3 (31·8–80·9)	294·2 (182·5–452·8)	39·5 (22·3–64·0)	107·7 (61·7–172·1)	100·3 (65·3–145·7)	772·9 (491·1–1122·3)	112·6 (68·9–170·9)	795·0 (471·8–1205·4)	216·6 (137·1–323·4)	1918·1 (1175·8–2870·8)	117·7 (71·7–182·8)	585·4 (379·6–853·8)	326·5 (224·4–468·5)	1956·3 (1327·4–2835·4)
*Proteus* spp	45·3 (29·0–65·7)	19·7 (12·5–29·9)	29·0 (20·1–40·5)	8·1 (5·0–12·6)	38·9 (26·8–54·5)	54·9 (37·1–78·8)	23·6 (14·1–36·6)	44·8 (26·5–69·3)	40·5 (26·2–59·1)	82·6 (52·5–129·9)	25·5 (16·1–37·7)	26·2 (17·1–37·9)	47·9 (31·1–71·3)	141·0 (92·5–207·2)
*Providencia* spp	1·5 (0·9–2·4)	0·3 (0·1–0·7)	0·6 (0·4–0·9)	0·1 (0·0–0·2)	1·8 (1·2–2·6)	0·9 (0·4–1·9)	0·9 (0·4–1·6)	1·3 (0·3–3·4)	3·0 (2·0–4·6)	2·1 (0·9–4·2)	1·1 (0·6–1·8)	0·4 (0·1–1·0)	1·3 (0·7–2·1)	1·4 (0·6–2·8)
*Pseudomonas aeruginosa*	216·0 (144·9–309·8)	426·0 (326·8–561·9)	163·1 (114·1–224·6)	100·1 (63·5–149·2)	221·4 (151·9–310·7)	751·3 (515·0–1045·9)	180·9 (119·0–260·1)	831·1 (562·8–1149·5)	262·3 (182·9–361·0)	1592·1 (1132·9–2144·6)	157·4 (104·7–227·3)	476·5 (345·5–627·8)	472·6 (351·1–621·5)	2347·9 (1763·1–3073·0)
Rabies	0·8 (0·6–1·2)	0·3 (0·2–0·7)	0·0 (0·0–0·0)	0·1 (0·1–0·1)	0·1 (0·1–0·2)	0·2 (0·0–0·3)	0·7 (0·2–1·0)	0·9 (0·2–2·2)	22·4 (11·0–32·9)	26·8 (13·2–47·4)	5·4 (1·7–7·7)	6·4 (1·5–11·5)	34·0 (11·2–60·6)	74·2 (22·0–141·5)
Respiratory syncytial virus	68·6 (56·0–84·4)	922·2 (738·3–1159·9)	12·6 (10·5–15·6)	112·2 (75·8–165·4)	87·7 (62·8–117·4)	990·2 (692·4–1344·8)	134·7 (97·5–177·0)	1333·7 (956·2–1760·5)	241·3 (181·2–310·6)	2605·0 (1944·6–3363·4)	56·7 (44·1–70·6)	803·4 (621·2–1008·6)	569·0 (439·0–733·6)	3663·4 (2820·8–4730·9)
Rotavirus	21·3 (9·0–40·0)	115·5 (51·0–206·4)	8·7 (3·5–17·1)	37·5 (15·4–71·0)	73·5 (36·3–131·0)	412·4 (193·5–729·5)	155·7 (75·1–278·3)	1141·2 (506·3–2071·3)	165·1 (77·3–319·6)	527·1 (229·6–950·5)	61·2 (30·5–111·4)	515·7 (268·2–813·9)	791·0 (391·8–1315·4)	4710·5 (2312·5–7814·7)
*Salmonella* Paratyphi	0·0 (0·0–0·1)	0·0 (0·0–0·0)	0·0 (0·0–0·0)	0·0 (0·0–0·0)	0·0 (0·0–0·1)	0·1 (0·1–0·3)	0·3 (0·1–0·6)	0·3 (0·1–0·9)	84·1 (34·7–162·9)	128·2 (39·6–306·3)	4·2 (1·7–8·6)	10·0 (2·9–24·7)	2·6 (1·0–5·6)	6·0 (1·9–14·4)
*Salmonella* Typhi	9·4 (4·5–18·1)	35·1 (19·9–57·5)	1·6 (0·8–2·8)	5·1 (2·9–8·3)	21·2 (13·7–31·6)	151·6 (96·0–224·7)	59·2 (36·8–89·5)	315·4 (191·0–475·2)	378·9 (211·7–604·3)	1202·0 (695·9–1848·9)	51·4 (28·6–84·6)	176·9 (105·1–281·2)	440·6 (299·0–634·1)	1949·6 (1276·6–2854·8)
Schistosomiasis	0·0 (0·0–0·0)	0·0 (0·0–0·0)	0·0 (0·0–0·0)	0·0 (0·0–0·0)	16·5 (9·8–28·1)	0·7 (0·2–1·6)	17·6 (10·3–30·0)	1·6 (0·5–3·7)	0·0 (0·0–0·0)	0·0 (0·0–0·0)	4·6 (2·6–8·2)	0·3 (0·1–0·7)	126·9 (82·9–200·0)	18·4 (11·6–29·1)
*Serratia* spp	26·2 (15·5–40·4)	45·8 (28·1–72·2)	15·1 (9·2–23·0)	15·9 (9·8–24·6)	40·2 (25·7–59·3)	174·2 (114·1–250·7)	36·7 (21·9–57·5)	167·1 (100·2–258·0)	70·2 (43·2–110·4)	402·5 (243·6–630·2)	33·9 (21·0–52·3)	107·8 (71·0–158·7)	119·0 (77·7–176·3)	596·8 (378·9–900·4)
*Shigella* spp	9·0 (3·6–18·3)	54·7 (18·5–117·0)	2·1 (0·9–4·5)	5·8 (2·0–13·5)	26·2 (11·4–48·8)	216·3 (88·0–412·7)	38·1 (14·1–83·5)	291·9 (93·7–669·2)	83·9 (33·1–169·2)	501·6 (185·9–1000·9)	11·7 (4·9–23·1)	108·8 (40·1–219·5)	487·7 (215·1–857·5)	2604·6 (1112·0–4757·3)
*Staphylococcus aureus*	454·4 (316·4–633·9)	709·6 (558·7–903·7)	430·2 (307·7–590·6)	188·4 (122·2–275·3)	434·1 (320·4–577·2)	1138·1 (801·0–1563·0)	323·8 (226·5–448·5)	1214·8 (874·2–1643·9)	420·0 (313·4–551·1)	2049·9 (1542·5–2663·7)	308·3 (213·0–434·8)	716·8 (547·6–921·1)	854·0 (681·8–1081·1)	3696·1 (2831·6–4813·6)
*Streptococcus pneumoniae*	317·4 (254·9–396·9)	1123·4 (904·1–1411·1)	127·7 (99·8–165·6)	112·6 (78·8–159·9)	275·1 (214·2–351·7)	1306·0 (957·8–1719·2)	330·2 (258·1–424·9)	1971·2 (1515·9–2521·2)	596·3 (483·3–737·4)	4002·6 (3122·2–5099·2)	250·2 (198·7–316·8)	1429·1 (1164·1–1772·9)	1449·0 (1157·2–1814·4)	7388·4 (5688·1–9502·9)
Syphilis	4·5 (2·2–8·3)	53·3 (17·9–108·6)	2·2 (1·5–3·3)	21·0 (8·7–41·7)	41·7 (19·2–79·2)	469·2 (199·6–922·9)	48·1 (15·1–106·2)	476·7 (143·2–1064·0)	89·8 (30·0–193·9)	947·6 (293·6–2086·4)	46·2 (15·4–100·5)	676·2 (208·6–1500·9)	588·4 (209·4–1210·1)	3741·8 (1316·1–7749·3)
Tetanus	0·2 (0·2–0·5)	0·3 (0·2–0·9)	0·1 (0·1–0·3)	0·2 (0·1–0·2)	4·5 (2·8–8·4)	31·7 (14·9–72·8)	16·2 (9·9–26·1)	120·9 (59·0–217·4)	52·0 (35·4–73·1)	449·8 (277·6–667·8)	11·3 (6·8–14·0)	59·5 (43·0–80·0)	120·7 (84·7–196·0)	576·3 (395·3–934·3)
Trachoma	0·0 (0·0–0·0)	0·0 (0·0–0·0)	0·0 (0·0–0·0)	0·0 (0·0–0·0)	0·2 (0·1–0·3)	0·0 (0·0–0·0)	0·8 (0·4–1·3)	0·0 (0·0–0·0)	4·7 (2·8–7·3)	0·0 (0·0–0·0)	0·7 (0·4–1·2)	0·0 (0·0–0·0)	7·0 (4·6–10·0)	0·0 (0·0–0·0)
Trichomoniasis	3·2 (1·2–6·6)	0·0 (0·0–0·0)	2·8 (1·1–5·7)	0·0 (0·0–0·0)	6·6 (2·6–13·5)	0·0 (0·0–0·0)	2·4 (0·9–4·9)	0·0 (0·0–0·0)	2·2 (0·9–4·7)	0·0 (0·0–0·0)	3·5 (1·4–7·3)	0·0 (0·0–0·0)	7·0 (2·7–14·3)	0·0 (0·0–0·0)
Trichuriasis	0·0 (0·0–0·0)	0·0 (0·0–0·0)	0·0 (0·0–0·0)	0·0 (0·0–0·0)	4·0 (2·2–6·6)	2·5 (1·3–4·2)	0·1 (0·0–0·2)	0·1 (0·0–0·1)	3·1 (1·6–5·5)	2·0 (1·1–3·6)	4·3 (2·2–7·1)	3·1 (1·6–5·1)	5·9 (3·2–9·8)	2·7 (1·5–4·3)
Tuberculosis	300·5 (275·2–327·5)	153·5 (123·3–190·1)	31·3 (28·7–33·3)	4·2 (3·7–4·9)	167·4 (147·2–190·4)	120·8 (93·8–152·3)	144·2 (117·5–176·0)	123·3 (82·7–168·7)	1525·8 (1351·1–1724·1)	806·2 (644·6–1002·7)	447·3 (407·3–490·2)	298·4 (243·8–360·5)	2263·6 (1947·7–2638·8)	2613·9 (1974·2–3420·6)
Varicella-zoster virus	4·0 (3·0–5·3)	15·3 (9·4–20·6)	8·2 (6·2–12·3)	8·3 (6·0–15·1)	14·3 (11·0–20·1)	54·2 (39·0–83·0)	12·1 (9·7–15·2)	44·2 (29·1–65·5)	22·1 (18·8–25·7)	80·1 (60·5–104·2)	9·1 (7·5–11·3)	29·4 (23·7–36·9)	52·8 (42·5–64·4)	182·4 (135·3–241·9)
*Vibrio cholerae*	17·8 (9·8–29·8)	46·4 (23·3–88·0)	0·0 (0·0–0·0)	0·0 (0·0–0·0)	19·2 (10·6–33·9)	79·2 (35·1–159·1)	131·6 (64·8–251·2)	605·6 (266·6–1266·3)	56·6 (23·5–119·9)	148·2 (70·4–267·8)	12·5 (5·9–24·4)	73·1 (37·1–133·6)	414·6 (213·4–713·3)	1604·2 (717·0–2911·0)
Viral meningitis	8·4 (5·7–13·5)	15·7 (10·2–24·1)	3·9 (2·6–6·2)	6·5 (4·4–9·1)	9·4 (6·6–13·5)	33·6 (21·4–49·1)	16·0 (9·4–28·2)	58·9 (29·1–119·7)	24·5 (18·7–32·5)	88·8 (62·0–127·9)	7·0 (4·8–11·1)	30·1 (21·3–42·4)	104·4 (72·7–144·1)	357·7 (229·4–529·2)
Visceral leishmaniasis	0·6 (0·0–6·1)	3·1 (0·0–29·8)	0·1 (0·0–0·9)	0·4 (0·0–3·5)	12·3 (0·0–51·5)	36·8 (0·1–146·8)	5·0 (0·0–51·2)	17·0 (0·0–156·7)	5·2 (0·0–31·0)	15·8 (0·0–87·8)	0·0 (0·0–0·0)	0·0 (0·0–0·1)	21·8 (11·5–34·2)	50·7 (25·7–83·1)
Yellow fever	0·0 (0·0–0·0)	0·0 (0·0–0·0)	0·3 (0·0–0·9)	0·4 (0·0–1·9)	0·9 (0·3–2·2)	0·9 (0·2–2·4)	3·9 (0·7–12·1)	3·8 (0·4–15·3)	0·0 (0·0–0·0)	0·0 (0·0–0·0)	0·0 (0·0–0·0)	0·0 (0·0–0·0)	25·0 (9·2–52·1)	24·8 (8·7–54·7)
Zika virus	0·0 (0·0–0·0)	0·0 (0·0–0·0)	0·0 (0·0–0·0)	0·0 (0·0–0·0)	0·1 (0·0–0·1)	0·2 (0·1–0·4)	0·0 (0·0–0·0)	0·0 (0·0–0·0)	0·0 (0·0–0·0)	0·0 (0·0–0·0)	0·0 (0·0–0·0)	0·0 (0·0–0·0)	0·0 (0·0–0·0)	0·0 (0·0–0·0)
**Total**	**5082·2 (4117·3–6298·7)**	**8586·5 (6882·7–10 854·4)**	**2933·4 (2344·2–3696·8)**	**1861·8 (1324·7–2559·4)**	**4968·9 (4067·0–6083·1)**	**16 021·5 (11 861·9–21 090·7)**	**4952·2 (3956·4–6228·7)**	**22 711·3 (17 408·3–28 876·1)**	**9695·6 (8263·7–11 477·7)**	**43 350·7 (33 923·1–54 791·8)**	**4773·9 (3981·9–5792·9)**	**13 627·7 (10 982·6–16 600·2)**	**29 124·9 (25 041·8–34 123·0)**	**116 704·8 (95 281·0–143 065·6)**

95% uncertainty intervals are shown in parentheses. DALY rates are shown to one decimal place. DALYs=disability-adjusted life-years. *E coli*=*Escherichia coli*. GBD=Global Burden of Disease (Study). *Salmonella* Typhi=*Salmonella enterica* serotype Typhi. *Salmonella* Paratyphi=*Salmonella enterica* serotype Paratyphi.

* Excluding enteropathogenic and enterotoxigenic *E coli*.

For all 85 pathogens, the highest age-standardised DALY rates were estimated for sub-Saharan Africa, for all ages (29 124·9 [95% UI 25 041·8–34 123·0] per 100 000 population) and for children younger than 5 years (116 704·8 [95 281·0–143 065·6] per 100 000 population; table 2). Conversely, the lowest age-standardised DALY rates were observed in the high-income super-region, at 2933·4 (2344·2–3696·8) per 100 000 population (all ages), and 1861·8 (1324·7–2559·4) per 100 000 population (under 5s).

**Figure 4:**
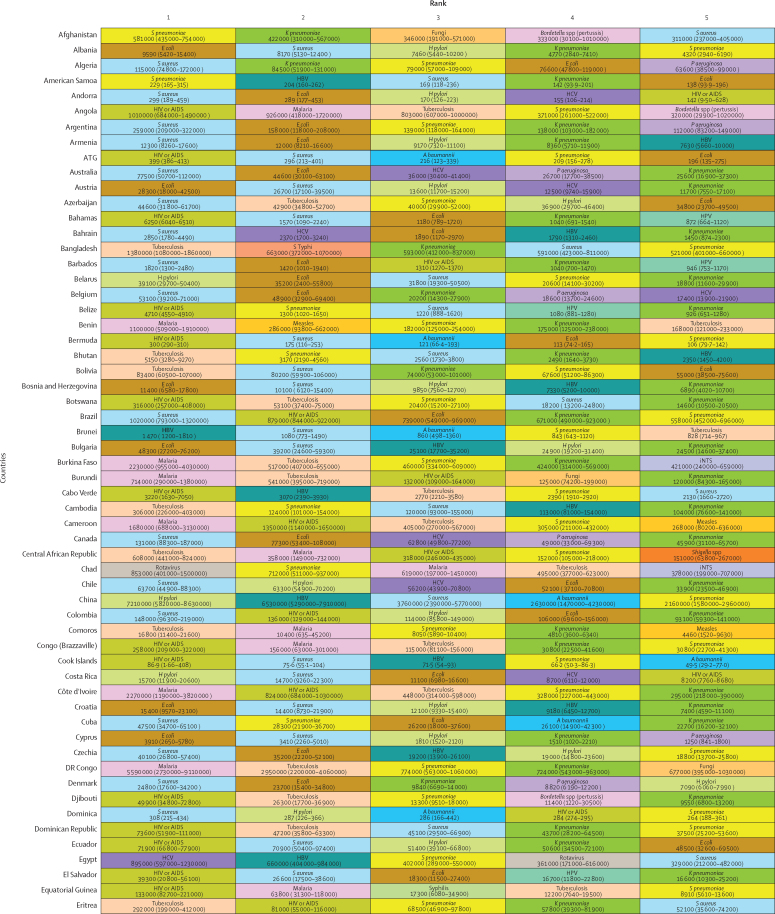

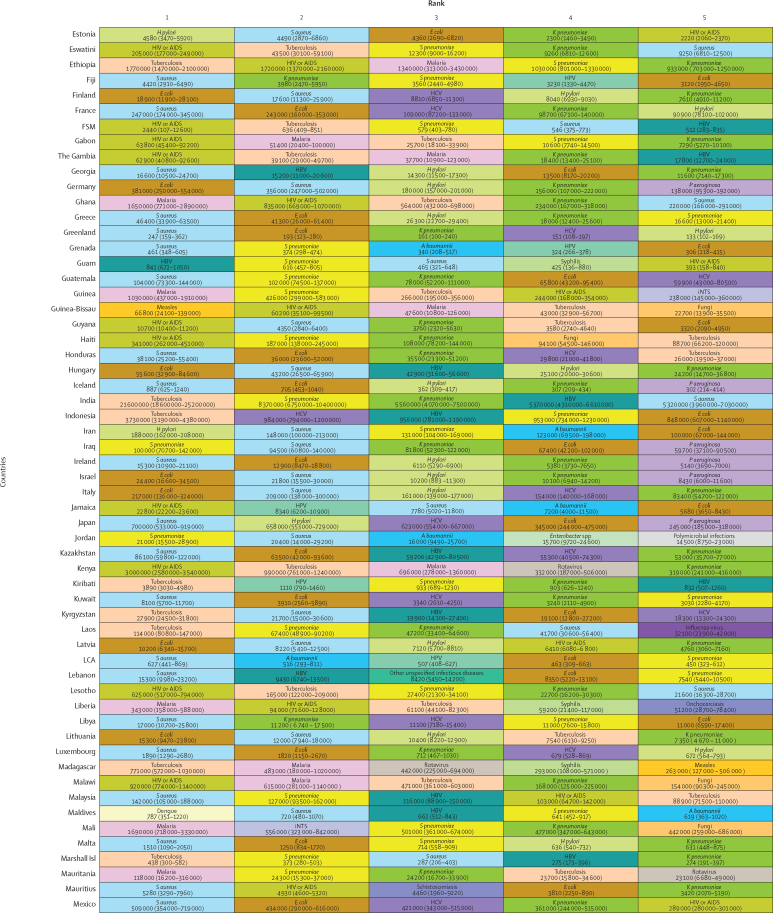

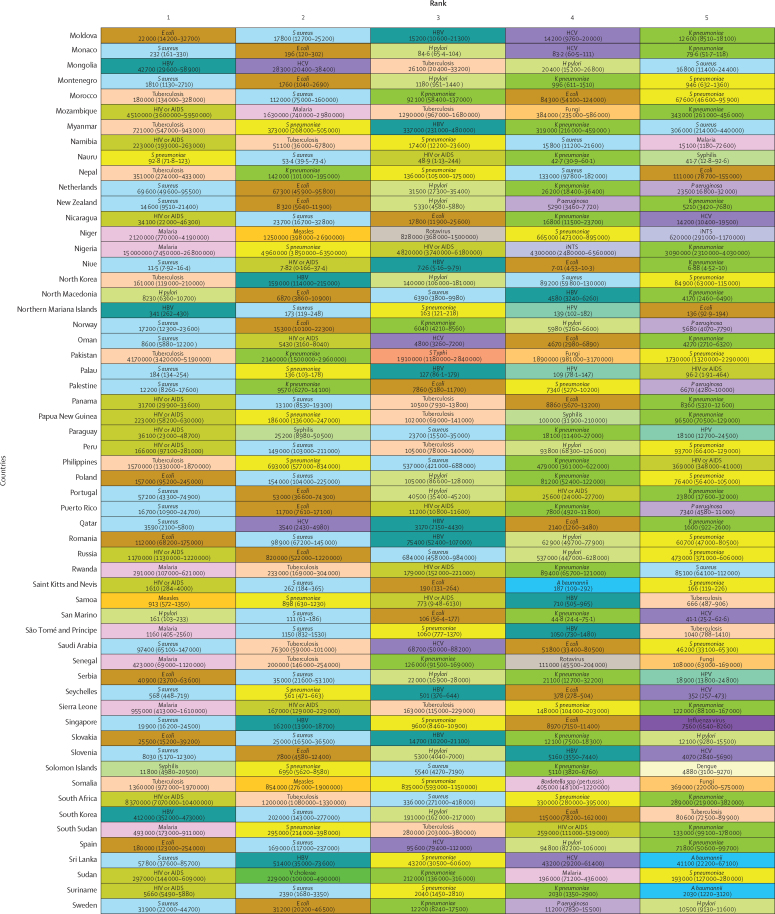

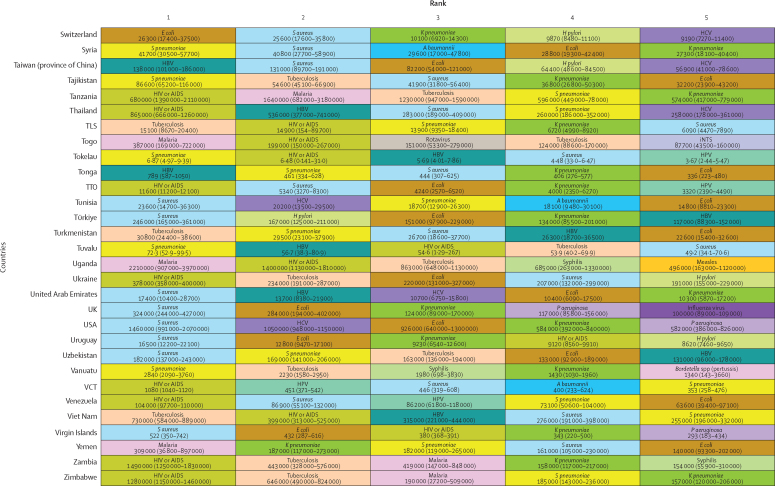
DALYs associated with the top five pathogens in each of 204 countries and territories in 2019 Colours represent pathogens. Country order is alphabetical. DALYs are shown as counts, presented to three significant figures. 95% uncertainty intervals are shown in parentheses. *A baumannii*=*Acinetobacter baumannii*. ATG=Antigua and Barbuda. DALYs=disability-adjusted life-years. *E coli*=*Escherichia coli* (excluding enteropathogenic and enterotoxigenic *E coli*). FSM=Federated States of Micronesia. HBV=hepatitis B virus. HCV=hepatitis C virus. *H pylori*=*Helicobacter pylori*. HPV=human papillomavirus. iNTS=invasive non-typhoidal *Salmonella*. Isl=Islands. *K pneumoniae*=*Klebsiella pneumoniae*. LCA=Saint Lucia. *P aeruginosa=Pseudomonas aeruginosa*. *S aureus*=*Staphylococcus aureus*. *S pneumoniae*=*Streptococcus pneumoniae*. *S* Typhi=*Salmonella enterica* serotype Typhi. TLS=Timor-Leste. TTO=Trinidad and Tobago. VCT=Saint Vincent and the Grenadines.

*S aureus* was a leading pathogen according to DALY burden in 64 (31·4%) of the 204 countries and territories, followed by HIV or AIDS in 44 (21·6%) countries, tuberculosis in 25 (12·3%), malaria in 21 (10·3%), *E coli* in 20 (9·8%), *S pneumoniae* in ten (4·9%), and *H pylori* in seven (3·4%; figure 4). We observed notable differences between countries in the burden of *H pylori*. Estimates of death rates for all ages and children younger than 5 years according to GBD super-region are presented in the appendix (pp 31–35).

## Discussion

To our knowledge, this study presents the most comprehensive estimation of the health burden associated with specific pathogens to date, in terms of the number of pathogens and pathogen groups assessed, and inclusivity, incorporating pathogens involved in the pathway to death. We found that in 2019, the 85 pathogens analysed (which included specific causative agents, pathogen groups, infectious conditions, and aggregate categories) were collectively associated with more than 700 million DALYs, which was a substantial proportion of the overall burden from all diseases. The pathogen-associated burden comprised 27·7% of the total DALY burden in all ages, and 65·5% of the total DALY burden in children younger than 5 years. We noted considerable differences among super-regions and countries with respect to both the total burden from these 85 pathogens and the burden of specific pathogens nationally in terms of those ranked top five for each country.

As the leading three pathogens or infectious entities, tuberculosis, malaria, and HIV or AIDS warrant the considerable attention they receive from the global health community.^16^, ^17^ However, it is important to recognise that numerous other pathogens impose a substantial burden and perhaps deserve increased consideration globally. First, infections associated with Gram-negative bacteria, such as *K pneumoniae, E coli, P aeruginosa,* and *A baumannii*, were estimated to account for a large associated burden of disease (ie, 114 million DALYs collectively on a global level). Such a burden poses a substantial threat in health-care settings, leading to increased treatment costs, prolonged hospital stays, and elevated mortality rates, with the additional compounding effect of outbreaks in hospital settings that further contribute to the disease burden.^18^ Furthermore, these infections often occur in immunocompromised individuals, hospitalised patients, older individuals, or people with chronic illnesses, and are often caused by pathogenic species carrying various resistance genes.^18^, ^19^ Therefore, addressing the burden of Gram-negative infections requires a multifaceted approach and implementable policy changes informed by precise estimates, as well as increased vaccine development efforts, considering that currently no vaccines are available for the aforementioned pathogens.^18^, ^19^

Second, *S aureus* was the leading cause of associated DALY burden in nearly a third of the countries in our analysis, despite ranking fifth in the overall global DALY burden list. This bacterial species was previously shown to be associated with more than one million deaths in 2019 and had the highest mortality burden among 33 bacteria in 135 countries.^5^ A recent systematic review and meta-analysis found that between 3–6% of pneumonia cases in children younger than 5 years hospitalised for all-cause pneumonia are caused by *S aureus*, contributing to the estimated burden in this age group.^20^ In our study, *S aureus* was associated with the sixth highest DALY burden in children younger than 5 years. This pathogen has also emerged as one of the main drivers of antimicrobial resistance burden on a global level and in the WHO European region.^9^, ^21^ Despite the substantial burden imposed by severe and often drug-resistant *S aureus* infections, the absence of an effective vaccine remains an important challenge as previous vaccine candidates have failed to generate any lasting immune response.^22^ Nonetheless, findings from clinical and preclinical research studies provide valuable insights, highlighting specific targets that hold promise and could serve as potential avenues for vaccine developers to explore in the future.^22^ Despite the introduction of new antibiotics to target antibiotic-resistant isolates of *S aureus*, reductions in mortality due to this pathogen below 20% are yet to be achieved;^23^ therefore, a viable and efficacious vaccine could be impactful in both high-income and low-income countries.

Third, *H pylori*, which is a causative agent for gastric cancer, ranked as the leading cause of DALY burden in seven countries, and a second and third leading cause in an additional four and 28 countries, respectively. Geographical differences linked to gastric cancer burden associated with *H pylori* have been described previously.^24^ Considering *H pylori's* high prevalence in the general population in many countries, it is not surprising that many recent studies are again emphasising the importance of eradication efforts in countries with a high burden of gastric cancer,^24^, ^25^ with antibiotic resistance surveillance and vaccine development efforts becoming increasingly recognised as crucial additional steps.^26^, ^27^ The World Gastroenterology Organisation has highlighted the pivotal role of local factors in determining the impact and management strategies for *H pylori* infection, which is in accordance with differences in demonstrated burden among countries.^28^ Specifically, to ensure the most effective approaches, it is essential to use country-specific burden estimates and the best available local knowledge, rather than simply extrapolating from guidelines formulated in other regions.

For viruses, our analysis showed substantial DALY burdens associated with hepatitis B and C, which ranked respectively as the eighth and 14th highest among all pathogens globally. The under-recognition of hepatitis B and C burden arises from various factors, including inadequate screening and testing programmes, limited awareness among health-care providers and the general population, and unabating social stigmatisation.^29^, ^30^ Thus, recognising the burden of hepatitis B and C is essential for developing comprehensive public health strategies that prioritise prevention, early detection, and access to appropriate care. Efforts to raise awareness, improve screening and testing initiatives, and expand treatment access will be pivotal in mitigating the global burden of hepatitis B and C and reducing associated morbidity and mortality.^30^, ^31^

Influenza virus was the seventh highest ranked pathogen for DALYs in children younger than 5 years, and the 11th highest for all age groups. Influenza virus is notable for its economic impact in low-income and middle-income countries (LMICs), from the direct costs to health-care services and households, to indirect costs and broader adverse effects on economies (despite the seasonal and yearly variations inherent in influenza patterns).^32^ This economic burden is not limited to LMICs alone: a recent study examining the costs of paediatric influenza on the health-care system and society in Europe also revealed substantial direct and indirect costs.^33^ As new and relevant studies continue to show that receiving influenza vaccines considerably lowers the incidence rate of severe disease requiring intensive care,^34^, ^35^ the broader impact of vaccinating children younger than 5 years is pivotal to consider, not only to reduce the burden of disease on health care but also to reduce its associated economic burden, especially in LMICs.

We estimated rotavirus to be associated with more than 10 million DALYs among children younger than 5 years, ranking ninth for disease burden in this age group. This estimate corroborates findings from the Global Pediatric Diarrhea Surveillance Network that, despite the vital impact from the rotavirus vaccine, rotavirus continues to be the primary cause of hospital admissions for paediatric diarrhoea in all regions except the Americas.^36^ Nonetheless, at sites where the rotavirus vaccine had been introduced, the proportion of hospitalisations attributed to rotavirus was approximately 50% lower than at sites where the vaccine had not yet been implemented,^36^ suggesting real-world effectiveness of the vaccine. However, the ongoing burden of disease highlights the need to increase vaccine delivery and uptake in high-burden regions and to identify other preventable factors contributing to viral transmission.

Among the pathogens that receive considerable global attention, HPV had a burden that was smaller than anticipated. This finding might reflect past successes in various public health programmes and interventions. We should emphasise that in this study we have focused on cervical cancer as a proxy of the burden associated with HPV infection; although cervical cancer has been extensively studied as the primary outcome associated with HPV infection, we are cognisant that other forms of HPV-related cancers can be overlooked in terms of research investment.^37^ Nevertheless, our study should not detract from hard-won gains in HPV immunisation, particularly in sub-Saharan Africa, where the aggressive nature of the disease is intertwined with the HIV burden.^38^ The results of our study simply caution that there are hitherto neglected priorities in terms of the pathogen-associated burden.

We found considerable disparity in the proportion of total DALYs from all causes that were associated with infectious causes among different super-regions, especially comparing sub-Saharan Africa with the high-income region. The super-region of sub-Saharan Africa had the highest percentage of infectious DALYs among total DALYs, at 61·5% in all ages (79·3% in those younger than 5 years), whereas the high-income region had the lowest percentage, at 9·8% in all ages (16·5% in those younger than 5 years). This discrepancy can be attributed to several factors, including poor sanitation, limited access to clean water, and poor hygiene practices in LMICs,^39^ and notable differences in health-care infrastructure, access to essential medicines, and prevention options between LMICs and high-income settings.^40^, ^41^ In our study, the fraction of DALYs associated with infectious causes was also high in south Asia, a finding consistent with literature describing the sizeable economic consequences of communicable diseases such as HIV or AIDS, malaria, and dengue fever on individuals in resource-poor settings.^42^ These findings highlight the need for both region-specific and global estimates for improved priority setting and policy development.

A study by Head and colleagues found that, from 2000 to 2017, global spending on infectious disease research was US$105 billion (with 74·8% of this funding in preclinical science and 20·4% in public health);^43^ however, the allocation of this funding did not necessarily correspond to the burden of disease or the level of risk posed by specific infections. For instance, the study ranked genital herpes among the top two positions in terms of investment, with $3101 per DALY; this is incongruent with our burden assessment in which it was responsible for 0·253 million (95% UI 0·080–0·628) DALYs (with a DALY rate of 3·3 [95% UI 1·1–8·1] per 100 000 population). Furthermore, the previous study, which used GBD data, reported that syphilis received the lowest proportion of investment, with only $9 per DALY.^43^ However, our research indicates that syphilis was responsible for 9·54 million (3·00–19·4) DALYs in 2019 (with a DALY rate of 123·3 [43·9–250·4] per 100 000 population), primarily affecting children younger than 5 years, which is consistent with reports from some countries of surges in congenital syphilis rates.^44^, ^45^, ^46^ Similarly, *S aureus* and Gram-negative bacterial infections (primarily *E coli* and *Pseudomonas* spp) received a relatively low investment in research and development,^43^ when considering the associated DALY burden estimated in our study. We acknowledge that our estimates are a single epoch of burden and successful research programmes might have led to reduced incidence and mortality, creating the appearance of a misalignment of funding and burden. Nevertheless, by aligning research funding with the burden of pathogens, we can make substantial progress in preventing and treating such infections.

We should be cognisant that funding decisions are also hampered by insufficient diagnostic capabilities and the associated funding constraints of some countries. As shown by Tufa and colleagues,^47^ many African nations exhibit either non-existent or severely restricted blood culture infrastructure; and the financial responsibility for blood culture procedures typically burdens the patient and their family. In addition, a quantitative estimate of current disease burden alone should not be the deciding factor regarding whether a drug or vaccine should be developed for a specific infectious cause. However, investments in high-efficacy vaccines to help eliminate specific diseases could have substantial long-term cost and health benefits,^48^ even if the current burden is low. The need for novel antimicrobials should also be considered for pathogens that are not currently presenting the issue of resistance but might in the future.

Notably, of the 85 pathogens presented in this study, vaccines are currently available for just 22, or a quarter. These 22 pathogens are tuberculosis, malaria, *Streptococcus pneumoniae*, hepatitis A, B, and E virus, influenza, rotavirus, *Salmonella enterica* serovar Typhi, *Bordetella* spp, *Neisseria meningitidis*, human papillomavirus, measles, cholera, *Haemophilus influenzae*, dengue virus, tetanus, varicella-zoster virus, rabies, diphtheria, yellow fever, and ebola virus. These 22 pathogens accounted for 302 million DALYs in 2019, meaning that more than 400 million DALYs are due to infectious causes for which vaccines are not available or are in the pipeline.^26^, ^49^ In addition, among the top ten leading infectious causes in terms of DALY burden, vaccines are currently only available for three (tuberculosis, malaria, and *S pneumoniae*), and these vaccines have low effectiveness.^50^, ^51^, ^52^ Studies have also shown that globally, 20 million infants younger than 1 year are not receiving their complete series of recommended vaccinations. Most of these children belong to the lowest socioeconomic groups and are at the highest risk of disease.^53^, ^54^ Additionally, gains in global childhood vaccine coverage have stalled or even reversed from 2010 to 2019,^55^ which was especially evident for diphtheria–pertussis–tetanus vaccine coverage in Africa^56^ and routine measles vaccination in LMICs.^57^ Therefore, all countries should be urged to close identified equity gaps by improving stagnating vaccine coverage. We also recognise the crucial need for improved treatments and evidence-based guidelines in managing sepsis caused by major pathogens. In particular, focus should be on coordinating clinical trials to register new agents, conducting comparative effectiveness trials for existing treatments, and promoting implementation science in LMICs (in relation to interventions such as hospital infection control and antimicrobial stewardship).

Prior to the current analysis, three extensive multinational studies used the DALY metric to evaluate the impact of infectious diseases: GBD 2019,^1^ the Burden of Communicable Diseases in Europe project,^58^ and WHO estimates of the global burden of food-borne diseases.^59^ These studies used diverse methodological approaches to estimate DALYs. A crucial methodological decision regarding YLD calculations involves choosing between a prevalence-based approach^58^, ^59^ or incidence-based approach;^1^ for example, our study adopted a prevalence-based approach in accordance with GBD methods. In addition to these multinational endeavours, manifold independent studies on the burden of communicable diseases, in which researchers conducted their own YLL, YLD, or DALY calculations using primary epidemiological sources, have been independently done, with many originating from Europe.^60^ The most extensively researched infectious diseases are food-borne and water-borne illnesses, with the Netherlands having the highest number of such publications (with substantial variations in terms of scope and applied methodologies).^60^

Our estimation approach used the measure of associated with burden, which, to our knowledge, is the most inclusive approach used to date, as it considers all scenarios in which a pathogen is involved in the pathway to death, even if the cause of death is not directly attributed to the pathogen itself. This measure captures the full impact of pathogens on mortality and disability, providing a more accurate assessment of their burden than the underlying cause approach or attributable cause approach. However, alongside this pathogen-associated burden, in the future it will be pivotal to calculate the attributable burden to quantify the direct effect of a specific pathogen on mortality or morbidity. Knowing the attributable burden could also potentially enable the calculation of DALYs averted as a measure of the reduction in disease burden attributed to an intervention, measuring its effect on population health. Furthermore, calculation of attributable burden might represent the most salient approach to inform decisions on vaccine research and prioritisation.

Our study includes several limitations, many of which are linked to already recognised data sparsity issues.^61^ The input data for modelling has incomplete geographical coverage and varies in quality for many LMICs, highlighting the need for capacity building in those areas. Different countries might have varying capacities and systems for detection and reporting of communicable diseases, which can subsequently bias estimates and, in turn, make cross-pathogen and country comparisons challenging. Furthermore, the pathway to death framework relies on clinician adjudication for establishing whether the disease of interest had a causative role in an individual's death, introducing the potential for misclassification bias inherent in subjective clinical judgment rather than objective laboratory metrics. Misclassification bias is also pertinent for morbidity estimates, as in some instances testing is not sufficient to allow for pathogen identification, or to distinguish between colonisation and infection. Furthermore, our estimates for HPV and *H pylori* have been quantified with use of cervical and non-cardia gastric cancers as proxies, which omits the low but increasingly recognised burdens of other malignancies associated with these two infectious agents.^62^, ^63^ The decision to group fungi together was influenced by the diagnostic complexities associated with detecting fungal pathogens, and we are cognisant that diagnostic yield when culture methods are used can be suboptimal (not only for fungi, but for some bacterial species such as *Mycoplasma* spp). Cases of tuberculosis-associated or HIV-associated opportunistic infections were not included as part of the causative pathway for these diseases, given that they are categorised as cases of tuberculosis and HIV according to GBD methodology. We are aware that some comorbidities at older ages might aggravate the severity of a given infectious disease, implying the need to modify disability weights and consider the attributable fraction due to the infections as opposed to the other underlying conditions. Our YLD estimates for many of our infections are only for acute infection, so they do not include other post-acute sequelae of the disease (eg, post-traumatic stress disorder after intensive care unit admission due to sepsis, rheumatic fever after *S pyogenes* infection, childhood stunting after *Shigella* infection, post-kala-azar dermal leishmaniasis, or post-Ebola syndrome), leading to under-representation of the DALY burden of implicated pathogens. We acknowledge potential biases, such as selection bias linked to the use of passive microbial surveillance data; however, we addressed specific biases for included pathogens (eg, by using spatial information to adjust the bias associated with the location of antenatal care clinics when estimating HIV burden; appendix pp 16–22).^1^ The exact drivers of high burden of fungal infections must still be elucidated to put the results into context. Some other categories in our study should include more detail (such as the polymicrobial category), which we plan to addresss in future research endeavours. Future studies should also account for the complex interactions and cumulative burden of multiple diseases over a lifecourse.

In conclusion, we estimated the fatal and non-fatal burden, expressed as DALYs, associated with 85 pathogens globally in 2019. We included pathogens when they were observed as intermediate causes of death and disability to accurately evaluate and compare impacts on population health. In this comprehensive analysis, we estimated that more than 700 million DALYs were associated with 85 pathogens, and that this burden disproportionately affected children younger than 5 years. We identified pathogens with sizeable associated burden of disease that have not been frequently considered in priority setting exercises and policy-level discussions. Therefore, we urgently call for further research in drug development, vaccinology, and pathogen biology to innovate and accelerate drug and vaccine development for the broader group of pathogens highlighted in these rankings.

## Data sharing

To download the data used in these analyses, please visit the Global Health Data Exchange at https://ghdx.healthdata.org/record/ihme-data/global-burden-85-pathogens-2019

## Declaration of interests

B S Cooper reports support to their institution (University of Oxford) for their contribution to this research from the Wellcome Trust, and from the Department of Health and Social Care using UK aid funding managed by the Fleming Fund. E Chung reports support from the US National Institutes of Health (training grant NICHD T32HD007233). S J Dunachie has received support from the UK Fleming Fund at the Department of Health and Social Care, the Bill & Melinda Gates Foundation, Wellcome Trust, the UK National Institute of Health and Care Research, and a Wellcome Drug Resistant Infections Discretionary Award; served as Scientific Advisor for the Scottish Parliament, for which a fee was received; served on funding committees for Wellcome Trust, for which fees were received; served as a Data Monitoring Committee member for the UK STABILISE study of BCG vaccine in COPD; is a member of the New and Emerging Respiratory Virus Threats Advisory Group; is Chair of the Wellcome SEDRIC subgroup on Data Standards and Harmonisation in Antimicrobial Resistance; is a member of the Variant Technical Group for SARS-CoV-2 (invited as a T-cell specialist) for the UK Health Security Agency; is an expert advisor to WHO's Global Antimicrobial Resistance Surveillance System; and is a member of the WHO Guidelines Development Group on Treatment of Ebola. C E Moore reports participation on an advisory board for an MRC grant (no payments received); participation in a WHO Advisory Group for the WHO Medically Important Antimicrobial List; participation in a REVIVE Advisory Group as a member of the Steering Group for the REVIVE study; and served as an unpaid Co-Chair of the Impact and Influence Group for the Microbiology Society. J F Mosser reports grant funding from the Bill & Melinda Gates Foundation and GAVI, and travel support for attending meetings from the Bill & Melinda Gates Foundation. A Stergachis reports being a member of the Executive Board for the Safety Platform for Emergency Vaccines, Brighton Collaboration, based at the Task Force for Global Health; Chair of the Data Safety Monitoring Board for the IMPROVE 1 and 2 trials in Malawi, Tanzania, and Kenya; member of the Data Safety Monitoring Board for the Improving Neonatal Health Through Rapid Malaria Testing in Early Pregnancy with High-Sensitivity Diagnostics study; and member of the Scientific Advisory Board for Vivli AMR Register.
