# Supplementary material for: Global burden associated with 85 pathogens in 2019: a systematic analysis for the Global Burden of Disease Study 2019
Source: Lancet Infect Dis. 2024 Aug;24(8):868–95. doi: 10.1016/S1473-3099(24)00158-0 (PMC11269650; doi:10.1016/S1473-3099(24)00158-0)
Supplement: Supplementary appendix [file mmc1.pdf]

THE LANCET
Infectious Diseases

Supplementary appendix

This appendix formed part of the original submission and has been peer reviewed. We post it as supplied by the authors.

Supplement to: IHME Pathogen Core Group. Global burden associated with 85 pathogens in 2019: a systematic analysis for the Global Burden of Disease Study 2019. *Lancet Infect Dis* 2024; published online April 16. https://doi.org/10.1016/S1473-3099(24)00158-0.

Appendix: supplementary methods and results to "Global burden associated with 85 pathogens in 2019: a systematic analysis for the Global Burden of Disease Study 2019"

This appendix provides further methodological details and supplementary figures/tables for "*Global burden associated with 85 pathogens in 2019: a systematic analysis for the Global Burden of Disease Study 2019*". Parts of the appendix are taken directly from the appendix of the papers "*Global burden of bacterial antimicrobial resistance in 2019: a systematic analysis*"[1] and "*The burden of antimicrobial resistance in the Americas in 2019: a cross-country systematic analysis*"[2] which are referenced throughout the text.

Table of Contents

Section 1: List of Abbreviations 3

Section 2: Data sources[1,2] 3

Section 2.1: Multiple causes of death and vital registration (MCoD-VR) data. 3

Section 2.2: Hospital discharge data. 4

Section 2.3: Linkage data sources. 4

Section 2.4: Mortality surveillance in the Child Health and Mortality Prevention Surveillance (CHAMPS) study. 4

Section 2.5: Literature review of the microbial aetiology of meningitis, maternal and neonatal sepsis, lower respiratory infections, urinary tract infections, skin infections, peritonitis, and bone and joint infections 4

Section 2.6: Exclusion criteria for literature reviews 6

Section 2.7: Laboratory-based passive surveillance data. 6

Section 3: Supplementary methods: a summary of the estimation process[1,2] 7

Section 3.1: GBD 2019 framework 7

Section 3.2: Deaths where infection plays a role and infectious syndrome estimation 7

Section 3.3: Case fatality ratios 15

Section 3.4: Pathogen distribution 18

Section 4: Pathogen-specific ratios for GBD pathogen adjustment 25

Section 4.1: Generating the ratios 26

Section 4.2: Exceptions 28

Section 4.3: Ratios 28

Section 5: *Helicobacter pylori* burden estimation: 30

Section 6: Supplementary tables 31

Section 7: Estimation Flowchart 54

Section 8: GATHER Compliance: Guidelines for Accurate and Transparent Health Estimates Reporting 55

Section 9: References 57

Section 10: Authors' Contributions 58

Section 1: List of Abbreviations

Abbreviation	Full phrase
DALYs	disability-adjusted life-years
GBD	Global Burden of Disease
CODEm	Cause of Death Ensemble model
GATHER	Guidelines for Accurate and Transparent Health Estimates Reporting
GHDx	Global Health Data Exchange
HIV	human immunodeficiency virus
HPV	human papillomavirus
ICD	International Classification of Diseases
LMICs	low- and middle-income countries
MEPCO	multinomial estimation with partial and composite observations
NTDs	neglected tropical diseases
RSV	respiratory syncytial virus
ST-GPR	Spatiotemporal gaussian process regression
UI	uncertainty interval
YLDs	years lived with disability
YLLs	years of life lost

Section 2: Data sources[1,2]

We use a subset of the input data described in the GB-AMR capstone paper.[1] This subset has information on underlying cause or primary diagnosis at admission or sample specimen type to determine the infectious syndrome which have a positive culture of pathogen and did not have a sampling framework that would bias the aetiology estimation towards a specific pathogen (ie, did not deliberately sample until 100 cases of every pathogen of interest had been obtained).

The input data source types that met these criteria were:

Section 2.1: Multiple causes of death and vital registration (MCoD-VR) data.

These are certificates from vital records provide the underlying, immediate and intermediate causes and conditions contributing to deaths observed in the following national health systems:

- United States National Vital Statistics System
- Brazil Mortality Information System
- National Institute of Statistics (Italy)
- Statistics South Africa
- National Institute of Statistics and Geography (Mexico)
- National Administrative Department of Statistics (Colombia)
- Taiwan Ministry of Health and Welfare

Section 2.2: Hospital discharge data.

Hospital admissions and discharge data, which include primary and secondary diagnosis for each patient.

• USA National Hospital Discharge Survey

• USA State Inpatient Databases

• Brazil Hospital Information System

• Italy Hospital Inpatient Discharges

• Sistema Automatizado de Egresos Hospitalarios (Mexico)

• Austria Hospital Inpatient Discharges

• New Zealand National Minimum Dataset

• Canada Discharge Abstract Database

Section 2.3: Linkage data sources.

For two of the hospital discharge sources mentioned above, namely Italy Friuli-Venezia Giulia and New Zealand National Minimum dataset, we have linked admission records to microbial positive cultures, which are referred as linkage data throughout the paper.

Section 2.4: Mortality surveillance in the Child Health and Mortality Prevention Surveillance (CHAMPS) study.

It comprises under-5 mortality surveillance in South Africa, Mali, Bangladesh, Kenya, Ethiopia, and Mozambique. This study provides information about pathogens contributing to death by collecting a minimally invasive tissue sampling (MITS) in addition to vital records. MITS is also known as a pathology-based autopsy which improves the understanding of mortality surveillance specially in low and middle income settings.

Section 2.5: Literature review of the microbial aetiology of meningitis, maternal and neonatal sepsis, lower respiratory infections, urinary tract infections, skin infections, peritonitis, and bone and joint infections

Search strings were used in PubMed to look systematically for the causative microorganisms of the following infectious syndromes:

Section 2.5.1: Meningitis

((meningitis[title]) AND (1990/05/01[PDat] : 2018/12/31[PDat]) AND ((etiolog*[title/abstract]) AND Humans[MeSH Terms])

Section 2.5.2: Maternal and neonatal sepsis and LRI aetiology

Aetiology terms, combined with OR:

- Infection (Infect*)
- Microbiology (Microbiolog*
- Aetiology (Aetiolog*)
- Etiology (Etiolog*)
- Virology (Virolog*)
- Bacteriology (Bacteriolog*)
- Fungus (fung*)

AND

Syndrome terms, combined with OR:

Maternal Sepsis

- puerperal sepsis (puerper* sepsis)

- maternal sepsis (matern* sepsis)
- puerperal septicaemia (puerper* septicaemia, American spelling too - septicemia)
- maternal septicaemia (matern* septicaemia, American spelling too - septicemia)
- puerperal infection (puerper* infection)
- maternal infection (matern* infection)
- puerperal bacteraemia (puerper* bacteraemia, American spelling too - bacteremia)
- maternal bacteraemia (matern* bacteraemia, American spelling too - bacteremia)

Neonatal Sepsis

- Neonatal sepsis (Neonat* sepsis within 3 or 5 words of each other)
- Neonatal septicaemia (Neonat* septicaemia within 3 or 5 words of each other, American spelling too - septicemia)
- Infant sepsis (Infant* sepsis)
- Infant septicaemia (Infant* septicaemia, American spelling too - septicemia)
- Neonatal bacteraemia (Neonat* bacteraemia, American spelling too - bacteremia)
- Infant bacteraemia (Infant* bacteraemia, American spelling too - bacteremia)

Lower respiratory infections

- LRI
- Lower respiratory infection
- LRTI
- Lower respiratory tract infection
- Pneumonia

Section 2.5.3: Urinary tract infections aetiology

("complicated"[Title/Abstract] OR "uncomplicated"[Title/Abstract]) AND (("Cystitis/etiology"[majr:noexp] OR "Cystitis/microbiology"[majr:noexp]) OR ("Pyelonephritis/etiology"[marj:noexp] OR "Pyelonephritis/microbiology"[majr:noexp]) OR ("Urinary Tract Infections/etiology"[majr:noexp] OR "Urinary Tract Infections/microbiology"[majr:noexp])) OR ("Urinary tract infections"[tiab] AND ("etiology"[tiab] OR "microbiology"[tiab]))

(("urinary tract infection*"[title]) AND (1990/05/01[PDat] : 2018/12/31[PDat]) AND ((etiolog*[title/abstract] OR "Urinary Tract Infections/microbiology"[Mesh]) AND Humans[MeSH Terms]) NOT Review[ptyp]

Section 2.5.4: Skin infections aetiology

(("Cellulitis/epidemiology"[majr:noexp] OR "Cellulitis/etiology"[majr:noexp] OR "Cellulitis/microbiology"[majr:noexp]) OR ("Pyoderma/epidemiology"[majr:noexp] OR "Pyoderma/etiology"[marj:noexp] OR "Pyoderma/microbiology"[majr:noexp]) OR

"Pressure Ulcer/microbiology"[majr:noexp])

(“skin and soft tissue infection”[title] OR cellulitis[title] OR erysipelas[title]) AND (1990/05/01[PDat] : 2018/12/31[PDat]) AND (etiolog*[title/abstract] OR "Cellulitis/microbiology"[Mesh]) AND Humans[MeSH Terms] NOT Review[ptyp]

Section 2.5.5: Intra-abdmoinal infection aetiology

(("Peritonitis/epidemiology"[majr:noexp] OR "Peritonitis /etiology"[majr:noexp] OR "Peritonitis /microbiology"[majr:noexp]) OR ("Intraabdominal infections/epidemiology"[majr:noexp] OR "Intraabdominal infections /etiology"[marj:noexp] OR "Intraabdominal infections /microbiology"[majr:noexp]) OR ("abdominal abscess/epidemiology"[majr:noexp] OR " abdominal abscess /etiology"[majr:noexp] OR "abdominal abscess/microbiology"[majr:noexp]))

Section 2.5.6: Bone and joint infections aetiology

("Osteomyelitis/etiology"[majr:noexp] OR "Osteomyelitis/microbiology"[majr:noexp] NOT 'chronic') OR ("Arthritis, infectious/etiology"[marj:noexp] OR "Arthritis, infectious/microbiology"[majr:noexp] NOT 'lyme')

Section 2.6: Exclusion criteria for literature reviews

Studies were excluded from full text review if:

- The study did not include at least one of the following: *E.coli, K.pneumoniae, S.pneumoniae, S.aureus or S.typhi/paratyphi*
- The entire study was conducted before 1990
- Samples were collected before 1990
- Did not perform resistance testing
- Sample is non-representative (lab strains, only resistant strains)
- Included non-human samples
- Article type was a case study
- Article type was a commentary, editorial or review with no primary data
- Isolates were not from blood culture
- There were duplicated isolates
- Travellers/non-endemic country/ no location information
- Study did not test susceptibility to antimicrobials
- There were fewer than 10 consecutive isolates used for susceptibility testing
- Could not locate the full text
- The study was uninterpretable due to poor data quality
- Studies where data was aggregated with other pathogens
- Studies using non-sterile site/mixed isolates
- Studies with no iNTS AST data

Section 2.7: Laboratory-based passive surveillance data.

Laboratories based in hospitals or part of public and private laboratory networks have provided information on patient's specimens with positive pathogen growth. We infer the infectious syndrome from admission diagnosis if this is present in data. If the former is not present, we use the type of specimen to infer the infectious syndrome of the patient. Some datasets include discharge disposition of the patient and whether the infection was identified after 48 hrs. from admission, which allow us to classify into community- or hospital-onset infections.

Section 2.7.1: Laboratory-based data with outcome:

- **USA Becton, Dickinson, and Co. (BD) Insights, Research and Analytics Database microbiology test and in-patient hospital data:** data procured by BD via MedMined. Covers a range of regions in the United States from 2011 to 2017.
- **International Nosocomial Infection Control Consortium (INICC) surveillance online system:** data from the INICC data collection software. ICU patient microbiology and hospital data from 50 countries across Latin America, Asia, the Middle East, eastern Europe, and Africa from 2009 to 2020.
- **St. George's Hospital, University of London - Global Antimicrobial Resistance, Prescribing and Efficacy among Neonates and Children (SGUL-GARPEC) Project bloodstream infection data:** Penta-sponsored global surveillance network focusing on neonatal and paediatric antimicrobial resistance and the organisms causing blood stream infections.
- **Burden of Antibiotic Resistance in Neonates from Developing Societies (BARNARDS)**: BARNARDS includes locations in Nigeria, South Africa, Pakistan, Rwanda, Bangladesh, Ethiopia and India from 2015 to 2018.
- **Lima, Peru Cayetano Heredia University (UPCH) antimicrobial resistance data:** data from UPCH hospital sites across Lima, Peru with discharge disposition for infectious pulmonary disease

Section 2.7.2: Laboratory-based data without outcome:

- **SENTRY:** SENTRY Antimicrobial Surveillance Program established by JMI Labs in 1997. Sites are in the USA, Europe, Latin America, parts of Asia, and the Western Pacific
- **Pfizer ATLAS Programme:** the Antimicrobial Testing Leadership and Surveillance (ATLAS) database includes the Tigecycline Evaluation Surveillance Trial (TEST), the Assessing Worldwide Antimicrobial Resistance Evaluation (AWARE) and the International Network for Optimal Resistance Monitoring (INFORM) programs. The study spans in coverage across more than 70 countries between 2004 and 2017.
- **WHO Meningitis surveillance:** sentinel hospital surveillance of suspected meningitis cases among children under 5 years old and positive cultures, provided by the World Health Organisation (WHO) Global Rotavirus, Invasive Bacterial Vaccine Preventable Diseases Surveillance Network Collaboration from 2008 to 2020.
- **NARMS**: The National Antimicrobial Resistance Monitoring System for Enteric Bacteria (NARMS) is a collaboration of agencies within The U.S. Department of Health and Human Services (HHS) (FDA and CDC) and the U.S. Department of Agriculture (USDA). It tracks enteric bacteria and selected animal pathogens and their resistance to antimicrobials, and data is available from 1997 onwards.
- **United States Active Bacterial Core Surveillance (ABCs) Reports:** case reports on healthcare-associated Infections and community interface infections from the Emerging Infections Program (EIP) Network coordinated by the Center for Disease Control and Prevention (CDC).
- **World Health Organization (WHO) Global Tuberculosis Programme**
- **GLASS**: Global Antimicrobial Resistance Surveillance System by WHO
- **Hospital Civil de Guadalajara Fray Antonio Alcalde, Mexico**
- **Canadian Antimicrobial Resistance Surveillance System**
- **SOAR**: Survey on Antibiotic Resistance (SOAR) sponsored by GSK.
- **ReLAVRA and SIREVA**: The Latin American Network for Antimicrobial Resistance Surveillance (ReLAVRA by its Spanish acronym) and the Serotype and Antimicrobial Resistance Surveillance Program (SIREVA by its English acronym) which are coordinated by the Pan-American Health Organization (WHO/PAHO)
- **SMART**: Study for Monitoring Antimicrobial Resistance Trends which monitors complicated intra-abdominal infections (cIAIs), complicated urinary tract infections (cUTIs) and respiratory infections worldwide, funded by Merck & Co.

Section 3: Supplementary methods: a summary of the estimation process[1,2]

Section 3.1: GBD 2019 framework

The study relies on Global Burden of Disease (GBD) 2019 fatal and non-fatal estimates, and a comprehensive description of data sources, data quality, statistical modelling and analyses for GBD 2019 have been reported elsewhere.[3] A brief summary of the fatal and non-fatal estimation, including a flow chart of the processes, can be found in the appendix of Murray et al. (2022).[1]

Section 3.2: Deaths where infection plays a role and infectious syndrome estimation

Section 3.2.1: Input data

Multiple causes of death (MCoD) data are individual-based records that provide underlying causes of death and two or more intermediate causes in the chain of death. Additionally, each record includes age, sex, residence, and the date of death.

Hospital record with multiple diagnoses and discharge status of death represents an individual-based hospital record of a patient that provides the main diagnosis and two or more additional diagnoses. Additionally, each record includes age, sex, residence, date of admission, date of discharge, and outcome (dead or alive). Only hospital discharges with discharge status of death were used in this component model, since we aimed to estimate the fraction of deaths that involve infection and the infectious syndrome distribution of those deaths.

Linkage data are generated using probabilistic methods in a defined population that link individual-based hospital data to individual-based MCoD data. Linkage data offer a wider dataset that includes main diagnosis, other diagnoses, underlying cause of death, and intermediate causes of death in the chain.

Section 3.2.2: Data processing and mapping

Within the WHO European region, data for Italy has been extracted at the subnational level by GBD 2019 age groups, sex, year, and causes of death and/or diagnoses, while data for the remaining countries have been analysed at the national level. This allowed us to expand the location-years of data that we had for each Socio-demographic Index (SDI)[4] value.

Prepared data were mapped to GBD causes. The GBD cause list is a mutually exclusive and collectively exhaustive list of diseases and injuries. The GBD cause list is organised hierarchically to accommodate different purposes and needs of various users. The first two levels aggregate causes into general groupings. At Level 1, there are three cause groups: communicable, maternal, neonatal, and nutritional diseases (Group 1 diseases); non-communicable diseases (Group 2); and injuries (Group 3). These Level 1 aggregates are subdivided at Level 2 of the hierarchy into 22 cause groupings (eg, neonatal disorders, neurological disorders, and transport injuries). The disaggregation into Levels 3 and 4 contains the finest level of detail for causes captured in GBD 2019. See section 14, table S1 for the full GBD cause hierarchy by level.

The underlying cause of death or main diagnosis for each record in the data was mapped to a GBD cause. After the mapping of underlying cause, we used the GBD 2019 garbage code redistribution algorithm (see appendix 1, section 2.4 in Vos et al.[3]) to ensure that all deaths had a plausible and specific underlying cause of death. The redistribution of garbage codes for underlying causes of death followed the same age and sex restrictions as GBD 2019. We did not redistribute garbage codes in the chain causes because the concept of a garbage code applies only to plausible underlying cause of death (see Rudd et al.[5] and appendix 1, section 2.5 in Vos et al.[3]).

Section 3.2.3: Intermediate cause and infectious syndrome mapping hierarchy with modelling pathways

Within our modelling framework, an infectious syndrome is the infection directly responsible for sepsis and serves as the bridge between the underlying cause of death and sepsis. Infectious syndromes can be both underlying causes of death and intermediate causes of death.

For mapping underlying and intermediate causes of death and hospital diagnoses to sepsis and infectious syndromes, we designed a new map, called "*AMR, sepsis, and infectious syndrome map*". This map is a list of mutually exclusive and collectively exhaustive infectious syndromes that we divided into four levels to form the infectious syndrome hierarchy.

Each level of infectious syndrome is mutually exclusive and collectively exhaustive. Furthermore, the infectious syndrome hierarchy is internally consistent across any metric (eg, number, cause fraction)—aggregating across Level 3 syndromes gives us Level 2 syndromes, aggregating the Level 2 syndromes gives us Level 1 syndromes, and the total of Level 1 syndromes is equal to the value of sepsis (figure 4.4.2.1).

Level 0: All International Classification of Diseases 9th (ICD-9) or 10th revision (ICD-10) coded deaths divided into three groups: explicit sepsis (any death with the specific ICD code for sepsis in the MCoD chain or hospital diagnoses), implicit sepsis (any death with an infectious disease code in the underlying cause or cause chain, as well as with a specific organ dysfunction) and non-sepsis (any death that does not meet either of the two aforementioned criteria). More information can be found in the appendix of Murray et al. (2022).[1]

Explicit sepsis (A40, R65.2 in ICD-10 and 039 in ICD-9): Any death has specific ICD code for sepsis in the MCoD chain or hospital diagnoses was considered explicit sepsis.[5]

- Implicit sepsis: Any death that has an infectious disease code in the underlying cause or cause chain and a specific organ dysfunction code was considered implicit sepsis
- Non-sepsis: Any death that does not meet either of the two above criteria (section 14, tables S2, S3)

Of the estimated infection-related deaths with explicit sepsis or implicit sepsis and infectious diseases, 59.4% occur with communicable, maternal, neonatal, and nutritional underlying causes of death. 38.9% infection related deaths occur with non-communicable disease as the underlying cause of death, and 1.7% occur with injuries as the underlying cause of death.

Level 1: All implicit and explicit sepsis deaths were divided into 12 Level 1 infectious syndromes and an "other" category. These are as follows: 1) Bacterial infections of the skin and subcutaneous systems; 2) Bloodstream infections; 3) Gonorrhoea and chlamydia; 4) Diarrhoea; 5) Endocarditis and other cardiac infections; 6) Infections of bones, joints and related organs; 7) Lower respiratory infections and all related infections in the thorax; 8) Meningitis and other bacterial central nervous system infections; 9) Peritoneal and intra-abdominal infections; 10) Tuberculosis; 11) Typhoid, paratyphoid, and invasive non-typhoidal *Salmonella;* 12) Urinary tract infection and pyelonephritis; 13) Other infections

Level 2: Each Level 1 infectious syndrome was divided into Level 2 infectious syndromes based on the pathogen type (eg, bacterial, fungal, viral) causing the infection. Examples include specified bacterial, unspecified bacterial, fungal, viral, and unspecified pathogen.

Level 3: Each specified bacterial infectious syndrome in Level 2 was divided to Level 3 infectious syndromes by the culprit bacterial pathogen. Table S3 (section 14) shows this list and bacterial hierarchy.

Due to our data often having multiple diagnoses associated with each record, a single case of sepsis could potentially map to multiple candidate infectious syndromes. Because multiple infectious syndrome assignments pose a risk of double counting, we employed an informative ranking hierarchy. The informative ranking allowed us to determine the infectious syndrome that provided the most information on the culprit pathogen. The goal of this hierarchy was to produce the most accurate pathogen burden estimate such that when there were multiple infectious syndromes, we prioritised the syndrome with the most distinctive distribution. For example, bloodstream infections (BSIs) are common infections in sepsis but there is often an earlier source of the infection such as a UTI, cellulitis, or LRI, and each has a unique pathogen distribution that provides more information than the distribution of BSI. In the event that a patient record reflected both BSI and LRI, we would assign the infectious syndrome based on the pathogen distribution that would be the most proximal aetiologic syndrome, LRI (please refer to the appendix of Murray et al. (2022)[1] for more information).

After mapping the underlying and chain causes of death, our database went through two separate modelling pathways. The first model estimated the fraction of deaths that are sepsis-related in each GBD cause; these sepsis-related deaths for non-infectious GBD causes were combined with GBD deaths for infectious causes to create the total envelope of all deaths where infection plays a role. The second pathway estimated each infectious syndrome as a fraction of sepsis-related mortality in each GBD cause. In the last step of infectious syndrome estimation, the fractions of sepsis by Level 1 infectious syndromes were squeezed to sum to one so as to not exceed the sepsis mortality envelope and multiplied by the sepsis estimate in each GBD cause by country and territory, age, and sex in 2019.

Section 3.2.4: First pathway – deaths where infection plays a role

We used a mixed-effects binomial logistic regression to model the logit of the fraction of sepsis-related deaths by GBD cause-age-sex-location, consistent with the modelling approach used by Rudd et al.[5] Sex and Healthcare Access and Quality Index (HAQ Index)[3] were included as covariates and a nested random effect on underlying cause of death was included. A separate model was run for each GBD 2019 age group (0–6, 7–27, 28-364 [days], 1–4, 5–9, 10–14, 15–19, 20–24, 25–29, 30–34, 35–39, 40–44, 45–49, 50–54, 55–59, 60–64, 65–69, 70–74, 75–79, 80–84, 85–89, 90–94, 95+ [years]):

$$sepsis\ related\ deaths \sim B(total\ deaths, sepsis\ fraction)$$

$$logit(sepsis\ fraction) = \beta_0 + \beta_1 * HAQ\ Index + \beta_2 * sex + \pi_{level\ 1, level\ 2}$$

Where $\pi_{level\ 1, level\ 2}$ is a nested random effect on underlying cause of death. The nested random-effect's structure in the model on underlying cause of death allowed the prediction of sepsis fractions where data were limited by borrowing information from diseases within the same group. There were 22 groups of underlying causes of death, each categorised by physiological relatedness. We produced our predictions and uncertainty intervals (UIs) by generating 1000 draws from the normal distribution of the fixed coefficients, separately for each GBD location, age group, sex, and cause in 2019. The means of our results were used for the point estimates and the 95% UIs were

delineated using the 2.5th and 97.5th percentiles of the draws. Uncertainty is attributable to sample size variability between data sources, data availability, and model specifications.

All underlying causes of death that are infectious diseases were included in the model; however, for these causes we used the GBD death estimates rather than the modelled sepsis estimate, since infection inherently plays a role in these deaths even if the pathway doesn't include sepsis. These causes and their associated infectious syndromes are available in the appendix of Murray et al. (2022).[1] For all other causes, we calculated the number of sepsis-related deaths in 2019 by multiplying our predictions of cause-, age group-, sex-, year-, and location-specific sepsis fractions by GBD 2019 death estimates. Finally, we aggregated our results to arrive at regional and global sepsis-related mortality in non-infectious underlying causes of death, which we combined with the GBD infectious disease deaths estimates to create the mortality envelope of all deaths related to infection.

Section 3.2.5: Second pathway – fraction of deaths where infection plays a role by infectious syndrome in each GBD cause

We used a mixed-effects binomial logistic regression to model the logit of the infectious syndrome fraction of sepsis-related mortality by GBD cause. The model covariates varied by infectious syndrome, and all models included HAQ Index as a covariate and most included a summary exposure value (SEV) scalar calculated for GBD 2019. To more accurately estimate the burden of pathogens responsible for infection, we separated infectious syndromes into hospital-acquired and community-acquired for LRI+ and UTI. More details on the infectious syndrome model covariates and age groups are found in the appendix of Murray et al. (2022).[1]
The infectious syndrome models were specified as mixed-effects binomial logistic regressions, one for each infectious syndrome and age group:

$$syndrome\ related\ deaths \sim B(total\ sepsis\ deaths, syndrome\ fraction)$$

$$logit(syndrome\ fraction) = \beta_0 + \beta * X + \pi_{level\ 1, level\ 2}$$

where β and X are vectors of length for covariates and is a nested random effect on underlying cause of death. The granularity of the age groups estimated for each infectious syndrome was chosen based on the age pattern of the infectious syndrome and the limitations of data sparsity.

As in the first pathway, we derived our predictions and UIs by generating 1000 draws from the normal distribution of the fixed coefficients separately for each GBD location, age group, sex, and cause in 2019. We used the means of our results for the point estimates and the 95% UIs were delineated using the 2.5th and 97.5th percentiles of the draws.

We calculated the number of deaths attributable to each infectious syndrome in 2019 by multiplying our predictions of cause-, age group-, sex-, year-, and location-specific infectious syndrome fractions by our sepsis-mortality estimates from the first pathway. All infectious syndrome fractions were squeezed to sum to one prior to multiplication in order to ensure that we did not exceed the sepsis mortality envelope.

Out of the 12 explicit Level 1 infectious syndromes included in our hierarchy, we excluded (i) tuberculosis (TB), (ii) typhoid, paratyphoid, and invasive non-typhoidal *Salmonella*, and (iii) gonorrhoea and chlamydia from our binomial mixed-effects linear regression model. Instead, we used the published results from GBD 2019[4] for these causes of death, as we believe the GBD 2019 estimates fully represent these infectious syndromes because they are usually not intermediate causes of death.

Section 3.2.6: Model validation

Infectious syndrome modelling aims to predict which cases of infection belong to a specific infectious syndrome, which is a multi-class classification problem. We therefore use the Area Under the Receiver Operating Characteristics (ROC) Curve (AUC) and accuracy to evaluate model performance. More information on this can be found in the appendix of Murray et al. (2022).[1]

The out-of-sample strategy for this validation excluded 20% of the sample on each iteration. Table 3.2.6.1 reports the Accuracy and AUC score[6] for each of the age groups within the infectious syndrome models and table 3.2.6.2

reports the same metrics for the sepsis models. 99% of the models have an AUC score between 0.7 and 1, indicating an overall excellent performance of this modelling framework.

Table 3.2.6.1: Accuracy and AUC score for out-of-sample validation of infectious syndrome models (GLOBAL)

Model	Age group name	Accuracy	AUC score
CAI lower respiratory infections and all related infections in the thorax	Post Neonatal to 5	0.99	1.00
CAI lower respiratory infections and all related infections in the thorax	70+ years	0.99	1.00
CAI urinary tract infections and pyelonephritis	0 to 39	1.00	1.00
CAI urinary tract infections and pyelonephritis	40 plus	1.00	1.00
Diarrhoea	Early Neonatal	1.00	1.00
Diarrhoea	Late Neonatal	1.00	1.00
Diarrhoea	1 to 4	0.99	1.00
Diarrhoea	20 to 24	0.99	1.00
Diarrhoea	25 to 29	0.99	1.00
Diarrhoea	30 to 34	0.99	1.00
Diarrhoea	35 to 39	0.99	1.00
CAI lower respiratory infections and all related infections in the thorax	5 to 69	0.99	0.99
Diarrhoea	Post Neonatal	0.98	0.99
Diarrhoea	5 to 9	0.99	0.99
Diarrhoea	10 to 14	0.99	0.99
Diarrhoea	15 to 19	0.99	0.99
Diarrhoea	40 to 44	0.99	0.99
Diarrhoea	45 to 49	0.99	0.99
Diarrhoea	95 plus	0.99	0.99
Meningitis and other bacterial central nervous system infections	Early Neonatal	0.99	0.99
Meningitis and other bacterial central nervous system infections	Late Neonatal	1.00	0.99
Bacterial infections of the skin and subcutaneous systems	Late Neonatal	0.99	0.98
Diarrhoea	50 to 54	0.99	0.98
Diarrhoea	85 to 89	0.99	0.98
Diarrhoea	90 to 94	0.99	0.98
Endocarditis and other cardiac infections	Early Neonatal	0.99	0.98
Endocarditis and other cardiac infections	Late Neonatal	0.99	0.98
Endocarditis and other cardiac infections	85 to 89	0.99	0.98
Endocarditis and other cardiac infections	90 to 94	0.99	0.98
Endocarditis and other cardiac infections	95 plus	0.99	0.98
Meningitis and other bacterial central nervous system infections	Post Neonatal	0.99	0.98
Meningitis and other bacterial central nervous system infections	1 to 4	0.98	0.98
Meningitis and other bacterial central nervous system infections	10 to 14	0.97	0.98
Meningitis and other bacterial central nervous system infections	25 to 29	0.99	0.98
Meningitis and other bacterial central nervous system infections	30 to 34	0.99	0.98
Peritoneal and intra-abdominal infections	25 to 29	0.98	0.98
Peritoneal and intra-abdominal infections	30 to 34	0.98	0.98

Peritoneal and intra-abdominal infections	35 to 39	0.98	0.98
Peritoneal and intra-abdominal infections	80 to 84	0.98	0.98
Peritoneal and intra-abdominal infections	85 to 89	0.98	0.98
Peritoneal and intra-abdominal infections	90 to 94	0.98	0.98
Peritoneal and intra-abdominal infections	95 plus	0.99	0.98
Bacterial infections of the skin and subcutaneous systems	95 plus	0.98	0.97
Diarrhoea	55 to 59	0.99	0.97
Diarrhoea	60 to 64	0.99	0.97
Diarrhoea	75 to 79	0.99	0.97
Diarrhoea	80 to 84	0.99	0.97
Endocarditis and other cardiac infections	10 to 14	0.99	0.97
Endocarditis and other cardiac infections	25 to 29	0.99	0.97
Endocarditis and other cardiac infections	30 to 34	0.99	0.97
Endocarditis and other cardiac infections	35 to 39	0.99	0.97
Endocarditis and other cardiac infections	40 to 44	0.99	0.97
Endocarditis and other cardiac infections	80 to 84	0.99	0.97
Meningitis and other bacterial central nervous system infections	5 to 9	0.97	0.97
Meningitis and other bacterial central nervous system infections	15 to 19	0.98	0.97
Meningitis and other bacterial central nervous system infections	20 to 24	0.99	0.97
Meningitis and other bacterial central nervous system infections	35 to 39	0.99	0.97
Peritoneal and intra-abdominal infections	Early Neonatal	0.99	0.97
Peritoneal and intra-abdominal infections	Late Neonatal	0.99	0.97
Peritoneal and intra-abdominal infections	1 to 4	0.99	0.97
Peritoneal and intra-abdominal infections	5 to 9	0.98	0.97
Peritoneal and intra-abdominal infections	20 to 24	0.97	0.97
Peritoneal and intra-abdominal infections	40 to 44	0.97	0.97
Peritoneal and intra-abdominal infections	75 to 79	0.97	0.97
Bacterial infections of the skin and subcutaneous systems	90 to 94	0.98	0.96
Bloodstream infections	Early Neonatal	0.94	0.96
Bloodstream infections	Late Neonatal	0.95	0.96
Bloodstream infections	Post Neonatal	0.93	0.96
CAI lower respiratory infections and all related infections in the thorax	Neonatal	0.95	0.96
Diarrhoea	65 to 69	0.99	0.96
Diarrhoea	70 to 74	0.99	0.96
Endocarditis and other cardiac infections	15 to 19	0.99	0.96
Endocarditis and other cardiac infections	20 to 24	0.99	0.96
Endocarditis and other cardiac infections	45 to 49	0.99	0.96
Endocarditis and other cardiac infections	50 to 54	0.99	0.96
Endocarditis and other cardiac infections	70 to 74	0.99	0.96
Endocarditis and other cardiac infections	75 to 79	0.99	0.96
Meningitis and other bacterial central nervous system infections	40 to 44	0.99	0.96

Meningitis and other bacterial central nervous system infections	45 to 49	0.99	0.96
Peritoneal and intra-abdominal infections	10 to 14	0.97	0.96
Peritoneal and intra-abdominal infections	15 to 19	0.96	0.96
Peritoneal and intra-abdominal infections	45 to 49	0.97	0.96
Peritoneal and intra-abdominal infections	70 to 74	0.97	0.96
Bacterial infections of the skin and subcutaneous systems	30 to 34	0.99	0.95
Bacterial infections of the skin and subcutaneous systems	85 to 89	0.98	0.95
Bloodstream infections	1 to 4	0.91	0.95
Bloodstream infections	95 plus	0.94	0.95
Endocarditis and other cardiac infections	5 to 9	0.99	0.95
Endocarditis and other cardiac infections	55 to 59	0.99	0.95
Endocarditis and other cardiac infections	60 to 64	0.99	0.95
Endocarditis and other cardiac infections	65 to 69	0.99	0.95
Infections of bone, joints, and related organs	10 to 14	0.99	0.95
Infections of bone, joints, and related organs	95 plus	0.99	0.95
Peritoneal and intra-abdominal infections	50 to 54	0.96	0.95
Peritoneal and intra-abdominal infections	55 to 59	0.96	0.95
Peritoneal and intra-abdominal infections	60 to 64	0.96	0.95
Peritoneal and intra-abdominal infections	65 to 69	0.96	0.95
Bacterial infections of the skin and subcutaneous systems	Early Neonatal	0.99	0.94
Bacterial infections of the skin and subcutaneous systems	25 to 29	0.99	0.94
Bacterial infections of the skin and subcutaneous systems	35 to 39	0.99	0.94
Bacterial infections of the skin and subcutaneous systems	40 to 44	0.98	0.94
Bacterial infections of the skin and subcutaneous systems	80 to 84	0.98	0.94
Bloodstream infections	5 to 9	0.87	0.94
Bloodstream infections	20 to 24	0.89	0.94
Bloodstream infections	25 to 29	0.92	0.94
Bloodstream infections	30 to 34	0.93	0.94
Endocarditis and other cardiac infections	1 to 4	0.99	0.94
HAI lower respiratory infections and all related infections in the thorax	Post Neonatal to 5	0.97	0.94
Infections of bone, joints, and related organs	0 to 9	0.99	0.94
Infections of bone, joints, and related organs	85 to 89	0.99	0.94
Infections of bone, joints, and related organs	90 to 94	0.99	0.94
Meningitis and other bacterial central nervous system infections	50 to 54	0.99	0.94
Peritoneal and intra-abdominal infections	Post Neonatal	0.98	0.94
Bacterial infections of the skin and subcutaneous systems	20 to 24	0.99	0.93
Bacterial infections of the skin and subcutaneous systems	45 to 49	0.98	0.93
Bacterial infections of the skin and subcutaneous systems	75 to 79	0.98	0.93
Bloodstream infections	35 to 39	0.92	0.93
Bloodstream infections	90 to 94	0.94	0.93
Infections of bone, joints, and related organs	80 to 84	0.99	0.93

Meningitis and other bacterial central nervous system infections	55 to 59	0.99	0.93
Meningitis and other bacterial central nervous system infections	60 to 64	0.99	0.93
Meningitis and other bacterial central nervous system infections	90 to 94	0.99	0.93
Bacterial infections of the skin and subcutaneous systems	50 to 54	0.98	0.92
Bacterial infections of the skin and subcutaneous systems	55 to 59	0.97	0.92
Bacterial infections of the skin and subcutaneous systems	60 to 64	0.97	0.92
Bacterial infections of the skin and subcutaneous systems	65 to 69	0.97	0.92
Bacterial infections of the skin and subcutaneous systems	70 to 74	0.98	0.92
Bloodstream infections	10 to 14	0.85	0.92
Bloodstream infections	40 to 44	0.90	0.92
Bloodstream infections	85 to 89	0.93	0.92
Infections of bone, joints, and related organs	75 to 79	0.99	0.92
Meningitis and other bacterial central nervous system infections	65 to 69	0.99	0.92
Meningitis and other bacterial central nervous system infections	70 to 74	0.99	0.92
Meningitis and other bacterial central nervous system infections	80 to 84	0.99	0.92
Meningitis and other bacterial central nervous system infections	85 to 89	0.99	0.92
Meningitis and other bacterial central nervous system infections	95 plus	0.99	0.92
Bloodstream infections	15 to 19	0.84	0.91
Bloodstream infections	80 to 84	0.92	0.91
Infections of bone, joints, and related organs	70 to 74	0.99	0.91
Meningitis and other bacterial central nervous system infections	75 to 79	0.99	0.91
Bacterial infections of the skin and subcutaneous systems	15 to 19	0.98	0.90
Bloodstream infections	45 to 49	0.89	0.90
Infections of bone, joints, and related organs	60 to 64	0.99	0.90
Infections of bone, joints, and related organs	65 to 69	0.99	0.90
Bacterial infections of the skin and subcutaneous systems	Post Neonatal	1.00	0.89
Bloodstream infections	50 to 54	0.88	0.89
Bloodstream infections	75 to 79	0.91	0.89
Endocarditis and other cardiac infections	Post Neonatal	0.99	0.89
HAI lower respiratory infections and all related infections in the thorax	5 to 69	0.96	0.89
HAI lower respiratory infections and all related infections in the thorax	70+ years	0.96	0.89
Infections of bone, joints, and related organs	55 to 59	0.99	0.89
Bloodstream infections	70 to 74	0.90	0.88
Infections of bone, joints, and related organs	15 to 19	0.99	0.88
Infections of bone, joints, and related organs	50 to 54	0.99	0.88
Bacterial infections of the skin and subcutaneous systems	1 to 4	1.00	0.87
Bacterial infections of the skin and subcutaneous systems	5 to 9	0.99	0.87
Bacterial infections of the skin and subcutaneous systems	10 to 14	0.99	0.87
Bloodstream infections	55 to 59	0.88	0.87
Bloodstream infections	60 to 64	0.88	0.87
Bloodstream infections	65 to 69	0.89	0.87

HAI urinary tract infections and pyelonephritis	40 plus	0.99	0.86
Infections of bone, joints, and related organs	25 to 29	0.99	0.85
Infections of bone, joints, and related organs	35 to 39	0.99	0.85
Infections of bone, joints, and related organs	40 to 44	0.99	0.84
Infections of bone, joints, and related organs	45 to 49	0.99	0.84
Infections of bone, joints, and related organs	30 to 34	0.99	0.83
Infections of bone, joints, and related organs	20 to 24	0.99	0.82
HAI urinary tract infections and pyelonephritis	0 to 39	0.99	0.77
HAI lower respiratory infections and all related infections in the thorax	Neonatal	0.99	0.50

Table 3.2.6.2: Accuracy and AUC score for out-of-sample validation of sepsis models (GLOBAL)

**Model**	**Age group name**	**Accuracy**	**AUC score**
Sepsis	25 to 29	0.94	0.95
Sepsis	15 to 19	0.95	0.94
Sepsis	20 to 24	0.95	0.94
Sepsis	30 to 34	0.93	0.94
Sepsis	1 to 4	0.89	0.93
Sepsis	35 to 39	0.93	0.93
Sepsis	5 to 9	0.89	0.92
Sepsis	10 to 14	0.90	0.92
Sepsis	95 plus	0.96	0.92
Sepsis	40 to 44	0.93	0.91
Sepsis	90 to 94	0.96	0.90
Sepsis	Post Neonatal	0.88	0.89
Sepsis	Late Neonatal	0.87	0.88
Sepsis	45 to 49	0.93	0.88
Sepsis	85 to 89	0.96	0.88
Sepsis	Early Neonatal	0.91	0.87
Sepsis	80 to 84	0.96	0.87
Sepsis	50 to 54	0.93	0.86
Sepsis	75 to 79	0.95	0.85
Sepsis	55 to 59	0.94	0.84
Sepsis	70 to 74	0.95	0.84
Sepsis	60 to 64	0.94	0.83
Sepsis	65 to 69	0.94	0.83

Section 3.3: Case fatality ratios

Section 3.3.1: Input data

Case fatality ratios (CFRs) were modelled for the pathogens and infectious syndromes of interest using all available data detailing the organism responsible for infection, the infectious syndrome, and patient outcome, which included hospital and microbial data. Input data for the CFR models were aggregated based on data source, year, GBD location, and age group (as well as hospital/community acquired status, in the case of the lower respiratory and

urogenital infectious models). For lower respiratory and blood stream infections, for which CFRs could be vastly different in neonates, we modelled the following age groups: neonatal, post-neonatal–5 years, 5–50 years, 50–70 years, and 70 years and older. For all other infectious syndromes, we modelled the following age groups: neonatal–5 years, 5–50 years, 50–70 years, and 70 years and older. We excluded from the analysis any source-location-year-age with fewer than five cases and zero deaths.

To allow us to implement linear models, CFRs were logit-transformed. We used the delta method to compute the standard error of CFRs in logit space. To incorporate data with zero deaths, or with an equal number of deaths and cases, we applied a 1% offset, such that the CFRs for data with zero deaths was represented as 1% and the CFR for data with an equal number of deaths and cases was represented as 99%.

Pathogen-specific CFRs were modelled separately by infectious syndrome and were calculated as a function of HAQ Index and age. To account for heterogeneity across the sources of input data, we implemented a mixed-effects meta-regression framework, modelling data source as a random effect. We further incorporated a binary fixed-effect denoting whether the data source only included intensive care unit (ICU) patients, for which CFRs were expected to be higher. The pathogens of interest for each infectious syndrome were determined by prevalence in the data and expert opinion, with the goal of modelling approximately 90% of specified-pathogens associated with each infectious syndrome.

Section 3.3.2: Models run for each infectious syndrome

The interaction of the HAQ Index fixed-effect with the pathogen-specific fixed-effect allowed the relative deadliness of pathogens to vary depending on a location's HAQ Index – this is termed an 'interaction model'. For those pathogens with fewer than ten high quality data points below 0.7 HAQ Index, or those whose results in the interaction models indicated an unrealistically large influence of HAQ Index (eg, 70% CFR in low HAQ Index countries, 1% CFR in high HAQ Index countries), we modelled a pathogen-specific intercept with an HAQ Index fixed-effect shared across the pathogens. As a consequence of the single fixed-effect on HAQ Index, a pathogen that was predicted to be the deadliest in low HAQ Index countries would also be predicted to be the deadliest in high HAQ Index countries in these 'intercept models.' To estimate the CFRs for other known bacteria, which either were not selected as a pathogen of interest or lacked sufficient data for inclusion in the intercept models, we pooled all bacterial data together and estimated a single CFR curve from age, HAQ Index, and the data source heterogeneity covariates. Thus, up to three models were run for each infectious syndrome:

1. an interaction model including data for all data rich pathogens and 'other specified bacteria' (which was included to inform the overall influence of HAQ Index on CFR, predictions were only generated for the data rich pathogens),
2. an intercept model including data for data rich and data sparse pathogens, as well as 'other specified bacteria' (predictions were only generated for the data sparse pathogens), and
3. an 'other bacteria' model that included data for all bacterial pathogens (predictions were generated by HAQ Index and age, without any pathogen specific term).

For some infectious syndromes, the relative deadliness of a pathogen may be strongly determined by either the age of the patient or whether the infection was community- or hospital-acquired. For bloodstream infections, we ran two distinct sets of CFR models, one for neonates (0–27 days) and another for post neonates, to capture the differing dynamics of pathogen deadliness in these two populations. As is done for our other modelling processes, we also separate community-acquired and hospital-acquired cases in our CFR models for lower respiratory and urogenital infections. Because some data sources did not provide enough information to infer whether an infection was community- or hospital-acquired, but still included important information on the relative pathogenesis and the difference in CFRs across varying HAQ indices, infections of unknown origin were included in both the community-acquired and hospital-acquired models for these two syndromes. Any bias in these 'unknown origin' infections was adjusted for using a binary fixed-effect representing an 'unknown origin' infection, and predictions were generated for the community- and hospital-acquired infections only.

Section 3.3.3: Modelling framework

The data were analysed using a meta-analytic mixed effects structure. The main model can be specified as follows:

$$logit(y_i) = X_i\beta + u_i 1 + \epsilon_i, \;\; \epsilon_i \sim N(0, \Sigma_i), \quad u_i \sim N(0, \gamma)$$

where

- y_i contains CFRs for data source i
- Design matrix contains as columns the following covariates
  - in all models:
    - HAQ Index
    - dummy-coded indicator for age group
    - dummy-coded ICU indicator for data source (1 if data source only compiles information on ICU patients, 0 if a mix between ICU/non-ICU patients)
  - in 'interaction' and 'intercept' models:
    - dummy-coded indicator for pathogen
  - in 'interaction' models only:
    - interaction between pathogen and HAQ Index (product of dummy-coded pathogen columns and HAQ Index)
  - in models evaluating community/hospital acquired infection (LRI+, UTI):
    - dummy-coded variable indicating source of infection (1 if unknown source, 0 if community OR hospital acquired, depending on whether the model is evaluating community or hospital infections)
- β are fixed effect multipliers
- ϵ_i are observation error terms with known variances
- u_i are data source-specific random intercepts with unknown covariance γ

The underlying program used to fit the model (meta-regression, Bayesian, regularized, trimmed [MR-BRT]) is described elsewhere.[7] The program allows specification of priors on γ and β, which were particularly useful when data for specific locations was very limited.

Section 3.3.4: Predictions and uncertainty

Predictions for 2019 CFRs were generated for each country, age group, and pathogen as a function of each country's HAQ Index, assuming mixed ICU/non-ICU patients and, in the case of models for UTI and LRI+, that the infection was community- or hospital-acquired (in contrast to infections of unknown origin). For pathogens with insufficient data to estimate a syndrome-specific CFR, we predicted out using the 'other bacteria' CFR associated with the infectious syndrome. Importantly, all of the CFRs we calculate by infectious syndrome are independent of that syndrome's underlying cause.

Uncertainty estimates were generated using asymptotic uncertainty intervals. Specifically, for the model, the posterior uncertainty for the coefficients β is Gaussian, with mean and variance given below:

$$\hat{\beta} = \left(\sum_i X_i^T V_i^{-1} X_i\right)^{-1} \left(\sum_i X_i^T V_i^{-1} logit(y_i)\right)$$

$$Var(\hat{\beta}) = \left(\sum_i X_i^T V_i^{-1} X_i\right)^{-1}$$

where

$$V_i = 11^T + \hat{\gamma} I$$

where $\hat{\gamma}$ is the estimated variance of random effects and $Var(\hat{\beta})$ refers to the estimated variance-covariance matrix of beta.

The variance-covariance matrix was used to obtain 1000 draws for the coefficients, which are then used to get intervals for the predictions.

Section 3.3.5: Modeling exceptions for lower respiratory infections and all related infections in the thorax

To reduce the effect of bias from severe cases, we controlled for data provided from ICU-only sources which, if left uncontrolled, bias the results towards higher CFRs. Additionally, we controlled for data with unknown setting of infection origin due to our process of modeling community- and hospital-acquired lower respiratory infections separately.

We used a Poisson family model in which the probability distribution took the form of:

$$P(y_i|\lambda_i) = \frac{1}{y_i!}\exp(-\lambda_i)\,\lambda_i^{y_i} = \frac{1}{y_i!}\exp(-\lambda_i + y_i\log(\lambda_i))$$

Where *y* is the number of deaths. This suggests the following parameterization of:

$$\log(\lambda_i) = c_i + x_i^T\beta.$$

The link function is the exponential map and $x_i^T\beta$ is a linear predictor that can use direct covariates or splines. $c_i$is an offset used for observation-specific normalization of the number of cases, thereby allowing us to model rates.

β is estimated using the following:

$$\min_{\beta} \sum_i \exp\left(c_i + x_i^T\beta\right) - y_i\left(c_i + x_i^T\beta\right)$$

And the following priors were used to constrain the coefficients:

- Pathogen-vaccination interaction: We assumed vaccination would have no impact on CFRs of unrelated pathogens. For all combinations of pathogen-vaccination interaction that were not *Streptococcus pneumoniae*:PCV vaccination or *Haemophilus influenzae*:Hib vaccination, we coerced the the coefficients to 0 using model priors. For the *Streptococcus pneumoniae*:PCV vaccination and *Haemophilus influenzae*:Hib vaccination interaction terms, we employed a negativity prior to enforce case-fatality rates for these pathogens to decrease as vaccination was introduced.
- Large data source dummy variables: A variable for the data source was included to account for source heterogeneity. It is important to note that many input data sources covered only a single country, leading to low variability in HAQ Index within each data source. Such collinearity adversely influenced the accuracy of the estimated effect of HAQ Index, which was instrumental in extrapolating trends from the input data to global results. To emphasise the contribution of HAQ Index over data-source in the modelled estimates, we implemented a Gaussian prior (mean 0, standard error 0.1) on the coefficients for data source variables.

Nonfatal pathogen proportions for a given demographic group and pathogen were converted to deaths using the CFRs estimates for demographic group as follows:

$$p_{i,j}^{deaths} = \frac{p_{i,j} \times CFR_i}{\sum_{J_{i,J}^P} CFR_i}$$

Finally, we adjusted influenza and RSV mortality estimates for 2020 and 2021 to account for the reductions in influenza and RSV cases associated with the COVID-19 pandemic, as described elsewhere in this appendix.

Section 3.4: Pathogen distribution

Section 3.4.1: Input data and pathogens selected for estimation

With this model, we aimed to estimate the distribution of pathogens causing each infectious syndrome. To get input data for this model, we gathered all available data sources described in section 2 that meet the following criteria:

- Sufficient diagnosis (for patient- or admission-level datasets) or sample specimen type (for isolate- or culture-level datasets) information for us to determine the infectious syndrome

- Information on which pathogen(s) caused the infection or which pathogen(s) were detected in an infectious sample, as determined through culture or genomic-based methods
- Did not have a strongly biased sampling framework across pathogens (for example, did not deliberately sample until 100 cases of every pathogen of interest had been obtained)

The input data source types that met these criteria in this study were:

- Multiple causes of death data
- Hospital discharge
- Linkage data
- Microbial data with and without outcome information
- Literature studies from the aetiology literature reviews

For each infectious syndrome, we selected roughly 10–20 pathogens to estimate explicitly in the pathogen distribution based on the following criteria:

- The prevalence of each pathogen in the raw data
- Clinical knowledge about the primary aetiologies of each infectious syndrome
- The amount of available data, which limits the number of pathogens that can be estimated successfully

In addition to the n pathogens for a given syndrome that we estimate explicitly, we also included an "other specified pathogens" category for every infectious syndrome, to which we mapped all other aetiologies identified in the data. Thus, the set of estimated pathogens for each infectious syndrome is mutually exclusive and collectively exhaustive of all possible aetiologies. Polymicrobial infections were either estimated explicitly or included in the "other" category, making all explicitly estimated individual pathogens mono-pathogenic. In addition to these criteria, we also considered the following factors:

- Since we were ultimately interested in estimating the burden of AMR in bacteria, we erred on the side of estimating bacteria with strong evidence of AMR, rather than bacteria with low evidence of AMR or non-bacterial aetiologies.
- Clinically relevant aetiologies differ from syndrome to syndrome, and we were unable to estimate all pathogens explicitly in every syndrome due to a lack of data. Therefore, the "other" pathogen category is composed of slightly different pathogens for every infectious syndrome and can occasionally contain pathogens that are explicitly estimated for another infectious syndrome. We attempted to mitigate this by including bacteria with strong evidence of AMR in the estimation of all infectious syndromes whenever possible.
- We included enough explicitly estimated pathogens to ensure that the "other" category remained below 10% for all infectious syndromes.

For a list of pathogens covered in each infectious syndrome model, please refer to table 3.4.6 (pp 23-24).

Section 3.4.2: Data processing and analysis

We extracted and standardised the location, year, age, sex, diagnoses, specimen type, pathogens, and hospital- and community-acquired (HAI and CAI) status of each record in every dataset. These datasets report a variety of metrics, including deaths, admissions, cases, cultures, and isolates. While these metrics are not completely comparable (for example, a single patient may often have multiple cultures taken during a single hospital admission), we chose to standardise them into two categories: "deaths," for any unit associated with an outcome of death, and "cases," for any unit regardless of outcome. After standardising the data, we mapped every sample ID or tabulated figure in the data to infectious syndrome based on its diagnoses and specimen type. More details on this process can be found the appendix of Murray et al. (2022).[1]

Some pathogens cause disease so rarely or are so commonly contaminants that we considered them to be contaminants, unlikely to be the true cause of disease. Examples include many *Corynebacterium* species and *Staphylococcus epidermidis*. We dropped all such contaminants from the analysis, as well as any record listed by treating clinicians in the data as a contaminant. We also dropped from the analysis all records where no pathogen was detected, or the patient diagnosis indicated an unspecified bacterium. This assumes that the distribution of pathogens among cases with known aetiology are the same as those with unknown aetiology; in other words that the

probability of detection is the same for every pathogen. This assumption may break down if certain pathogens are more difficult to detect than others, or in cases where a pathogen is irregularly tested for within a laboratory.

For data sources where multiple pathogens were listed per sample ID, we classified these cases according to the following criteria. First, if a case contained more than one of "unspecified bacteria," "virus," "fungus," and another pathogen(s), we chose to drop all these pathogens except the one(s) most likely to be responsible for disease, with the following ranking from most to least likely: 1. Another pathogen(s); 2. Unspecified bacteria; 3. Virus; 4. Fungus. This was to drop co-occurrence profiles that we consider to be uninformative, like a viral infection co-occurring with a fungal infection. After applying this drop, we considered any sample ID that contained more than one pathogen to be polymicrobial. Polymicrobial was treated as a distinct pathogen category in all further analysis, and we were unable to include any AMR burden from polymicrobial infections in our final results, which possibly underestimates the burden of AMR by hiding infections caused by resistant pathogens of interest in the polymicrobial category.

Furthermore, in our approach we chose to assume that the relative prevalences of pathogens in datasets that do not report co-occurrence would be comparable to their mono-pathogenic counterparts in datasets that do report co-occurrence. This assumes that the co-occurrence of pathogens is random and is not correlated for certain pathogens. We did not have sufficient data to fully test the validity of this assumption, given that few datasets report the full universe of pathogens which may co-occur. When selecting pathogens for estimation, we took into account that the set of estimated pathogens for each infectious syndrome is mutually exclusive and collectively exhaustive of all possible aetiologies. Polymicrobial infections were either estimated explicitly or included in the "other" category, making all explicitly estimated individual pathogens mono-pathogenic. Additional factors that were considered can be found in the appendix of Murray et al. (2022).[1]

Section 3.4.3: Dealing with challenges in pathogen distribution appraisal

One of the central challenges of estimating pathogen distributions was that not every data source tested for or reported every possible aetiology of a given infectious syndrome. For example, many literature studies on the aetiologies of meningitis only report on bacterial aetiologies, and some surveillance systems only collect data on certain pathogens of interest. Only certain pathogens are referenced explicitly in the International Classification of Diseases (ICD), limiting which pathogens can be identified from ICD-based data types like MCoD and hospital discharge. Finally, some datasets reported only a subset of the pathogens that we are interested in for a given infectious syndrome, reporting the remaining aetiologies in an aggregate "other" category. These practices have led to inconsistencies in the "other" and "polymicrobial" categories across data sources. Datasets can either over or under-report "other," and datasets that report fewer specific pathogens will automatically report fewer polymicrobial infections.

To address this problem, we maintained a list of data sources that we believe have sufficient testing and reporting to give unbiased estimates of other and polymicrobial for all syndromes, dropping any data on polymicrobial or other that did not come from these data sources. These data sources all had a complete sampling framework (eg, they do not limit the scope of aetologies that they test for) and reported their results without any deliberate aggregation. While we believe this list provided an accurate starting place for the estimation of other and polymicrobial, future work to improve this method would involve a more detailed analysis of sampling framework and reporting categories in each dataset, specific to each infectious syndrome.

There were two major exceptions to this method for handling "other specified pathogens." First, determining the pathogenic aetiology of LRI with microbiology represents challenges that have been well described previously.[8,9] In order to account for this limitation, we utilised a vaccine probe design to inform the *Streptococcus pneumoniae* cause fraction of LRI, consistent with the approach used in the GBD aetiology estimation process.[10,11] In brief, we extracted the vaccine efficacy of the pneumococcal vaccine against all pneumonia from 18 vaccine probe studies with randomised-control trial, before-after, and cohort designs among children and adults. We then calculated the PAF of pneumonia due to *S. pneumoniae* in each study ($Strep\ Base\ PAF$) based on these vaccine efficacies ($VE_{all\ pnuemonia}$), the vaccine efficacy of pneumococcal vaccine against vaccine-type pneumococcal pneumonia as pooled from three studies (two in children and one in adults) (VE_{vtpp}), the percentage of the population covered by the pneumococcal vaccine as modelled in GBD (100% for RCTs) (Cov_{PCV3}),[11] and the percent of serotypes covered

by the vaccine[12] $Cov_{serotype}$ (equation 6.2.6.1). We modelled a global age-specific PAF for *S. pneumoniae* based on these data in the MR-BRT environment and finally adjusted this PAF based on the vaccine coverage in children in every GBD location in 2019 and optimal vaccine efficacy in children ($Strep\ Final\ PAF$) (equation 3.4.3.2). In adults (age 5+), we assumed the effects of vaccination on adults would be primarily indirect from vaccination in children, and included an adjustment factor on the vaccine efficacy to account for this, derived from Grijalva et al.[13]

$$Strep\ Base\ PAF = \frac{VE_{all\ pneumonia}}{VE_{vtpp}Cov_{PCV3}Cov_{serotype}} \tag{3.4.3.1}$$

$$Strep\ Final\ PAF = \frac{Strep\ Base\ PAF\left(1 - Cov_{PCV3}Cov_{serotype}VE_{PCV3\ Optimal}\right)}{1 - (Strep\ Base\ PAF)Cov_{PCV3}Cov_{serotype}VE_{PCV3\ Optimal}} \tag{3.4.3.2}$$

In this vaccine probe analysis, $(1 - Strep\ Final\ PAF)$ is not consistent with the "other" category in our model, since it includes all non--*S. pneumoniae* aetiologies. We retained all of the data from the vaccine probe analysis as two categories, *S. pneumoniae* and "not *S. pneumoniae*" and addressed the inconsistencies between them and our other data using our modelling framework.

The second major exception involves several literature studies on the proportion of neonatal bacterial meningitis caused by *Streptococcus agalactiae* (Group B *Streptococcus;* GBS). We found that these literature studies were important to our estimation of the pathogen distribution of neonatal meningitis, which is distinct from other age groups because of its high proportion of GBS. However, these studies either only reported or were only extracted with two categories, GBS and "other bacterial, not GBS." We retained both these categories and addressed the inconsistencies between them and our other data using our modelling framework.

Section 3.4.4: Age-sex splitting and standardizing measures

We standardised age and sex across all datasets to the following most-detailed groups using the GBD causes of death age-sex splitting algorithm for age:[14] 0–6, 7–27, and 28–364 days, and 1–4, 5–9, 10–14, 15–19, 20–24, 25–29, 30–34, 35–39, 40–44, 45–49, 50–54, 55–59, 60–64, 65–69, 70–74, 75–79, 80–84, 85–89, 90–94, 95+ years; and sex: male and female. This algorithm assumes that age-sex pattern of the death or case rate for a given infectious syndrome or pathogen is inherent to the pathology of the disease and is therefore constant across location and year. Details on how the algorithm was applied can be found in the appendix of Murray et al. (2022).[1]

The input data sources reported a variety of combinations of measures, including some that reported deaths only, some that reported cases only, and some that reported both cases and deaths. In order to standardise these measures to cases, we estimated infectious syndrome- and pathogen-specific CFRs and used these CFRs to convert all deaths-only datasets to cases. For any infectious syndrome or pathogen combination for which we did not have enough data to estimate plausible CFRs, we used a set of all-bacteria CFRs for that infectious syndrome instead. All modelling was done in case space.

Several of our microbial databases came exclusively from ICUs and were therefore heavily biased towards severe illness. In order to mitigate this bias, we dropped all information on cases in ICU-only datasets and recalculated implied cases based on reported deaths and our CFRs. No similar adjustment was made to attempt to account for biases between hospitalised and un-hospitalised populations, although we did account for HAI versus CAI for two infectious syndromes – LRI and thorax infections and UTI – within our modelling framework. The use of hospital-based data to calculate both pathogen-specific case fatality ratios and pathogen distributions biases our estimate of the distribution of pathogens in incident cases towards more severe disease, particularly for less-severe infectious syndromes like lower respiratory infections; adjusting for this bias would improve the accuracy of our non-fatal estimates.

Section 3.4.5: Modelling framework

To model the distribution of pathogens for each infectious syndrome, we developed a method for the multinomial estimation of partial and compositional observations (MEPCO). We assumed that the aetiologies of a given infectious syndrome followed a multinomial distribution. Due to inconsistencies in which pathogens are tested for

and reported by different data sources, each data source contained partial observations of the possible outcomes of the underlying multinomial distribution. Certain data sources like the vaccine probe estimates and the GBS neonatal meningitis studies represent compositional observations, where pathogens like "not *S. pneumoniae"* and "other bacterial, not GBS" represent aggregates of more detailed pathogens.

In order to use both partial and compositional data, we constructed a network model with the dependent variable as the log ratio of cases between different pathogens and estimated over a flexible parameterisation of multinomial parameters using a maximum likelihood approach. Consider a given infectious syndrome with a multinomial distribution of n mutually exclusive, collectively exhaustive aetiologies with probabilities $p = (p_1, \dots, p_n)$, so that each $p_j \in (0,1)$ and $\sum_j p_j = 1$. The likelihood of an observation of $c = (c_1, \dots, c_n)$, where c_j = number of cases of pathogen j in a total sample of N infections ($\sum_j c_j = N$), is:

$$P(c|p) = N! \prod_{j=1}^{n} \frac{p_j^{c_j}}{c_j!}$$

We modelled the probabilities as proportional to a link function with a linear predictor:

$$p_{i,j} \propto \exp\left(x_{i,j}^T \beta_j\right)$$

for observations i, a vector of covariates $x_{i,j}$, and a vector of coefficientsβ_j for each pathogen j. the appendix of Murray et al. (2022)[1] contains a table with the covariates used for infectious syndrome model, which included a typical specification included an intercept term, HAQ Index, a categorical age group dummy for large age bins, and any relevant vaccine coverage proportions by country. However, we did not observe these probabilities directly. Rather, we observed ratios between sums of these probabilities, which reduce to ratios between sums of cases within each study. These observations therefore take the form:

$$y_i = \frac{cases\ of\ pathogen\ A}{cases\ of\ pathogen\ B} = \frac{\sum_{j=1}^{n} w_{i,j}^a \exp\left(x_{i,j}^T \beta_j\right)}{\sum_{j=1}^{n} w_{i,j}^b \exp\left(x_{i,j}^T \beta_j\right)}$$

where $w_{i,j}^a$ is a weight of 0 or 1 that selects the mutually exclusive, collectively exhaustive most-detailed pathogens that make up observed pathogen A, which may be a composite observation. For example, for the "other bacterial, non-GBS" pathogen, $w_{i,j}$ would be 1 for *Staphyloccocus aureus, S. pneumoniae, Haemophilus influenzae, Neisseria meningitidis, Listeria monocytogenes, K. pneumoniae, E. coli*, and other pathogens and 0 for GBS and virus. We dropped all observations where either the numerator or denominator had 0 observed cases in order to make this calculation and a forthcoming log transform possible. This may bias the model towards overestimating less common pathogens.

It is not possible to infer all coefficients β_j from the observations, since they are all relative. However, if we fix all of the coefficients for one pathogen to 0 as a reference group, then we obtain a well-posed inverse problem, as long as there is enough data to estimate the remaining coefficients. Without loss of generality, we assumed $\beta_1 = 0$ for all elements and obtain estimates of the remaining $\beta_2, \dots, \beta_n$ by minimising the sum of the residuals between log-transformed observations y and corresponding log-transformed predictions from equation 3.3.5.4:

$$\min_{\beta_2,\dots,\beta_n} f(\beta) \coloneqq \sum_i \frac{1}{\sigma_i^2} \left[\ln(y_i) - \ln\left(\sum_{j=1}^{n} w_{i,j}^a\, exp\left(x_{i,j}^T \beta_j\right) \right) + \ln\left(\sum_{j=1}^{n} w_{i,j}^b\, exp\left(x_{i,j}^T \beta_j\right) \right) \right]^2 \qquad (3.4.5.4)$$

where σ_i^2 are variances corresponding to the data points. Equation 3.3.5.4 is a nonlinear likelihood minimisation problem that that we optimised using a standard implementation of the Gauss-Newton method.[15] We then re-normalised the optimal coefficients to obtain final predictions of the probabilities of each pathogen:

$$p_{i,j} = \frac{\exp\left(x_{i,j}^{T}\beta_j\right)}{\sum_{\hat{j}} \exp\left(x_{i,\hat{j}}^{T}\beta_{\hat{j}}\right)} \tag{3.4.5.5}$$

To quantify the uncertainty of this estimate, we used asymptotic statistics to obtain the posterior distribution of $(\beta_2, \dots, \beta_n)$. Specifically, using the Gauss-Newton Hessian approximation gave us the asymptotic information matrix for all β_j except for the reference pathogen, allowing us to sample draws of $\beta = (\beta_1 = 0, \beta_2, \dots, \beta_n)$. For each β draw and given feature x, we obtained a corresponding draw of p using equation 3.3.3.5.

Finally, to convert $p_{i,j}$ for a given demographic group i from case space to deaths space, we transformed using our CFR estimate for demographic i:

$$p_{i,j}^{deaths} = \frac{p_{i,j} \times CFR_i}{\sum_{\hat{j}} p_{i,\hat{j}} \times CFR_i} \tag{3.4.5.6}$$

This network regression with covariates framework allowed us to use partial and composite data that reported on one or only a few pathogens, or that reported multiple pathogens aggregated together. Networks, however, can be unstable with sparse data and stable estimates have in some cases required the use of Bayesian priors in these models. In particular, we imposed Gaussian priors with mean 0 and non-zero variance on all coefficients except intercepts, to bias the model away from spurious effects driven by data sparsity. These priors were based on expert opinion and can improved with further empirical validation in the future (appendix of Murray et al.[1]).

Table 3.4.6: Pathogens included in each infectious syndrome model

**Infectious syndrome**	**Pathogens assessed**	**Model covariates**	**Age groups**
Bloodstream infections	*Acinetobacter baumannii*, *Citrobacter* spp., *Enterobacter* spp., *Enterococcus faecalis*, *Enterococcus faecium*, other enterococci, *Escherichia coli*, fungus, group A *Streptococcus*, group B *Streptococcus*, *Klebsiella pneumoniae*, *Neisseria meningitidis*, non-typhoidal *Salmonella*, polymicrobial, *Proteus* spp., *Pseudomonas aeruginosa*, *Salmonella* Typhi, *Serratia* spp., *Staphylococcus aureus*, *Streptococcus pneumoniae*	HAQ Index,[14] age group, age-standardised proportion of intravenous drug use,[23] proportion coverage by PCV3 vaccine,[33] indicator variable for Europe	Neonatal,<br>Post-neonatal–5,<br>5–50,<br>50–70,<br>70+
Infections of bones, joints, and related organs	*Enterococcus faecalis*, *Enterococcus faecium*, other enterococci, *Escherichia coli*, group A *Streptococcus*, group B *Streptococcus*, *Klebsiella pneumoniae*, *Pseudomonas aeruginosa*, *Staphylococcus aureus*	HAQ Index, age group	Under 5,<br>5–50,<br>50–70,<br>70+
Endocarditis and other cardiac infections	See bloodstream infection pathogens	Not explicitly modelled. Pathogen distribution for bloodstream infections is used.	Neonatal,<br>Post-neonatal–5,<br>5–50,<br>50–70,<br>70+
Diarrhoea	Adenovirus, *Aeromonas* spp., Amebiasis, *Campylobacter* spp., *Clostridium difficile*, cryptosporidium, enteropathogenic *Escherichia coli*, enterotoxigenic *Escherichia coli*, non-typhoidal *Salmonella*, norovirus, rotavirus, *Shigella* spp., *Vibrio cholerae*	Not modelled here. GBD diarrhoea aetiology estimates are used.	GBD most detailed age groups
Lower respiratory infections and all related infections in the thorax	*Acinetobacter baumannii*, *Chlamydia* spp., *Enterobacter* spp., *Escherichia coli*, fungus, group B *Streptococcus*, *Haemophilus influenzae*, *Klebsiella pneumoniae*, *Legionella* spp., *Mycoplasma* spp., polymicrobial, *Pseudomonas aeruginosa*, *Staphylococcus aureus*, *Streptococcus pneumoniae*, Influenza viruses, Respiratory syncytial virus, other viruses	HAQ Index, proportion coverage by PCV3 vaccine, proportion coverage by Hib3 vaccine,[33] age group, HAI/CAI	Neonatal,<br>Post-neonatal–5,<br>5–50,<br>50–70,<br>70+
Meningitis and other bacterial	*Escherichia coli*, group B *Streptococcus*, *Haemophilus influenzae*, *Klebsiella pneumoniae*,	HAQ Index, proportion coverage by PCV3 vaccine, proportion coverage	Neonatal,<br>Post-neonatal–5,

central nervous system infections	*Listeria monocytogenes*, *Neisseria meningitidis*, *Staphylococcus aureus*, *Streptococcus pneumoniae*, virus	by Hib3 vaccine, age group, proportion of population covered by ’10-’15 MenAfriVac rollout[1,34]	5-50, 50-70, 70+
Peritoneal and intra-abdominal infections	*Citrobacter* spp., *Enterobacter* spp., *Enterococcus faecalis*, *Enterococcus faecium*, *Escherichia coli*, *Klebsiella pneumoniae*, other *Klebsiella* species, *Proteus* spp., *Pseudomonas aeruginosa*, *Serratia* spp., *Staphylococcus aureus*	HAQ Index, age group	Under 5, 5–50, 50–70, 70+
Bacterial infections of the skin and subcutaneous systems	*Acinetobacter baumannii*, *Enterobacter* spp., *Enterococcus faecalis*, other enterococci, *Escherichia coli*, group A *Streptococcus*, group B *Streptococcus*, *Klebsiella pneumoniae*, *Proteus* spp., *Pseudomonas aeruginosa*, *Staphylococcus aureus*	HAQ Index, age group	Under 5, 5–50, 50–70, 70+
Urinary tract infections and pyelonephritis	*Acinetobacter baumannii*, *Citrobacter* spp., *Enterobacter* spp., *Enterococcus faecalis*, *Enterococcus faecium*, other enterococci, *Escherichia coli*, group B *Streptococcus*, *Klebsiella pneumoniae*, *Morganella* spp., *Proteus* spp., *Providencia* spp., *Pseudomonas aeruginosa*, *Serratia* spp., *Staphylococcus aureus*	HAQ Index, age group, HAI/CAI	Under 5, 5–50, 50–70, 70+

Group A *Streptococcus* = *Streptococcus pyogenes.* Group B *Streptococcus* = *Streptococcus agalactiae.* HAQ Index = Healthcare Access and Quality Index. HAI/CAI = hospital-acquired infection/community-acquired infection. * Enterotoxigenic *Escherichia coli* (ETEC) and Enteropathogenic *Escherichia col*i (EPEC) are only reported for the diarrhea syndrome.

Section 3.4.7: Exceptions and special handling

There were several notable exceptions and special handling decisions made for each individual pathogen distribution model, which we hope to address with more sustainable approaches in our future work. For example, for cardiac infections, we used the pathogen distribution for bloodstream infections rather than estimating specific distributions for these syndromes, due to a lack of complete literature reviews on the aetiologies and case-fatality rates of these syndromes. We consider this to be a serious limitation of our methodology, but do not anticipate that is seriously impactful on our final estimates.

In diarrhoea patients, cultures of specimens taken from the gastrointestinal tract, bowels, rectum, or stool are almost always affected by contaminants or pathogens that are not the cause of diarrhoea. For this reason, we believe that our input data and modelling framework are not able to accurately capture the aetiologies of diarrhoea. We chose to use GBD estimates of the aetiologies of diarrhoea in deaths instead of running our own model.[13] Nonetheless, a major limitation of using such approach is that the GBD diarrhoea aetiology estimates are population attributable fractions (PAFs) for each pathogen. These PAFs may add to greater than 1 and the authors made no attempt to quantify the extent of co-occurrence of pathogens; the latter is inconsistent with the pathogen distribution estimation method used in our study, which quantifies polymicrobial infections and estimates all pathogens as mono-infections. Hence, in order to avoid duplication of cases in our framework, we had to make some assumptions about the co-occurrence of pathogens in diarrhoea (details provided in the appendix of Murray et al.[1]).

Certain skin and subcutaneous samples are easily affected by contaminants, colonization, and other pathogens that are not the cause of infection. For this reason, we considered microbial data and mortality surveillance to be too difficult to extract meaningful aetiology information from, and instead used only ICD-coded databases (multiple cause of death, hospital discharge, and linkage data) and literature studies as inputs into our model of the pathogen distribution of skin infections.

We dropped all data on *S. pneumoniae* for community-acquired LRI and thorax infections in non-neonatal age groups except our estimates from the vaccine probe analysis. Because dedicated anaerobic cultures were not routinely performed for peritoneal samples, we dropped all anaerobes observed in the data for and excluded anaerobes as an etiology of intra-abdominal infections. Moreover, due to the unique pattern of meningitis in neonates, particularly the high prevalence of GBS, we modeled neonatal and adult central nervous syndrome infections separately.

For three infectious syndromes, we did not run a pathogen distribution model – these are “Typhoid, paratyphoid, and invasive non-typhoidal *Salmonella*”, “Tuberculosis” and “Gonorrhoea and chlamydia” infectious syndromes. They are all caused by distinct pathogens whose individual burdens are already estimated in GBD as separate causes of

death. Therefore, for these syndromes, we simply used GBD estimates. MTB- or HIV-associated opportunistic infections were not included as part of the infectious syndrome aetiology because they are classified as MTB and HIV cases according to the GBD methodology.

Section 3.4.8: Model validation

To assess model validity, we calculated the root mean square error (RMSE) and coefficient of determination (R^2) for each pathogen distribution model in proportion space for both in-sample and out-of-sample predictions. Proportions were predicted for each observation using the specific denominator observed from that study. For example, if a given study reported on only *E. coli* and *S. pneumoniae*, the predictions for model validation for this study were calculated as proportions of the total for *E. coli* and *S. pneumoniae.* In order to calculate out-of-sample fit, we perform non-exhaustive cross-validation, with each round of the validation holding out 1 country of data at a time. This leave-one-country-out approach simulates the prediction task of estimating the pathogen distribution of a country for which we have no data.

R^2 ranges from 0.784 to 0.867 in-sample and from 0.755 to 0.837 out of sample, indicating good model fit with only modest losses when data are moved out of sample. RMSE ranges from 0.129 to 0.149 in-sample and from 0.141 to 0.159 out of sample. Given that the data are expected to vary from the model predictions according to the observation-level variance, and the fact that the RMSEs are relatively consistent between in-sample and out-of-sample, these RMSEs are reasonable. Overall, these metrics show that these models have good fit and good out-of-sample predictive ability.

Table 3.4.8.1: In-sample and out-of-sample validation metrics for pathogen distribution models (GLOBAL)

Infectious syndrome	Model type	R^2		RMSE	
		In sample	Out of sample	In sample	Out of sample
Bacterial infections of the skin and subcutaneous systems		0.808	0.771	0.129	0.141
Bloodstream infections		0.822	0.785	0.128	0.141
Infections of bones, joints, and related organs		0.858	0.837	0.141	0.151
Lower respiratory infections and all related infections in the thorax		0.810	0.780	0.142	0.153
Meningitis and other bacterial central nervous system infections	Neonatal	0.858	0.803	0.134	0.158
	Non-neonatal	0.867	0.822	0.129	0.150
Peritoneal and intra-abdominal infections		0.815	0.812	0.147	0.148
Urinary tract infections and pyelonephritis		0.784	0.755	0.149	0.159

Out of sample metrics calculated using leave-one-country-out cross validation

Section 4: Pathogen-specific ratios for GBD pathogen adjustment

To make our estimates of burden comparable between all pathogens included in the paper, we developed pathogen-specific ratios that incorporated the burden of immediate and intermediate causes of death from pathogens modeled exclusively by the GBD. We used these ratios to adjust both deaths and years of life lost (YLLs). Adjusted YLLs were then combined with years lived with disability (YLDs) to generate adjusted disability-adjusted life years (DALYs). We generated ratios for the following pathogens:

- Hepatitis A
- Hepatitis B
- Hepatitis E
- Ascariasis
- Human papillomavirus
- Chagas disease
- Cystic echinococcosis

- Cysticercosis
- Dengue
- Diphtheria
- HIV/AIDS
- Malaria
- Measles
- *Neisseria gonorrhoeae*
- Other neglected tropical diseases
- Rabies
- Schistosomiasis
- Syphilis
- Tetanus
- Tuberculosis
- Varicella and herpes zoster
- Visceral leishmaniasis
- *Bordetella* species (Whooping Cough)
- Yellow fever

Section 4.1: Generating the ratios

To create these ratios, we used multiple cause of death (MCOD), hospital, linkage, and MITS data (Table 4.1.1) to determine the pathogen-specific fraction of deaths coming from immediate and intermediate causes of death, also known as the cause of death chain. Ratios were made only for pathogens with at least 200 recorded deaths in our dataset. The median number of deaths per pathogen in our data was 5,345 for all ages, 4,609 for the age group 5 plus, and 981 for under 5. Next, we divided the total number of deaths where the pathogen was the underlying cause of death (α) by the total number of deaths where the pathogen was diagnosed anywhere in the cause of death chain (β) (Equation 4.1.1). A ratio of 1 indicated that no fatal burden was lost by considering only the underlying cause of death estimates provided by the GBD.

$$\frac{\alpha}{\beta} \text{(Equation 4.1.1)}$$

We generated the ratios for the following age groups: under 5, over 5, and all ages. The means of a binomial distribution were used for the point estimates and the 95% UIs were delineated using the 2.5th and 97.5th percentiles of 1,000 draws. Uncertainty is attributable to sample size variability between data sources and data availability. The binomial distribution was defined by n, the number of times the cause appeared anywhere in the cause of death chain, including the underlying cause of death, and p, the probability of the cause being the underlying cause of death (Equation 4.1.2).

$$\frac{1}{\frac{Bin(n,p)}{n}} \text{(Equation 4.1.2)}$$

Table 4.1.1. Input data to the pathogen-specific ratios

Country	Source	Years	Year Range	Deaths
**Austria**	Hospital	18	2001-2018	460,840

**Bangladesh**	MITS (from CHAMPS)	6	2017-2022	105
**Brazil**	Hospital	6	2015-2020	964,447
	MCOD	23	1999-2021	20,980,227
**Canada**	Hospital	16	1994-2009	45,191
**Colombia**	MCOD	24	1998-2021	4,711,423
**Ethiopia**	MITS (from CHAMPS)	3	2019-2021	71
**Georgia**	Hospital	7	2014-2020	34,612
**India**	Hospital	4	2014-2017	13,371
**Italy**	Hospital	17	2005-2021	3,695,034
	Linkage	16	2003-2018	112,371
	MCOD	18	2003-2020	10,605,540
**Kenya**	MITS (from CHAMPS)	6	2017-2022	267
**Kyrgyzstan**	Hospital	1	2012	9
**Libya**	Hospital	2	2019-2020	439
**Mali**	MITS (from CHAMPS)	5	2017-2021	93
**Mexico**	Hospital	21	2000-2020	833,344
	MCOD	8	2009-2016	4,324,274
**Mongolia**	Hospital	2	2019-2020	2
	MCOD	3	2018-2020	13,192
**Mozambique**	MITS (from CHAMPS)	6	2017-2022	331
**New Zealand**	Hospital	10	2011-2020	116,643
	Linkage	11	2000-2010	144,515
**Pakistan**	Hospital	3	2017-2019	4,214
**Philippines**	Claims	1	2016	75,664
**Sierra Leone**	MITS (from CHAMPS)	4	2019-2022	287
**South Africa**	MCOD	20	1997-2016	4,687,023
	MITS (from CHAMPS)	6	2017-2022	567
**Taiwan (Province of China)**	MCOD	10	2008-2017	1,185,682
**United Arab Emirates**	MCOD	5	2014-2018	64,380
**United States of America**	Hospital	31	1980-2010	7,940,360
	MCOD	41	1980-2020	77,287,402

Recorded deaths in Table 4.1.1. meet two criteria:

1. At least two unique causes of death and/or diagnoses are associated with the death records
2. The ICD-codes used to describe the causes of death and/or diagnoses have at least 2 digits of detailž

Here, we would also like to clarify the steps of our modelling procedure in which we relied on data from the fewest number of countries. These were 1) to estimate the proportion of each underlying cause of death associated with sepsis and specific infectious syndromes, and 2) the ratio indicating how commonly a diagnosed infectious cause

was implicated as an intermediate vs. underlying cause of death by the attending clinician. With respect to #1, we use data from 16 countries, while for #2, we used data from 24 countries (including Mongolia, Pakistan, the Philippines, and the United Arab Emirates).

Section 4.2: Exceptions

The following pathogens were not adjusted due to being recorded in fewer than 200 death records in our data:

- African trypanosomiasis
- *Salmonella* Paratyphi (Paratyphoid fever)
- Zika Virus

The following pathogens were adjusted using all age scalars for the under 5 age group due to having fewer than 200 death records in the under 5 age group:

- Chagas disease
- Cystic echinococcosis
- Cysticercosis
- Diphtheria
- Hepatitis B
- *Neisseria gonorrhoaea*
- Rabies
- Schistosomiasis
- Yellow fever

Acute hepatitis E was not adjusted in under 5 due to the rarity of this pathogen in this age group and cervical cancer was not adjusted for under 5 due to the realistic age restrictions associated with this cause of death.

Section 4.3: Ratios

Table 4.3.1. pathogen-specific ratios

**Pathogen**	**Age**	**Scalar (95% UI)**
Yellow fever	Under 5	1.04 (1.02 - 1.06)
	5 plus	1.04 (1.02 - 1.06)
	All Ages	1.04 (1.02 - 1.06)
Cystic echinococcosis	Under 5	1.65 (1.55 - 1.75)
	5 plus	1.65 (1.55 - 1.75)
	All Ages	1.65 (1.55 - 1.75)
Hepatitis E	Under 5	1.0 (1.0 - 1.0)
	5 plus	1.74 (1.59 - 1.95)
	All Ages	1.75 (1.57 - 1.95)
Rabies	Under 5	1.15 (1.1 - 1.2)
	5 plus	1.14 (1.1 - 1.19)
	All Ages	1.15 (1.1 - 1.2)
Cysticercosis	Under 5	1.27 (1.25 - 1.29)
	5 plus	1.27 (1.25 - 1.28)
	All Ages	1.27 (1.25 - 1.29)
Human papillomavirus	5 plus	1.07 (1.07 - 1.08)
	All Ages	1.07 (1.07 - 1.08)
Schistosomiasis	Under 5	1.08 (1.07 - 1.09)

	5 plus	1.08 (1.07 - 1.08)
	All Ages	1.08 (1.07 - 1.09)
Chagas disease	Under 5	1.06 (1.05 - 1.06)
	5 plus	1.06 (1.05 - 1.06)
	All Ages	1.06 (1.05 - 1.06)
Neisseria gonorrhoeae	Under 5	1.67 (1.55 - 1.81)
	5 plus	1.66 (1.54 - 1.79)
	All Ages	1.67 (1.55 - 1.81)
Dengue	Under 5	1.03 (1.02 - 1.04)
	5 plus	1.09 (1.09 - 1.1)
	All Ages	1.09 (1.08 - 1.09)
Tetanus	Under 5	1.15 (1.1 - 1.21)
	5 plus	1.07 (1.06 - 1.08)
	All Ages	1.08 (1.07 - 1.08)
Hepatitis A	Under 5	1.07 (1.05 - 1.1)
	5 plus	1.63 (1.6 - 1.65)
	All Ages	1.58 (1.56 - 1.61)
Diphtheria	Under 5	2.29 (2.03 - 2.63)
	5 plus	2.68 (2.26 - 3.21)
	All Ages	2.29 (2.03 - 2.63)
Visceral leishmaniasis	Under 5	1.03 (1.02 - 1.04)
	5 plus	1.1 (1.09 - 1.12)
	All Ages	1.08 (1.08 - 1.09)
Hepatitis B	Under 5	1.32 (1.31 - 1.34)
	5 plus	1.32 (1.31 - 1.33)
	All Ages	1.32 (1.31 - 1.34)
Malaria	Under 5	1.18 (1.14 - 1.22)
	5 plus	1.13 (1.11 - 1.14)
	All Ages	1.13 (1.12 - 1.15)
Measles	Under 5	1.31 (1.24 - 1.38)
	5 plus	1.59 (1.47 - 1.72)
	All Ages	1.43 (1.37 - 1.5)
Other neglected tropical diseases	Under 5	1.28 (1.22 - 1.35)
	5 plus	1.33 (1.31 - 1.34)
	All Ages	1.32 (1.31 - 1.34)
Bordetella species (Whooping Cough)	5 plus	1.89 (1.68 - 2.13)
	Under 5	1.08 (1.06 - 1.09)
	All Ages	1.13 (1.11 - 1.15)
Ascariasis	Under 5	1.21 (1.17 - 1.25)
	5 plus	1.36 (1.29 - 1.43)
	All Ages	1.26 (1.23 - 1.3)

Varicella and herpes zoster	Under 5	1.17 (1.15 - 1.18)
	5 plus	2.45 (2.42 - 2.48)
	All Ages	2.26 (2.24 - 2.29)
Syphilis	Under 5	1.28 (1.26 - 1.3)
	5 plus	2.53 (2.48 - 2.59)
	All Ages	1.98 (1.95 - 2.0)
HIV/AIDS	Under 5	1.14 (1.13 - 1.14)
	5 plus	1.1 (1.1 - 1.1)
	All Ages	1.1 (1.1 - 1.1)
Tuberculosis	Under 5	1.39 (1.37 - 1.4)
	5 plus	1.43 (1.42 - 1.43)
	All Ages	1.43 (1.42 - 1.43)

Section 5: *Helicobacter pylori* burden estimation:

The burden for *Helicobacter pylori* was estimated based on GBD figures for stomach cancer, which were adjusted to represent total non-cardia stomach cancer, a form of stomach cancer for which *H. pylori* is thought to be the dominant cause. Fractions of stomach cancers that were non-cardia as compared to cardia were obtained from de Martel et al. 2020 which documented that 80.9% and 86.7% of gastric cancers for males and females, respectively, were non-cardia neoplasms.[16] Based on the estimated fraction of gastric non-cardia cancers attributable to *H. pylori* from the same research (89%, 95%CI: 79-94), we then attributed a proportion of non-cardia-adjusted GBD stomach cancers to *H. pylori*. We incorporated the uncertainty for the estimated *H. pylori* attributable fraction by approximating a beta distribution with the same mean and 95% confidence bounds as those found in de Martel et al. 2020, sampling 1,000 draws from that distribution, and ultimately multiplying those draws with GBD estimates. Limitations of this approach include the assumption that the proportion of non-cardia gastric cancer is consistent across the world, and the lack of attribution of burden of cardia cancers and non-Hodgin gastric lymphomas to *H. pylori*.

Section 6: Supplementary tables

Supplementary Table 1: Death rate per 100 000 population associated with specific pathogens in each GBD super-region for all ages and those under 5 years of age, 2019.

	Central Europe, Eastern Europe, and Central Asia		High-income		Latin America and Caribbean		North Africa and Middle East		South Asia		Southeast Asia, East Asia, and Oceania		Sub-Saharan Africa	
	all ages	under 5	all ages	under 5	all ages	under 5	all ages	under 5	all ages	under 5	all ages	under 5	all ages	under 5
Acinetobacter baumannii	4.12 (2.37-6.66)	2.23 (1.41-3.43)	3.95 (2.36-6.24)	0.6 (0.35-0.94)	5.43 (3.36-8.16)	5.17 (3.32-7.67)	4.71 (2.84-7.17)	7.11 (4.33-10.96)	8.4 (5.39-12.42)	16.09 (9.72-24.7)	8.19 (4.88-12.58)	4.65 (3.06-6.84)	7.81 (5.58-10.81)	20.15 (12.83-30.71)
**Adenovirus**	0.01 (0.01-0.03)	0.15 (0.07-0.32)	0.05 (0.03-0.08)	0.05 (0.02-0.09)	0.13 (0.07-0.23)	1.21 (0.6-2.33)	0.31 (0.13-0.68)	3.02 (1.19-6.66)	1.29 (0.73-2.16)	6.22 (3.04-11.84)	0.11 (0.07-0.18)	0.99 (0.48-1.89)	4.53 (2.45-7.5)	25.4 (12.76-44.45)
***Aeromonas* spp.**	0.01 (0-0.02)	0.06 (0.02-0.14)	0.01 (0-0.01)	0 (0-0)	0.03 (0.01-0.04)	0.15 (0.05-0.36)	0.08 (0.03-0.18)	0.71 (0.21-1.65)	0.43 (0.21-0.78)	2.15 (0.89-4.01)	0.02 (0.01-0.04)	0.08 (0.03-0.17)	1.13 (0.48-2.18)	5.58 (2.07-11.48)
**African trypanosomiasis**	0 (0-0)	0 (0-0)	0 (0-0)	0 (0-0)	0 (0-0)	0 (0-0)	0 (0-0)	0 (0-0)	0 (0-0)	0 (0-0)	0 (0-0)	0 (0-0)	0.13 (0.06-0.24)	0.03 (0.01-0.14)
**Ascariasis**	0 (0-0)	0 (0-0.01)	0 (0-0)	0 (0-0)	0.01 (0.01-0.02)	0.05 (0.03-0.08)	0.01 (0.01-0.02)	0.06 (0.04-0.09)	0.02 (0.02-0.02)	0.08 (0.05-0.1)	0.01 (0.01-0.01)	0.04 (0.03-0.05)	0.18 (0.13-0.23)	0.89 (0.62-1.23)
***Bordetella* species (Whooping Cough)**	0.09 (0.03-0.19)	1.12 (0.4-2.48)	0.01 (0-0.01)	0.11 (0.07-0.19)	0.39 (0.16-0.77)	4.09 (1.74-8.1)	1.39 (0.52-2.99)	12.12 (4.48-26.36)	1.66 (0.42-3.82)	15.39 (3.9-35.15)	0.54 (0.22-1.08)	7.02 (2.9-14.1)	7.41 (3-14.75)	43.08 (17.54-86.63)
***Campylobacter* spp.**	0.08 (0.02-0.18)	0.42 (0.14-0.96)	0.17 (0.03-0.43)	0.02 (0.01-0.04)	0.39 (0.13-0.81)	1.53 (0.59-3.19)	0.13 (0.04-0.3)	0.98 (0.29-2.47)	3.54 (1.03-8.54)	9.85 (4.25-18.51)	0.29 (0.07-0.74)	0.9 (0.33-2.02)	4.38 (1.52-9.02)	15.82 (5.98-33.05)
**Chagas disease**	0 (0-0)	0 (0-0)	0.08 (0.05-0.28)	0 (0-0)	1.56 (0.84-2.61)	0 (0-0.01)	0 (0-0)	0 (0-0)	0 (0-0)	0 (0-0)	0 (0-0)	0 (0-0)	0 (0-0)	0 (0-0)
***Chlamydia* spp.**	0.59 (0.46-0.77)	1.44 (1.11-1.88)	0.55 (0.44-0.67)	0.12 (0.07-0.2)	0.85 (0.68-1.06)	2.03 (1.37-2.88)	0.77 (0.58-1.01)	4.12 (2.89-5.65)	1.8 (1.39-2.27)	9.63 (7.03-12.87)	0.7 (0.53-0.95)	2 (1.46-2.61)	3.28 (2.62-4.09)	15.09 (11.49-19.81)
***Citrobacter* spp.**	0.89 (0.48-1.56)	0.43 (0.22-0.76)	0.56 (0.37-0.81)	0.13 (0.07-0.21)	0.63 (0.41-0.91)	0.96 (0.6-1.48)	0.49 (0.28-0.76)	0.96 (0.54-1.55)	0.8 (0.48-1.24)	2.08 (1.21-3.38)	0.74 (0.43-1.17)	0.87 (0.53-1.33)	0.69 (0.43-1.05)	2.38 (1.4-3.8)
Clostridioides difficile	0.24 (0.13-0.47)	0.15 (0.09-0.24)	1.95 (1.55-2.43)	0.2 (0.16-0.26)	0.27 (0.18-0.38)	1.06 (0.56-1.76)	0.07 (0.04-0.12)	0.11 (0.05-0.2)	0.08 (0.03-0.18)	0.1 (0.04-0.2)	0.27 (0.14-0.5)	0.29 (0.17-0.46)	0.15 (0.08-0.28)	0.18 (0.08-0.36)
***Cryptosporidium* spp.**	0.05 (0.01-0.17)	0.31 (0.05-1.01)	0.07 (0.01-0.25)	0.01 (0-0.03)	0.14 (0.02-0.48)	0.88 (0.14-2.9)	0.35 (0.06-1.01)	3.21 (0.54-9.2)	2.34 (0.34-8.12)	4.64 (0.81-13.61)	0.09 (0.01-0.35)	0.35 (0.05-1.15)	5.82 (1.4-14.42)	27.94 (6.28-63.83)

**Cystic echinococcosis**	0.02 (0.01-0.04)	0 (0-0.01)	0.01 (0-0.01)	0 (0-0)	0 (0-0.01)	0 (0-0)	0.08 (0.06-0.1)	0.03 (0.01-0.06)	0.04 (0.03-0.06)	0.02 (0-0.04)	0.01 (0.01-0.01)	0 (0-0.01)	0.05 (0.03-0.07)	0.09 (0.03-0.16)
**Cysticercosis**	0 (0-0)	0 (0-0)	0 (0-0)	0 (0-0)	0.07 (0.05-0.1)	0 (0-0.01)	0 (0-0)	0 (0-0)	0 (0-0.01)	0 (0-0)	0 (0-0)	0 (0-0)	0.07 (0.05-0.1)	0.01 (0-0.03)
**Dengue**	0 (0-0)	0 (0-0)	0 (0-0)	0 (0-0)	0.25 (0.12-0.29)	0.46 (0.32-0.57)	0.01 (0.01-0.02)	0.04 (0.04-0.05)	1.25 (0.24-1.61)	1.16 (0.22-1.85)	0.68 (0.24-0.87)	3.33 (0.79-4.87)	0.01 (0-0.01)	0.02 (0-0.05)
**Diphtheria**	0 (0-0.01)	0.01 (0-0.01)	0 (0-0)	0 (0-0)	0.01 (0-0.01)	0.03 (0.02-0.05)	0.02 (0.02-0.03)	0.11 (0.05-0.2)	0.02 (0.02-0.03)	0.11 (0.07-0.16)	0.01 (0.01-0.01)	0.05 (0.03-0.07)	0.88 (0.58-1.3)	4.85 (2.98-7.46)
**Ebola**	0 (0-0)	0 (0-0)	0 (0-0)	0 (0-0)	0 (0-0)	0 (0-0)	0 (0-0)	0 (0-0)	0 (0-0)	0 (0-0)	0 (0-0)	0 (0-0)	0.34 (0.28-0.4)	0.2 (0.16-0.23)
Entamoeba histolytica	0.01 (0-0.04)	0.14 (0.03-0.37)	0 (0-0.01)	0.01 (0-0.01)	0.14 (0.04-0.32)	1.23 (0.34-2.9)	0.21 (0.05-0.55)	1.95 (0.47-5.17)	0.75 (0.23-1.84)	2.82 (0.74-6.98)	0.02 (0.01-0.04)	0.12 (0.03-0.32)	1.28 (0.37-2.95)	5.58 (1.4-14.37)
***Enterobacter* spp.**	3.7 (2.35-5.42)	2 (1.36-2.97)	3.57 (2.48-4.96)	0.66 (0.41-1.03)	3.79 (2.58-5.32)	4.79 (3.16-6.96)	3.02 (1.89-4.56)	5.26 (3.34-7.83)	4.28 (2.77-6.21)	11.99 (8.02-17.64)	5.29 (3.23-8.19)	4.66 (3.11-6.75)	3.69 (2.66-5.11)	11.12 (7.91-15.51)
Enterococcus faecalis	4.77 (2.83-7.42)	1 (0.65-1.53)	4.07 (2.53-6.41)	0.4 (0.25-0.63)	3.24 (2.07-4.85)	2.44 (1.7-3.46)	1.92 (1.1-3.06)	2.01 (1.24-3.12)	2.61 (1.61-3.93)	4.15 (2.62-6.32)	2.25 (1.3-3.49)	1.19 (0.82-1.73)	2.76 (1.77-3.95)	6.71 (4.62-9.67)
Enterococcus faecium	4.85 (2.98-7.38)	0.64 (0.41-1)	4.61 (2.93-6.83)	0.37 (0.22-0.58)	3.32 (2.09-4.9)	1.68 (1.13-2.49)	2.08 (1.22-3.34)	1.26 (0.74-2)	2.22 (1.32-3.5)	1.85 (1.16-2.89)	2.53 (1.51-4.01)	0.76 (0.5-1.13)	2.06 (1.24-3.26)	3.13 (2.07-4.67)
**Enteropathogenic *Escherichia coli***	0 (0-0.01)	0.05 (0.02-0.09)	0 (0-0.01)	0 (0-0.01)	0.01 (0.01-0.02)	0.11 (0.05-0.21)	0.08 (0.04-0.16)	0.79 (0.32-1.6)	0.23 (0.11-0.41)	1.23 (0.53-2.41)	0.03 (0.02-0.06)	0.29 (0.12-0.58)	0.98 (0.47-1.83)	5.2 (2.31-10.25)
**Enterotoxigenic *Escherichia coli***	0.03 (0.01-0.06)	0.17 (0.07-0.38)	0.07 (0.03-0.14)	0.02 (0.01-0.04)	0.03 (0.01-0.06)	0.15 (0.06-0.33)	0.15 (0.06-0.32)	1.16 (0.4-2.77)	1.49 (0.59-3.19)	2.55 (0.99-5.39)	0.1 (0.04-0.2)	0.35 (0.12-0.78)	0.65 (0.27-1.32)	2.94 (1.11-6.72)
Escherichia coli	20.34 (12.99-30.21)	5.02 (3.82-6.66)	19.76 (13.78-27.49)	1.41 (0.9-2.1)	12.97 (9.45-17.37)	8.03 (5.55-11.03)	7.24 (4.81-10.41)	11.05 (7.93-15.13)	11.35 (8.43-15.08)	20.83 (15.39-27.62)	7.99 (5.35-11.48)	5.47 (4.14-7.13)	13.18 (10.34-16.94)	38.78 (29.07-52.19)
**Fungi**	2.01 (1.26-3.13)	2.42 (1.37-3.99)	1.37 (1.04-1.85)	0.45 (0.25-0.76)	2.81 (1.95-3.91)	8.61 (5.17-13.59)	2.55 (1.59-3.82)	11.27 (5.91-18.9)	5.6 (3.43-8.59)	27.86 (14.71-47.3)	1.98 (1.34-2.86)	5.39 (3.27-8.51)	12 (7.58-17.94)	57.69 (34.26-90.09)
**Group A *Streptococcus***	3.42 (1.71-6.39)	1.85 (1.21-2.8)	3.03 (1.68-5.34)	0.84 (0.52-1.28)	2.88 (1.72-4.94)	3.82 (2.6-5.39)	1.75 (0.88-3.31)	3.14 (1.92-4.75)	2.42 (1.26-4.37)	4.42 (2.92-6.68)	2.23 (1.04-4.4)	1.73 (1.17-2.5)	2.96 (1.77-4.89)	7.77 (5.24-11.24)
**Group B *Streptococcus***	2.88 (1.86-4.35)	3.09 (2.28-4.1)	3.44 (2.42-4.85)	0.85 (0.55-1.27)	2.96 (2.09-4.13)	5.74 (3.89-7.95)	2.15 (1.43-3.11)	7.55 (5.04-10.53)	3.12 (2.25-4.31)	15.1 (10.83-20.1)	2.42 (1.56-3.59)	3.98 (2.9-5.29)	5.6 (4.33-7.24)	24 (18.16-31.63)
**HIV/AIDS**	7.29 (7.2-7.39)	1.8 (1.76-1.84)	1.36 (1.35-1.37)	0.16 (0.15-0.16)	7.88 (7.42-8.7)	4.07 (2.83-6.86)	1.71 (1-3.37)	1.24 (0.38-4.86)	3.18 (2.57-5.05)	1.78 (0.96-5.43)	4.07 (3.53-4.74)	2.64 (1.81-3.84)	65.31 (57.31-78.21)	27.6 (21.17-35.6)

Haemophilus influenzae	0.88 (0.72-1.11)	1.81 (1.45-2.26)	1.01 (0.85-1.21)	0.17 (0.13-0.23)	0.99 (0.8-1.22)	2.15 (1.57-2.85)	0.76 (0.6-0.98)	3.49 (2.61-4.65)	1.34 (1.07-1.66)	6.98 (5.11-9.1)	0.8 (0.62-1.01)	2.21 (1.74-2.76)	3.09 (2.45-3.9)	14.47 (10.92-18.97)
Helicobacter pylori	13.6 (11.81-15.18)	0 (0-0)	11.42 (9.65-12.69)	0 (0-0)	8.45 (7.21-9.72)	0 (0-0)	4.81 (4.05-5.48)	0 (0-0)	4.09 (3.41-4.78)	0 (0-0)	16.07 (13.15-18.91)	0 (0-0)	2.45 (2.07-2.83)	0 (0-0)
**Hepatitis A**	0.03 (0.02-0.04)	0.07 (0.03-0.11)	0.02 (0.02-0.03)	0.01 (0-0.01)	0.08 (0.06-0.1)	0.11 (0.08-0.15)	0.55 (0.2-1.13)	0.69 (0.24-1.36)	2.25 (1.48-3.31)	4.33 (2.42-6.93)	0.22 (0.11-0.37)	0.05 (0.04-0.07)	0.92 (0.52-1.61)	0.39 (0.18-0.78)
**Hepatitis B**	8.57 (7.05-10.39)	0.24 (0.18-0.33)	4.51 (3.9-5.21)	0.01 (0.01-0.02)	2.73 (2.34-3.18)	0.09 (0.06-0.12)	7.95 (5.87-10.5)	0.43 (0.17-0.92)	10.05 (8.36-12.06)	1.91 (0.65-3.48)	14.29 (12.12-16.5)	0.14 (0.1-0.17)	8.75 (7.09-10.7)	0.71 (0.45-1.14)
**Hepatitis C**	7.91 (6.55-9.6)	0.01 (0.01-0.02)	11.4 (10.1-12.58)	0 (0-0)	5.99 (5.1-7.03)	0.02 (0.01-0.03)	9.7 (7.06-12.24)	0.03 (0.02-0.07)	5.2 (4.36-6.14)	0.3 (0.09-0.55)	6.99 (6.02-8.02)	0.01 (0.01-0.02)	4.34 (3.53-5.27)	0.08 (0.03-0.16)
**Hepatitis E**	0.01 (0-0.01)	0.02 (0-0.04)	0 (0-0.01)	0 (0-0)	0.01 (0.01-0.01)	0.01 (0.01-0.02)	0.02 (0.01-0.06)	0.02 (0.01-0.05)	0.09 (0.05-0.17)	0.21 (0.1-0.39)	0.03 (0.02-0.05)	0.01 (0.01-0.01)	0.04 (0.02-0.08)	0.03 (0.01-0.06)
**Human papillomavirus**	5.23 (4.6-5.92)	0 (0-0)	3.06 (2.65-3.27)	0 (0-0)	6.1 (5.37-7.03)	0 (0-0)	1.24 (0.96-1.47)	0 (0-0)	3.17 (2.55-4.17)	0 (0-0)	4.07 (2.94-4.87)	0 (0-0)	5.39 (4.17-6.57)	0 (0-0)
**Influenza virus**	3.49 (3.14-3.87)	6.42 (5.17-8.04)	7.94 (6.7-8.75)	0.56 (0.42-0.76)	5.92 (5.17-6.62)	7.63 (5.55-10.05)	3.14 (2.67-3.67)	11.18 (8.36-14.53)	5.19 (4.27-6.22)	22.23 (16.64-28.99)	3.39 (2.9-4.02)	6.17 (4.84-7.7)	10.27 (8.58-12.47)	39.73 (30.19-51.78)
**Invasive Non-typhoidal *Salmonella* (iNTS)**	0.9 (0.51-1.48)	0.97 (0.6-1.53)	0.27 (0.12-0.51)	0.13 (0.08-0.19)	0.74 (0.43-1.21)	3.06 (1.85-4.87)	1.15 (0.74-1.73)	5.41 (3.08-9.38)	2.71 (1.55-4.55)	9.5 (6.13-14.15)	0.86 (0.54-1.31)	2.91 (1.86-4.46)	12 (7.11-18.59)	53.89 (32.01-81.8)
Klebsiella pneumoniae	11.07 (7.4-15.87)	7.26 (5.51-9.66)	10.25 (7.38-14)	1.56 (1.01-2.31)	10.69 (7.67-14.55)	14.25 (9.92-19.75)	7.08 (4.84-10.15)	17.44 (12.24-24.39)	11.14 (8.03-15.01)	33.73 (24.45-45.75)	7.23 (4.99-10.19)	9.18 (6.9-11.94)	17.67 (13.76-22.63)	63.85 (48.14-82.82)
***Legionella* spp.**	1.14 (0.98-1.34)	0.94 (0.64-1.36)	2.12 (1.81-2.43)	0.14 (0.07-0.25)	1.15 (1-1.34)	1.2 (0.7-1.94)	0.72 (0.58-0.91)	1.95 (1.11-3.18)	1.07 (0.83-1.4)	4.33 (2.52-7.03)	0.97 (0.79-1.21)	1.07 (0.66-1.65)	1.46 (1.09-1.98)	5.12 (2.98-8.28)
Listeria monocytogenes	0.07 (0.04-0.12)	0.11 (0.07-0.18)	0.05 (0.03-0.08)	0.05 (0.03-0.07)	0.08 (0.05-0.12)	0.26 (0.15-0.41)	0.11 (0.06-0.2)	0.4 (0.18-0.77)	0.19 (0.14-0.27)	0.68 (0.42-1.07)	0.07 (0.05-0.11)	0.25 (0.16-0.37)	0.74 (0.49-1.1)	2.6 (1.5-4.35)
**Malaria**	0 (0-0)	0 (0-0)	0 (0-0)	0 (0-0)	0.26 (0.08-0.6)	0.36 (0.11-0.84)	1.49 (0.33-3.78)	2.24 (0.75-5.03)	2.37 (0.83-5.94)	7.34 (2.94-15.76)	0.12 (0.03-0.3)	0.22 (0.06-0.59)	63.83 (29.97-115.29)	245.37 (115.38-429.82)
**Measles**	0 (0-0)	0.03 (0.02-0.04)	0 (0-0)	0 (0-0.01)	0 (0-0)	0 (0-0)	0.76 (0.25-1.73)	6.23 (2.02-14.01)	0.65 (0.22-1.47)	5.71 (1.93-12.97)	0.38 (0.14-0.8)	4.74 (1.78-10)	8.19 (3.11-17.51)	43.19 (15.17-95.11)
***Morganella* spp.**	0.1 (0.06-0.16)	0 (0-0.01)	0.1 (0.07-0.13)	0 (0-0)	0.11 (0.08-0.16)	0.01 (0.01-0.02)	0.04 (0.02-0.06)	0.01 (0-0.02)	0.09 (0.06-0.13)	0.02 (0.01-0.03)	0.06 (0.03-0.1)	0 (0-0.01)	0.03 (0.02-0.04)	0.01 (0.01-0.02)
***Mycoplasma* spp.**	0.87 (0.72-1.06)	1.96 (1.57-2.45)	0.83 (0.68-1)	0.17 (0.12-0.24)	0.89 (0.74-1.08)	2.16 (1.56-2.91)	0.77 (0.6-0.97)	3.55 (2.55-4.77)	1.32 (1.04-1.63)	7.17 (5.13-9.61)	0.75 (0.58-0.97)	1.89 (1.46-2.41)	2.75 (2.16-3.45)	12.69 (9.42-16.88)

Neisseria gonorrhoeae	0.07 (0.06-0.08)	0 (0-0)	0.05 (0.04-0.05)	0 (0-0)	0.07 (0.06-0.07)	0 (0-0)	0.01 (0.01-0.02)	0 (0-0)	0.12 (0.09-0.16)	0 (0-0)	0.03 (0.02-0.03)	0 (0-0)	0.08 (0.06-0.11)	0 (0-0)
Neisseria meningitidis	0.96 (0.54-1.56)	1.83 (1.16-2.8)	0.3 (0.17-0.49)	0.47 (0.29-0.7)	1.11 (0.69-1.69)	5.56 (3.66-8.05)	1.29 (0.76-2.03)	6.38 (3.93-9.9)	2.25 (1.47-3.36)	10.57 (7.07-15.62)	0.89 (0.54-1.37)	3.59 (2.46-5.08)	5.52 (3.95-7.67)	23.6 (16.13-34.04)
**Norovirus**	0.16 (0.03-0.34)	0.49 (0.13-1.1)	0.51 (0.07-1.04)	0.06 (0.02-0.12)	0.82 (0.18-1.6)	2.44 (0.75-4.8)	0.46 (0.13-1.02)	3.38 (0.87-7.84)	2.49 (0.39-6.37)	3.77 (1.09-8.1)	0.65 (0.09-1.61)	0.98 (0.28-2.03)	4.68 (1-10.1)	13.54 (3.55-28.85)
**Other *Klebsiella* species**	1.04 (0.58-1.71)	0.05 (0.02-0.12)	0.77 (0.44-1.29)	0.03 (0.01-0.08)	0.85 (0.46-1.4)	0.15 (0.06-0.32)	0.52 (0.27-0.92)	0.12 (0.04-0.26)	0.73 (0.35-1.28)	0.09 (0.03-0.2)	0.56 (0.3-0.97)	0.1 (0.04-0.21)	0.72 (0.34-1.32)	0.31 (0.11-0.63)
**Other enterococci**	1.65 (0.96-2.64)	0.51 (0.3-0.84)	1.63 (1.13-2.28)	0.21 (0.13-0.34)	1.47 (1.05-2.04)	1.45 (0.94-2.19)	0.79 (0.47-1.23)	1.22 (0.69-1.98)	1.33 (0.86-1.95)	2.61 (1.53-4.18)	1.24 (0.74-1.88)	0.83 (0.53-1.23)	1.06 (0.68-1.58)	3.45 (2.09-5.42)
**Other neglected tropical diseases**	0.04 (0.02-0.05)	0.1 (0.05-0.14)	0.05 (0.02-0.06)	0.04 (0.02-0.05)	0.12 (0.07-0.16)	0.47 (0.29-0.79)	0.05 (0.01-0.06)	0.09 (0.02-0.14)	0.22 (0.12-0.28)	0.25 (0.13-0.37)	0.05 (0.03-0.07)	0.08 (0.05-0.12)	1.02 (0.49-3.39)	4.54 (2.12-15.88)
**Other unspecified infectious diseases**	0.3 (0.18-0.37)	1.64 (0.45-2.7)	0.95 (0.72-1.24)	0.71 (0.52-0.96)	0.6 (0.48-0.84)	1.14 (0.77-1.75)	0.77 (0.62-0.96)	1.27 (0.66-3.04)	1.31 (0.76-1.78)	3.07 (1.41-5.09)	0.62 (0.41-0.73)	1.22 (0.73-1.58)	1.38 (0.84-1.82)	4.8 (2.98-6.62)
**Polymicrobial infections**	1.79 (1.03-2.85)	3.31 (2.05-5.1)	1.87 (1.06-3.08)	1.21 (0.7-1.94)	2.44 (1.54-3.67)	8.7 (5.53-12.63)	2.34 (1.43-3.61)	8.95 (5.31-13.57)	4.07 (2.57-6.13)	21.55 (13.22-32.28)	4.07 (2.38-6.51)	6.58 (4.27-9.59)	4.45 (3.09-6.27)	22.01 (14.94-31.91)
***Proteus* spp.**	2.07 (1.33-3.01)	0.22 (0.14-0.33)	1.87 (1.33-2.55)	0.09 (0.06-0.14)	1.76 (1.23-2.41)	0.62 (0.42-0.88)	0.92 (0.57-1.4)	0.5 (0.3-0.78)	1.48 (0.96-2.18)	0.93 (0.59-1.46)	1.13 (0.72-1.66)	0.29 (0.19-0.43)	1.24 (0.77-1.86)	1.59 (1.04-2.34)
***Providencia* spp.**	0.07 (0.04-0.11)	0 (0-0.01)	0.04 (0.03-0.06)	0 (0-0)	0.09 (0.06-0.13)	0.01 (0.01-0.02)	0.03 (0.01-0.06)	0.02 (0-0.04)	0.12 (0.07-0.18)	0.02 (0.01-0.05)	0.05 (0.02-0.08)	0.01 (0-0.01)	0.04 (0.02-0.07)	0.02 (0.01-0.03)
Pseudomonas aeruginosa	8.47 (5.63-12.2)	4.81 (3.7-6.34)	9.96 (7.34-13.28)	1.13 (0.71-1.68)	8.12 (5.68-11.21)	8.48 (5.81-11.8)	5.08 (3.37-7.32)	9.38 (6.35-12.97)	6.61 (4.5-9.18)	17.89 (12.74-24.09)	6.29 (4.19-9.08)	5.37 (3.9-7.08)	8.04 (5.95-10.65)	26.52 (19.91-34.7)
**Rabies**	0.03 (0.02-0.04)	0 (0-0.01)	0 (0-0)	0 (0-0)	0 (0-0.01)	0 (0-0)	0.01 (0-0.02)	0.01 (0-0.03)	0.44 (0.22-0.64)	0.31 (0.15-0.55)	0.11 (0.04-0.14)	0.07 (0.02-0.13)	0.5 (0.16-0.87)	0.86 (0.26-1.65)
**Respiratory syncytial virus**	0.96 (0.8-1.15)	10.43 (8.35-13.11)	0.58 (0.49-0.64)	1.24 (0.83-1.83)	1.19 (0.9-1.54)	11.17 (7.81-15.14)	1.6 (1.18-2.09)	15.02 (10.77-19.82)	2.81 (2.13-3.58)	29.09 (21.73-37.48)	0.81 (0.65-0.99)	9.06 (7.01-11.36)	6.52 (5.05-8.38)	41.35 (31.8-53.28)
**Rotavirus**	0.06 (0.02-0.11)	0.66 (0.28-1.31)	0.07 (0.03-0.15)	0.1 (0.05-0.18)	0.85 (0.42-1.51)	3.84 (1.75-6.8)	1.24 (0.55-2.26)	11.61 (5.02-21.34)	3.4 (1.47-6.74)	5.79 (2.52-10.48)	0.75 (0.36-1.39)	4.93 (2.53-7.72)	9.5 (4.71-15.86)	52.73 (26.1-87.04)
***Salmonella* Paratyphi**	0 (0-0)	0 (0-0)	0 (0-0)	0 (0-0)	0 (0-0)	0 (0-0)	0 (0-0.01)	0 (0-0.01)	1.2 (0.5-2.34)	1.49 (0.46-3.55)	0.06 (0.02-0.12)	0.12 (0.03-0.29)	0.03 (0.01-0.07)	0.07 (0.02-0.17)
***Salmonella* Typhi**	0.23 (0.1-0.47)	0.4 (0.23-0.65)	0.05 (0.03-0.09)	0.06 (0.03-0.09)	0.37 (0.24-0.56)	1.71 (1.08-2.54)	0.84 (0.53-1.24)	3.57 (2.16-5.37)	5.27 (3.02-8.33)	13.74 (7.96-21.18)	0.78 (0.45-1.26)	2.02 (1.19-3.22)	5.67 (3.87-8.14)	22.09 (14.45-32.42)

**Schistosomiasis**	0 (0-0)	0 (0-0)	0 (0-0)	0 (0-0)	0.11 (0.1-0.12)	0 (0-0)	0.12 (0.1-0.15)	0 (0-0.01)	0 (0-0)	0 (0-0)	0.04 (0.03-0.04)	0 (0-0)	0.96 (0.82-1.13)	0.1 (0.07-0.14)
***Serratia* spp.**	0.94 (0.56-1.46)	0.52 (0.32-0.81)	0.73 (0.44-1.1)	0.18 (0.11-0.28)	1.19 (0.75-1.8)	1.96 (1.29-2.83)	0.94 (0.57-1.48)	1.89 (1.13-2.91)	1.69 (1.01-2.64)	4.53 (2.75-7.1)	1.16 (0.69-1.85)	1.22 (0.8-1.79)	1.89 (1.24-2.85)	6.73 (4.27-10.17)
***Shigella* spp.**	0.06 (0.02-0.12)	0.48 (0.16-1.05)	0.06 (0.02-0.12)	0.03 (0.01-0.06)	0.35 (0.15-0.64)	2.21 (0.91-4.17)	0.38 (0.14-0.85)	3.08 (0.95-7.1)	1.86 (0.71-4.1)	5.62 (2.08-11.28)	0.2 (0.08-0.4)	1.15 (0.43-2.32)	6.51 (2.97-11.28)	29.5 (12.57-53.78)
Staphylococcus aureus	17.58 (12.09-24.64)	8.02 (6.32-10.22)	26.63 (19.94-35.13)	2.11 (1.36-3.1)	17.13 (13.1-22.15)	12.85 (9.05-17.66)	9.67 (6.85-13.47)	13.72 (9.86-18.56)	11.66 (8.68-15.57)	23.11 (17.39-30.03)	12.77 (8.94-17.92)	8.09 (6.19-10.4)	16.18 (13.15-20.21)	41.89 (32.08-54.53)
Streptococcus pneumoniae	9.76 (7.58-12.61)	12.76 (10.27-16.03)	7.93 (6.42-9.84)	1.27 (0.89-1.81)	8.32 (6.72-10.4)	14.82 (10.88-19.5)	6.73 (5.26-8.64)	22.37 (17.19-28.62)	13.05 (10.55-16.18)	45.32 (35.36-57.78)	8.36 (6.6-10.7)	16.21 (13.2-20.12)	21.74 (17.97-26.38)	84.24 (64.8-108.39)
**Syphilis**	0.06 (0.03-0.11)	0.6 (0.2-1.22)	0.03 (0.03-0.05)	0.24 (0.1-0.47)	0.5 (0.25-0.93)	5.29 (2.25-10.4)	0.55 (0.18-1.21)	5.37 (1.61-11.99)	1.05 (0.37-2.24)	10.68 (3.31-23.51)	0.54 (0.19-1.15)	7.62 (2.35-16.92)	6.71 (2.43-13.73)	42.18 (14.84-87.36)
**Tetanus**	0.01 (0.01-0.02)	0 (0-0.01)	0.01 (0.01-0.01)	0 (0-0)	0.08 (0.06-0.15)	0.36 (0.17-0.82)	0.24 (0.15-0.37)	1.36 (0.67-2.45)	0.73 (0.51-1)	5.08 (3.13-7.53)	0.26 (0.14-0.32)	0.67 (0.49-0.91)	1.66 (1.14-2.62)	6.51 (4.46-10.57)
**Tuberculosis**	7.14 (6.52-7.8)	1.71 (1.36-2.13)	1.73 (1.5-1.88)	0.05 (0.04-0.05)	4.4 (3.88-5)	1.34 (1.02-1.7)	3.63 (2.98-4.46)	1.37 (0.9-1.89)	41.22 (36.08-47.22)	8.93 (7.09-11.25)	13.24 (12.1-14.48)	3.23 (2.63-3.92)	51.38 (44.52-59.48)	29.23 (21.93-38.29)
**Varicella and herpes zoster**	0.05 (0.04-0.07)	0.16 (0.1-0.22)	0.32 (0.23-0.63)	0.08 (0.06-0.16)	0.28 (0.21-0.41)	0.61 (0.43-0.93)	0.21 (0.18-0.25)	0.49 (0.32-0.74)	0.51 (0.45-0.58)	0.91 (0.69-1.19)	0.17 (0.15-0.19)	0.33 (0.26-0.41)	0.87 (0.74-1.03)	2.09 (1.55-2.77)
Vibrio cholerae	0.07 (0.04-0.11)	0.37 (0.19-0.71)	0 (0-0)	0 (0-0)	0.18 (0.1-0.3)	0.69 (0.31-1.42)	1.03 (0.58-1.76)	5.54 (2.52-11.71)	0.92 (0.38-1.81)	1.58 (0.75-2.86)	0.15 (0.07-0.3)	0.56 (0.28-1.01)	6.4 (3.39-10.91)	17.58 (7.9-31.8)
**Viral meningitis**	0.14 (0.09-0.23)	0.17 (0.11-0.27)	0.08 (0.05-0.14)	0.07 (0.05-0.1)	0.15 (0.1-0.22)	0.38 (0.24-0.55)	0.22 (0.13-0.38)	0.66 (0.32-1.34)	0.35 (0.27-0.47)	0.99 (0.69-1.44)	0.11 (0.08-0.18)	0.34 (0.24-0.47)	1.44 (1.03-1.95)	4.03 (2.59-5.96)
**Visceral leishmaniasis**	0.01 (0-0.09)	0.04 (0-0.35)	0 (0-0.02)	0 (0-0.04)	0.2 (0-0.78)	0.43 (0-1.7)	0.07 (0-0.7)	0.2 (0-1.82)	0.08 (0-0.44)	0.18 (0-1.02)	0 (0-0)	0 (0-0)	0.29 (0.15-0.45)	0.59 (0.3-0.96)
**Yellow fever**	0 (0-0)	0 (0-0)	0.01 (0-0.02)	0.01 (0-0.02)	0.01 (0-0.04)	0.01 (0-0.03)	0.06 (0.01-0.19)	0.04 (0.01-0.18)	0 (0-0)	0 (0-0)	0 (0-0)	0 (0-0)	0.37 (0.14-0.77)	0.29 (0.1-0.63)
**Zika virus**	0 (0-0)	0 (0-0)	0 (0-0)	0 (0-0)	0 (0-0)	0 (0-0)	0 (0-0)	0 (0-0)	0 (0-0)	0 (0-0)	0 (0-0)	0 (0-0)	0 (0-0)	0 (0-0)
**Total**	**162.98 (126.84-209.56)**	**93.61 (74.48-118.99)**	**157.92 (127.27-197.59)**	**18.62 (12.73-26.53)**	**147.11 (119.27-182.32)**	**176.27 (129.67-233.45)**	**111.35 (87.52-142.39)**	**249.89 (190.28-316.25)**	**216.78 (183.05-257.23)**	**484.6 (379.28-614.7)**	**151.92 (124.44-187.54)**	**150.64 (121.09-184.21)**	**456.09 (398.18-525.66)**	**1316.79 (1073.95-1613.48)**

To compute the death rate per 100 000 population associated with specific pathogens in each GBD super-region for all ages and those under 5 years of age in 2019, we first established the total population size using estimates from GBD 2019[4] within each GBD super-region for the specified year. Next, we tallied the total number of deaths attributed to each specific pathogen within these super-regions during the same year. Subsequently, we have divided the number of deaths associated with each pathogen by the corresponding total population in the respective age group and super-region and multiplied this quotient by 100 000 to express the death rate per 100 000 population.

Supplementary Table 2: Source counts for 57 pathogens estimated by GBD 2019. Source counts are presented as nid, year, location, combinations.

Cause name	Source Counts
**Adenovirus**	204
***Aeromonas* spp.**	149
**African trypanosomiasis**	2944
**Ascariasis**	3395
**Bordetella species (Pertussis)**	11093
***Campylobacter* spp.**	272
**Chagas disease**	2097
Clostridium difficile	107
**Cryptosporidium**	202
**Cutaneous and mucocutaneous leishmaniasis**	1056
**Cystic echinococcosis**	3532
**Cysticercosis**	3442
**Dengue**	5418
**Diphtheria**	3731
**Ebola**	51
Entamoeba histolytica	162
**Enteropathogenic *Escherichia coli***	217
**Enterotoxigenic *Escherichia coli***	234
**Food-borne trematodiases**	57
**Genital herpes**	338
**Guinea worm disease**	436
**Hepatitis A**	3053
**Hepatitis B**	468
**Hepatitis C**	332
**Hepatitis E**	2655
**HIV/AIDS**	5162
**Hookworm disease**	168
**Human papillomavirus**	7319
**Influenza virus**	370
**Leprosy**	1685
**Lymphatic filariasis**	561
**Malaria**	10803
**Measles**	12085
Neisseria gonorrhoeae	3985
**Non-typhoidal Salmonella (diarrhea)**	303
**Norovirus**	204
**Onchocerciasis**	351
**other neglected tropical diseases**	3651

**Other unspecified infectious diseases**	4261
**Rabies**	3527
**Respiratory syncytial virus**	312
**Rotavirus**	744
Salmonella Paratyphi	3422
**Schistosomiasis**	3923
***Shigella* spp.**	276
**Stomach cancer**	3659
**Syphilis**	4913
**Tetanus**	3991
**Trachoma**	135
**Trichomoniasis**	137
**Trichuriasis**	156
**Tuberculosis**	4700
**Varicella and herpes zoster**	2874
Vibrio cholerae	3685
**Visceral leishmaniasis**	4521
**Yellow fever**	2777
**Zika virus**	485
**Total**	140790

Supplementary Table 3: Source counts for 28 pathogens estimated as part of the Global Research on Antimicrobial Resistance. Source counts are presented as number of isolates.

Pathogen	Number of isolates
Acinetobacter baumannii	99,511
***Chlamydia* spp.**	15,577
***Citrobacter* spp.**	127,353
***Enterobacter* spp.**	399,917
Enterococcus faecalis	940,348
Enterococcus faecium	524,379
Escherichia coli	7,783,843
**Fungi**	1,438,888
**Group A *Streptococcus***	363,986
**Group B *Streptococcus***	219,335
Haemophilus influenzae	96,938
Klebsiella pneumoniae	1,705,653
***Legionella* spp.**	5,323
Listeria monocytogenes	16,300
***Morganella* spp.**	124,288
***Mycoplasma* spp.**	57,597
Neisseria meningitidis	37,641
**Other *Enterococci***	37,191
**Other *Klebsiella* species**	166,774
**Polymicrobial infections**	4,435
***Proteus* spp.**	670,723
***Providencia* spp.**	35,773
Pseudomonas aeruginosa	1,252,431
Salmonella Typhi	16,450
***Serratia* spp.**	106,321
Staphylococcus aureus	4,453,097
Streptococcus pneumoniae	982,319
**Viral meningitis**	170,568
**Total**	21,852,959

Supplementary Table 4: All-cause disability-adjusted life-years (DALYs) in counts and rates, for each GBD super-region, for all ages and under 5 years of age, both sexes, 2019 (Sources: GBD 2019 Diseases and Injuries Collaborators. Global burden of 369 diseases and injuries in 204 countries and territories, 1990–2019: a systematic analysis for the Global Burden of Disease Study 2019. Lancet 2020; 396: 1204–22; https://vizhub.healthdata.org/gbd-results/)

Location	Age	Metric	Mean Value	Upper	Lower
Sub-Saharan Africa	<5 years	Number	243891734.25	294668873.64	204720104.32
Sub-Saharan Africa	<5 years	Rate	147218.02	177868.14	123573.23
Sub-Saharan Africa	All ages	Number	510630214.28	584636593.89	450576878.91
Sub-Saharan Africa	All ages	Rate	47359.10	54222.92	41789.37
Latin America and Caribbean	<5 years	Number	18091597.29	22148493.34	14756413.94
Latin America and Caribbean	<5 years	Rate	37632.75	46071.60	30695.16
Latin America and Caribbean	All ages	Number	166067802.37	188607514.56	147230849.64
Latin America and Caribbean	All ages	Rate	28417.86	32274.91	25194.45
Southeast Asia, East Asia, and Oceania	<5 years	Number	39641447.77	45519251.39	34687635.62
Southeast Asia, East Asia, and Oceania	<5 years	Rate	28219.73	32403.98	24693.23
Southeast Asia, East Asia, and Oceania	All ages	Number	601252753.03	673101421.46	532376316.00
Southeast Asia, East Asia, and Oceania	All ages	Rate	27845.29	31172.75	24655.48
Central Europe, Eastern Europe, and Central Asia	<5 years	Number	6260246.00	7260055.57	5423229.43
Central Europe, Eastern Europe, and Central Asia	<5 years	Rate	22714.08	26341.70	19677.13
Central Europe, Eastern Europe, and Central Asia	All ages	Number	157814480.72	175327139.48	142714306.01
Central Europe, Eastern Europe, and Central Asia	All ages	Rate	37779.50	41971.89	34164.64
High-income	<5 years	Number	6409828.87	7180077.32	5721419.37
High-income	<5 years	Rate	11256.79	12609.48	10047.82
High-income	All ages	Number	323732733.12	368067442.92	284120170.63
High-income	All ages	Rate	29865.30	33955.31	26210.93
North Africa and Middle East	<5 years	Number	28813099.05	33501933.66	24842579.83
North Africa and Middle East	<5 years	Rate	48247.07	56098.45	41598.50
North Africa and Middle East	All ages	Number	163781896.54	186172200.68	143723029.83
North Africa and Middle East	All ages	Rate	26906.23	30584.53	23610.94
South Asia	<5 years	Number	127858088.49	150677267.16	108963941.03
South Asia	<5 years	Rate	77770.45	91650.35	66277.97
South Asia	All ages	Number	614740190.59	688694087.90	547579224.87
South Asia	All ages	Rate	34053.85	38150.56	30333.43
Global	**<5 years**	**Number**	**470966042.00**	**557758708.00**	**402932837.00**
Global	**<5 years**	**Rate**	**71052.46**	**84146.47**	**60788.61**
Global	**All ages**	**Number**	**2538020071.00**	**2810205655.00**	**2285262551.00**
Global	**All ages**	**Rate**	**32801.70**	**36319.46**	**29535.03**

Supplementary Table 5: Pathogen-associated disability-adjusted life-years (DALYs) counts + 95% UIs, for each super-region, all ages in 2019

	DALYs Count Mean							DALYs Count Lower							DALYs Count Upper						
**Location name**	**Central Europe, Eastern Europe, and Central Asia**	**High-income**	**Latin America and Caribbean**	**North Africa and Middle East**	**South Asia**	**Southeast Asia, East Asia, and Oceania**	**Sub-Saharan Africa**	**Central Europe, Eastern Europe, and Central Asia**	**High-income**	**Latin America and Caribbean**	**North Africa and Middle East**	**South Asia**	**Southeast Asia, East Asia, and Oceania**	**Sub-Saharan Africa**	**Central Europe, Eastern Europe, and Central Asia**	**High-income**	**Latin America and Caribbean**	**North Africa and Middle East**	**South Asia**	**Southeast Asia, East Asia, and Oceania**	**Sub-Saharan Africa**
Acinetobacter baumannii	421479.01	778465.71	855077.99	975180.95	5025644.52	4333959.08	4305684.83	249030.52	459145.78	530241.69	594679.01	3294898.41	2600190.13	2959672.72	668453.78	1226805.12	1282346.36	1490808.84	7373785.40	6577234.72	6130184.14
**Adenovirus**	10377.30	30662.73	70812.62	195453.61	1367377.47	180742.26	4101554.73	5647.14	17017.84	39119.21	86617.16	755289.32	101464.51	2147760.46	18317.06	49731.81	131136.85	411060.88	2353067.89	318594.69	6973830.07
***Aeromonas* spp.**	5991.11	2071.10	15292.02	55907.67	478881.82	42974.28	991766.69	2785.63	949.49	7137.05	20528.82	218883.94	17989.10	398028.28	10597.61	3601.81	29028.44	117279.44	859964.57	82556.36	1921581.11
**African trypanosomiasis**	0.00	0.00	0.00	0.00	0.00	0.00	82615.46	0.00	0.00	0.00	0.00	0.00	0.00	37636.49	0.00	0.00	0.00	0.00	0.00	0.00	155791.81
**Ascariasis**	787.84	267.80	36756.07	24542.04	372517.65	90931.70	268088.22	461.72	157.03	22038.84	15286.04	211674.63	54344.01	197364.65	1286.92	441.26	59813.41	37786.62	619326.67	145558.79	355866.75
***Bordetella* spp. (Pertussis)**	33337.64	11965.57	203475.72	735082.23	2584988.93	1004900.74	6934772.06	13149.00	7992.95	92201.38	275165.92	664210.00	415162.22	2822826.32	69681.43	17517.96	397574.30	1576850.30	5946530.07	2008119.30	13755209.65
***Campylobacter* spp.**	57049.93	74613.67	134996.86	77609.59	2510234.11	303523.63	3112495.30	16162.90	17059.21	50180.95	25219.36	966969.62	96564.29	1193850.64	127184.16	166276.58	276527.34	181032.69	5153936.81	731934.58	6397623.32
**Chagas disease**	0.00	30338.96	257022.93	0.00	0.00	0.00	0.00	0.00	20001.63	170189.14	0.00	0.00	0.00	0.00	0.00	71049.05	422171.17	0.00	0.00	0.00	0.00
***Chlamydia* spp.**	91993.92	95846.16	179493.12	290133.83	1828331.70	531299.15	2559145.39	75299.25	76992.83	137782.95	213296.67	1392275.10	404462.93	1988992.28	113480.60	121462.43	233266.50	382961.32	2338574.69	686912.71	3311591.04
***Citrobacter* spp.**	98280.82	115962.21	115340.70	110790.93	586777.03	463367.93	453734.00	52397.19	73688.37	73929.14	63330.68	350832.37	272392.03	273596.81	170506.67	172850.02	169264.68	174720.82	899046.31	723581.72	696262.55

Clostridioides difficile	249426.45	643376.83	191337.79	111053.57	112281.36	698278.85	119683.75	147038.94	458189.65	114325.92	41961.25	42348.59	329086.49	59517.08	370040.99	859635.63	288422.71	261088.40	260684.94	1503421.66	219777.97
***Cryptosporidium* spp.**	15999.59	13603.62	51132.41	185810.38	1307472.18	84504.24	4644500.07	2792.25	2197.07	8369.35	31126.12	222539.85	13683.64	1077845.93	51596.92	46055.12	165784.70	540788.98	4229274.07	292490.13	11048538.90
**Cutaneous and mucocutaneous leishmaniasis**	237.43	39.35	12842.01	242952.60	28724.01	94.48	8309.09	128.18	22.45	8281.18	152541.15	17525.72	10.12	5340.25	409.14	63.56	19413.39	362599.84	42575.77	298.53	12121.16
**Cystic echinococcosis**	41209.16	3062.32	875.68	33913.53	36566.65	9719.47	34938.85	24394.22	2274.41	530.23	24861.47	26934.38	7245.67	21557.81	67754.80	3943.64	1282.05	45345.22	47458.86	13117.60	49932.48
**Cysticercosis**	100854.85	123335.61	290308.29	730.04	390435.67	211048.86	269577.15	58924.20	72154.44	185774.83	395.97	236737.34	119538.59	173112.85	155230.36	191490.42	410915.79	1294.32	568336.76	327660.36	383393.38
**Dengue virus**	6.98	2731.13	120825.06	11958.97	1260308.57	1067342.29	52461.28	3.35	1094.15	90719.48	5026.43	281095.65	464986.87	12511.09	17.35	5718.59	144665.25	23535.56	1908641.20	1327023.99	113789.54
**Diphtheria**	673.21	410.19	2087.50	10610.40	30258.56	13635.34	801041.89	438.75	348.18	1440.58	6963.79	21932.52	10857.62	519960.50	1019.60	488.13	3068.14	15764.86	39991.42	16875.55	1182076.50
**Ebola virus**	0.00	0.00	0.00	0.00	0.00	0.00	195394.45	0.00	0.00	0.00	0.00	0.00	0.00	160083.66	0.00	0.00	0.00	0.00	0.00	0.00	230578.17
Entamoeba histolytica	15189.81	4996.34	87710.72	134859.59	911913.66	32038.56	1100023.53	4693.41	1462.45	26972.85	35986.90	271303.93	9064.08	306589.19	36205.25	12437.22	199929.77	350917.01	2251885.23	83477.17	2623548.71
***Enterobacter* spp.**	408331.83	703525.59	660535.32	668013.21	3223704.68	3180388.98	2248324.55	260433.18	469304.83	446086.26	417958.52	2131778.79	1974269.94	1631107.46	598204.05	1000382.74	947548.62	1006403.67	4671176.82	4832297.38	3107007.07
Enterococcus faecalis	532747.91	817954.62	548957.97	377984.74	1781642.11	1333993.90	1582682.47	320958.85	504919.83	356242.65	219742.99	1143342.42	791894.05	1045360.00	805713.75	1225526.28	801699.57	595318.03	2630481.39	2062459.26	2258271.25
Enterococcus faecium	515524.43	956626.52	521896.26	363262.00	1273445.30	1400437.45	970720.85	308470.80	598704.73	324269.50	210939.44	738192.65	827972.76	606342.58	788137.94	1424602.33	774539.29	576495.09	2029241.41	2229958.57	1490852.22
**Enteropathogenic *Escherichia coli***	3641.15	3687.01	7783.61	50586.27	293470.42	53198.07	888866.57	1629.50	1460.35	3691.91	22385.18	136169.94	24724.03	416887.31	6542.43	7040.47	14069.71	98015.87	536233.80	98442.45	1690506.44

**Enterotoxigenic *Escherichia coli***	15177.20	22936.07	10431.10	82478.28	780472.72	81340.40	503700.08	6739.45	10479.57	4480.09	32817.36	334496.78	33911.21	200679.58	28623.64	42606.32	21570.04	184393.58	1532623.45	161150.97	1077591.97
Escherichia coli	1998302.43	3453818.38	1877774.46	1485407.40	7291157.41	4345885.90	8011955.38	1288468.67	2358765.50	1345175.62	1018815.30	5456467.02	2935060.08	6139036.44	2939209.70	4898678.65	2572679.05	2100491.64	9449364.82	6242384.39	10469382.62
**Food-borne trematodiases**	17648.57	23788.72	34922.48	16526.63	0.00	687203.18	0.00	7666.39	15520.17	11575.00	3938.12	0.00	316069.02	0.00	31739.32	34599.79	67597.25	33357.83	0.00	1304133.54	0.00
**Fungi**	254559.20	273017.25	661965.49	839720.96	5437081.58	1494356.54	9566379.23	161142.72	196023.03	433642.46	472997.68	3094675.84	953824.52	5832504.44	389504.92	378997.06	955408.87	1337109.92	8580101.10	2192820.63	14715646.19
**Genital herpes**	12286.76	36879.85	34732.66	15003.70	32017.30	70949.22	50993.93	3912.86	11570.38	11139.51	4859.00	10430.83	22434.54	16935.45	30193.02	89908.91	84991.77	37069.76	80339.61	176517.16	123036.66
**Group A Streptococcus (*Streptococcus pyogenes*)**	413062.57	689004.99	536247.33	416985.55	1595522.36	1312692.94	1723252.73	229721.49	410291.31	347259.27	237315.80	973926.01	708513.82	1151495.04	702430.74	1138190.03	835423.95	698004.77	2638943.51	2358456.90	2550138.90
**Group B Streptococcus (*Streptococcus agalactiae*)**	356423.08	711987.81	593433.72	645939.27	3051021.99	1610251.83	4234346.25	241873.43	480694.50	411099.00	426748.84	2219930.85	1064552.16	3257060.18	510752.43	1021627.91	820879.57	926137.44	4025041.22	2318883.67	5504585.97
**Guinea worm disease**	0.00	0.00	0.00	0.00	0.00	0.00	0.81	0.00	0.00	0.00	0.00	0.00	0.00	0.46	0.00	0.00	0.00	0.00	0.00	0.00	1.34
**HIV/AIDS**	1689224.62	836852.28	2422366.50	558575.76	3201530.05	4431504.89	38961504.27	1630877.76	724668.96	2256543.86	320452.04	2547705.38	3842011.01	33487393.24	1765062.12	989787.48	2708902.95	1124478.87	5116473.86	5190371.22	47420163.21
Haemophilus influenzae	128288.26	162562.02	195179.59	257177.70	1357522.88	564985.48	2431072.60	107148.39	134106.76	152033.21	198129.76	1048554.39	450088.99	1873701.18	154088.43	201139.47	247267.47	333203.28	1707485.51	712551.93	3132511.05
Helicobacter pylori	1354370.79	2179150.29	1158981.37	747675.04	2066871.14	8163116.25	713639.90	1177875.08	1904132.64	987257.23	632100.05	1721686.56	6691905.87	592826.79	1508491.87	2388073.22	1336965.48	861136.46	2422671.38	9621575.72	835071.13

**Hepatitis A**	18104.34	30310.42	38959.74	173922.27	2335800.41	234526.26	476468.36	13320.06	21527.53	30407.23	76032.56	1540806.03	144763.57	287058.42	23415.71	41470.18	48008.44	317537.30	3393595.25	348101.20	824019.10
**Hepatitis B**	1207753.83	1221173.24	504300.04	1355671.89	6631138.33	9600453.18	3391042.32	973624.04	1062533.03	429915.28	1006894.26	5503994.35	8171016.95	2710992.23	1470850.27	1404051.59	587527.39	1777753.10	8012619.07	11093127.38	4192156.43
**Hepatitis C**	1062197.05	2661524.49	1031721.92	1530637.61	3227554.93	4244071.18	1530829.67	875368.07	2390518.76	876195.28	1123074.09	2688176.47	3637908.69	1229010.85	1287404.32	2960326.95	1227242.31	1959050.44	3830012.98	4908995.19	1885610.13
**Hepatitis E**	2222.74	2987.93	3382.20	6901.20	104165.32	35784.22	22834.89	1501.70	2022.52	2546.47	4198.12	55265.22	22255.32	13588.77	3019.01	4106.07	4608.22	15499.07	177486.04	47445.89	40882.67
**Hookworm disease**	3470.23	2116.90	45445.49	23590.81	231026.45	136669.17	541540.68	2179.33	1205.82	27930.35	14519.88	141052.02	82380.24	342703.08	5141.03	3438.86	68496.94	36840.16	355893.09	217586.91	809496.80
**Human papillomavirus**	663182.50	854765.02	1122263.94	237977.53	1967192.20	2712324.66	2046056.71	572601.29	751321.94	985360.75	181339.76	1572323.74	1901789.46	1573543.75	752150.82	911760.28	1297229.85	287406.17	2544065.80	3252111.28	2522312.63
**Influenza virus**	466667.04	1040657.26	887771.59	886003.03	4473124.59	1864905.76	7078769.82	416309.18	923798.39	765734.77	707207.96	3525691.14	1584484.27	5573847.59	528103.45	1137479.29	1032276.99	1082918.10	5566149.35	2203856.77	8989664.88
**Invasive Non-typhoidal *Salmonella* (iNTS)**	160495.05	159447.37	286310.36	564281.41	2531838.43	1034241.73	10194115.85	72689.07	35229.66	131279.67	291573.13	1536140.49	545113.96	6025327.09	301720.64	416199.85	558901.22	1085910.41	4028108.52	1927428.58	15924411.34
Klebsiella pneumoniae	1193976.88	1846561.17	1793796.98	1714444.86	8438114.01	4224070.92	11871674.58	815633.29	1286516.48	1271482.03	1166842.42	6148752.83	2952871.42	9173492.38	1689394.99	2598162.11	2448017.95	2447314.11	11408769.38	5886156.52	15378934.25
***Legionella* spp.**	139148.79	317768.89	180372.04	186131.05	924136.24	497610.74	973856.43	120337.73	272509.21	147048.43	132945.72	632749.01	393729.03	654923.73	162569.65	374077.97	225232.40	257500.33	1334787.86	639081.92	1448179.89
**Leprosy**	20.49	44.76	2924.19	587.07	16145.64	3007.86	6108.20	10.28	27.98	1844.39	362.22	10392.90	1894.76	3904.03	39.01	67.53	4333.83	872.45	23627.53	4402.86	9068.43
Listeria monocytogenes	12240.50	14930.70	23842.38	41029.74	203722.48	71563.16	553956.72	8048.12	9687.43	15349.26	22485.29	141665.77	47657.94	346243.58	20583.50	25820.85	36180.19	73367.20	291433.20	110535.86	848336.78
**Lymphatic filariasis**	0.00	201.63	18140.05	22716.75	872473.11	296078.23	419039.64	0.00	106.60	11212.18	12109.68	513126.73	175607.57	248207.20	0.00	363.71	29001.05	41248.80	1444167.78	490890.63	703236.57
**Malaria**	120.18	99.55	97453.40	549344.84	2873163.17	159144.29	49967779.48	87.10	72.46	38095.73	170077.22	1157401.31	61943.03	24706719.56	158.22	147.54	208641.12	1263410.18	6633300.02	364835.99	86809317.19

**Measles**	1372.11	475.20	97.33	398284.78	1006188.25	695513.24	7335238.85	1039.30	260.24	73.79	129196.98	339442.56	261888.86	2650117.39	1749.96	587.51	170.39	899157.87	2270833.83	1473606.23	15963533.06
***Morganella* spp.**	8512.50	13590.08	11854.61	4669.76	38306.64	24109.74	8181.70	5429.62	9871.79	8304.36	2133.53	24186.20	12784.60	4649.38	12930.32	19112.59	16644.52	8448.76	56464.02	40736.62	12991.51
***Mycoplasma* spp.**	145783.91	153385.89	207731.52	277092.46	1406601.05	554497.09	2205588.08	124342.10	123177.08	165487.92	212470.31	1080995.06	434715.25	1684604.26	172862.81	195035.35	258601.77	354646.32	1800616.08	711575.73	2832210.20
Neisseria gonorrhoeae	11836.44	12141.73	16889.08	7620.08	97577.28	31719.62	53269.99	10192.70	10551.96	14815.77	5588.27	70243.46	24769.85	41384.76	13890.35	13992.67	19407.32	10344.94	121959.56	39470.23	65961.50
Neisseria meningitidis	178150.54	118744.62	398546.69	528355.98	2606403.65	1002301.60	4558082.56	102459.96	67218.54	253597.91	316379.96	1737177.19	641953.17	3238159.60	285690.76	188594.03	587299.97	821207.57	3877264.00	1516054.82	6406692.09
**Norovirus**	146593.57	200729.49	316075.99	318469.59	1625575.64	699495.51	3249296.79	24787.13	29926.46	79146.23	81868.30	325977.31	122860.05	761356.86	294241.66	392794.91	608042.06	709507.92	3692103.01	1681079.02	6770588.60
**Onchocerciasis**	0.00	0.00	472.84	4422.89	0.00	0.00	1225537.68	0.00	0.00	184.70	2853.34	0.00	0.00	761435.36	0.00	0.00	873.91	6416.81	0.00	0.00	1814391.67
**Other *Klebsiella* spp.**	124405.98	163809.78	135121.07	87485.95	424228.03	334665.37	283195.46	65984.96	94467.39	72119.91	44146.41	195798.35	169321.16	124562.06	211687.49	268398.54	228659.39	154966.68	781172.39	587471.70	523611.03
**Other *Enterococcus* spp.**	151930.22	277860.50	213581.54	158525.42	824392.49	646808.33	658486.09	89345.53	187342.49	148913.31	93311.52	538945.40	394868.45	422651.48	244354.24	398684.08	301445.42	251438.19	1244420.00	958136.84	1008603.21
**Other neglected tropical diseases**	54312.74	50445.15	120046.97	114894.92	1093617.62	214614.04	1415525.07	37327.77	34704.77	87298.96	77197.57	768787.17	147573.72	877191.32	77191.75	72774.68	164219.47	167241.98	1494037.20	302891.95	3406254.55
**Other unspecified infectious diseases**	112563.16	319749.38	237047.71	301488.29	1959021.79	748031.83	1405566.16	71475.90	252352.66	187782.13	237004.25	1336608.52	539078.91	995004.16	150060.36	417740.88	310751.73	410242.15	2548953.09	897695.74	1785807.35
**Polymicrobial infections**	218280.33	428280.38	585854.29	685415.92	3910022.95	2540782.21	3520578.73	132926.97	241919.12	381596.43	419261.36	2474691.24	1547593.27	2419373.42	338016.22	694098.43	851537.82	1040351.34	5838908.57	3946673.64	5051670.00

***Proteus* spp.**	189265.54	314786.59	227192.60	143837.56	731538.05	549859.32	516381.74	120960.84	217864.64	156422.31	85934.32	472263.70	348129.92	334843.71	274424.50	438728.32	318726.65	222991.34	1066548.62	814447.70	768373.79
***Providencia* spp.**	6246.33	6396.39	10435.19	5203.41	54446.67	22753.35	14161.09	3963.76	4432.08	7035.37	2244.06	35268.60	12097.10	7828.75	9831.18	9466.10	15112.67	9814.36	83620.09	38145.18	22778.58
Pseudomonas aeruginosa	902189.23	1767681.96	1293650.07	1101106.90	4734218.69	3398394.26	5095306.43	605148.54	1236422.18	887680.38	724181.21	3301721.98	2261000.03	3785155.20	1293985.71	2434534.63	1815528.10	1582976.21	6516486.57	4908045.92	6701577.34
**Rabies**	3336.98	260.23	796.76	4217.16	403704.30	116213.29	366627.08	2444.77	234.66	485.74	1182.39	198739.08	36990.80	120275.90	4849.37	291.65	1141.91	6309.43	594629.62	166912.84	653464.21
**Respiratory syncytial virus**	286683.73	136783.82	512559.95	820116.09	4356701.09	1223337.10	6134581.92	233897.86	113754.42	366859.81	593668.65	3270138.92	953034.99	4732983.11	352525.88	168595.61	685918.73	1077253.71	5607070.04	1524329.00	7909267.33
**Rotavirus**	89053.34	94687.86	429223.53	947755.37	2980363.19	1322332.74	8528326.37	37788.44	38014.56	211973.38	457348.89	1394758.97	659544.83	4223923.67	167051.96	185591.97	765536.94	1693944.33	5769425.97	2405133.43	14183091.13
***Salmonella* Paratyphi**	158.37	95.20	255.09	1745.44	1518763.24	90505.99	28231.04	25.12	48.96	125.74	625.54	626613.81	35635.49	10715.03	277.16	114.27	482.99	3754.94	2941513.13	185729.40	60857.05
***Salmonella* Typhi**	39221.92	17180.54	123953.58	360154.62	6839437.54	1108888.98	4751108.63	18693.24	8986.07	80074.15	224052.12	3822076.91	617929.18	3223904.50	75731.22	30047.08	184921.76	544599.60	10908469.37	1825942.43	6836673.13
**Schistosomiasis**	0.00	0.00	96384.82	106936.38	0.00	98829.47	1368350.83	0.00	0.00	57166.51	62982.42	0.00	55413.40	894277.07	0.00	0.00	164275.67	182335.68	0.00	176712.74	2156431.19
***Serratia* spp.**	109577.99	163638.90	234951.18	223603.00	1266594.76	732828.29	1283170.23	64797.00	99945.18	150314.44	133505.10	779902.40	453732.44	837600.50	168928.40	249377.61	346346.62	350281.39	1992700.12	1128422.17	1900887.23
***Shigella* spp.**	37491.08	23162.65	153046.63	231642.26	1514905.45	252141.26	5258755.27	14990.96	9285.56	66591.02	85593.55	597518.70	105059.99	2319536.82	76383.36	48385.41	285280.52	508180.68	3054281.37	498090.97	9245814.31
Staphylococcus aureus	1897958.86	4662982.50	2537003.95	1970902.06	7581138.27	6657179.40	9208146.72	1321525.34	3335146.22	1872540.41	1378524.55	5658039.03	4598485.36	7350705.29	2647864.26	6402098.36	3373181.65	2730052.29	9948686.83	9388155.15	11656110.07
Streptococcus pneumoniae	1325787.26	1384470.02	1607484.52	2010077.46	10763554.50	5402159.08	15623095.02	1064706.66	1082273.29	1251498.57	1570858.78	8724567.72	4289400.50	12476678.14	1657863.41	1795561.93	2055040.52	2586541.60	13310973.43	6840497.95	19563219.20
**Syphilis**	18946.47	23709.02	243900.45	292841.17	1620468.91	997003.37	6344173.67	8994.09	16248.63	112165.27	92183.73	541968.42	332988.02	2257447.29	34662.16	35554.97	463085.79	646464.13	3500314.81	2171036.24	13047847.46
**Tetanus**	946.65	1473.83	26168.56	98689.53	939317.70	244150.47	1300979.02	688.87	1140.55	16234.66	60021.58	638969.79	147366.46	913510.25	1979.51	3165.15	49092.79	159001.48	1319273.35	303212.02	2112938.43

**Trachoma**	0.00	2.73	1087.66	4768.73	84015.91	15150.83	75484.43	0.00	1.43	714.72	2630.04	51401.83	8689.17	49938.24	0.00	4.81	1583.36	7846.91	132672.80	25464.13	108276.65
**Trichomoniasis**	13319.37	30003.32	38335.39	14554.19	40591.23	75044.00	75345.66	5155.71	11779.94	15091.01	5586.99	15714.59	29711.33	29484.93	27521.65	61656.61	78970.91	29670.90	84059.10	157134.63	154110.11
**Trichuriasis**	0.00	35.90	23168.68	568.24	55829.30	92533.98	63379.81	0.00	16.65	12587.67	294.02	28805.90	48446.06	35033.26	0.00	66.79	38785.05	991.85	98993.95	154185.72	105279.01
**Tuberculosis**	1255193.51	339168.77	978397.37	877461.49	27543150.04	9658514.35	24406716.84	1149382.94	311424.85	860436.97	715442.16	24390598.77	8794272.78	21000496.23	1367853.12	360861.43	1112640.48	1071330.51	31124032.89	10585348.55	28452244.78
**Varicella and herpes zoster**	16620.22	89192.26	83643.80	73696.30	398114.95	197302.03	568991.32	12603.04	67433.61	64548.75	58931.55	339542.87	161660.43	457887.19	22157.12	133269.11	117293.61	92562.06	464630.79	243705.57	694278.36
Vibrio cholerae	74343.72	0.00	112466.72	801312.84	1021961.62	269159.14	4470679.95	40828.20	0.00	61658.40	394657.57	423805.35	126674.46	2300721.42	124405.32	0.00	198130.32	1529118.30	2164324.12	526039.65	7690828.17
**Viral meningitis**	35096.31	41896.49	54886.97	97525.94	441464.69	151694.72	1125140.30	23835.42	28536.60	38511.97	57300.20	337243.88	103905.49	783664.42	56255.16	67638.35	79038.77	171625.39	586191.92	239396.32	1554152.48
**Visceral leishmaniasis**	2532.98	1002.61	71995.39	30526.27	94446.72	120.28	234946.86	6.29	3.86	110.64	69.85	145.60	12.00	123752.69	25674.70	9313.26	301074.87	311828.21	559700.76	502.26	368520.73
**Yellow fever**	0.00	2952.57	5014.37	23847.86	0.00	0.00	269565.58	0.00	462.28	1540.91	4231.18	0.00	0.00	99518.36	0.00	9683.43	12901.92	73600.83	0.00	0.00	561216.50
**Zika virus**	0.00	12.43	323.86	0.00	0.00	0.00	11.13	0.00	2.75	234.94	0.00	0.00	0.00	2.44	0.00	28.56	428.57	0.00	0.00	0.00	30.72
**TOTAL**	**21229794.78**	**31796918.74**	**29036926.35**	**30144982.74**	**175024431.29**	**103081751.51**	**314027503.76**	**17198854.81**	**25410445.71**	**23766873.48**	**24083316.32**	**149176480.33**	**85980435.27**	**270002926.18**	**26311145.48**	**40072459.86**	**35548076.91**	**37914866.83**	**207195469.09**	**125082803.62**	**367917737.19**

Supplementary Table 6: Pathogen-associated disability-adjusted life-years (DALYs) counts + 95% UIs for each super-region for the under 5 years age group in 2019.

	**DALYs Count Mean**							**DALYs Count Lower**							**DALYs Count Upper**						
**Location Name**	**Central Europe, Eastern Europe, and Central Asia**	**High-income**	**Latin America and Caribbean**	**North Africa and Middle East**	**South Asia**	**Southeast Asia, East Asia, and Oceania**	**Sub-Saharan Africa**	**Central Europe, Eastern Europe, and Central Asia**	**High-income**	**Latin America and Caribbean**	**North Africa and Middle East**	**South Asia**	**Southeast Asia, East Asia, and Oceania**	**Sub-Saharan Africa**	**Central Europe, Eastern Europe, and Central Asia**	**High-income**	**Latin America and Caribbean**	**North Africa and Middle East**	**South Asia**	**Southeast Asia, East Asia, and Oceania**	**Sub-Saharan Africa**
Acinetobacter baumannii	54447.041	30338.252	220459.132	376599.397	2354436.827	580175.611	2961848.272	34381.951	17538	141422.141	229229.148	1420552.035	381774.711	1883445.249	83934.095	47616.778	327067.148	580561.038	3621483.658	853425.224	4514263.321
**Adenovirus**	5431.62	10126.857	57999.955	174617.104	925670.051	136283.088	3737472.295	2567.969	4653.523	28486.804	70999.919	451419.67	66395.846	1866008.258	11095.489	19326.016	114853.886	377555.82	1755021.925	261202.277	6534549.821
***Aeromonas* spp.**	2874.415	425.958	9489.933	44593.667	327841.706	15599.974	837493.448	1079.312	150.403	3489.131	14502.695	134407.979	5606.679	310760.576	5962.558	881.399	20748.705	102891.963	613878.928	32700.979	1713108.3
**African trypanosomiasis**	0	0	0	0	0	0	4747.465	0	0	0	0	0	0	811.795	0	0	0	0	0	0	19417.757
**Ascariasis**	168.178	36.53	5562.586	5931.73	47235.335	12285.315	144660.743	94.227	15.003	3798.47	4017.951	30412.5	8584.016	107086.442	263.932	68.902	8206.315	8498.006	72403.505	17859.282	190812.296
***Bordetella* spp. (Pertussis)**	29605.16	10730.887	181459.295	641406.514	2235889.98	875238.26	6262444.587	11765.402	7193.309	81490.345	241125.76	585821.645	366285.791	2562200.335	62728.184	15574.778	350495.466	1391144.917	5076930.224	1742531.588	12562122.88
***Campylobacter* spp.**	15338.221	7078.129	79100.261	56638.07	1472481.775	124803.429	2357864.369	5578.37	2604.085	30837.761	16956.351	637131.113	45411.477	897692.959	33988.765	14957.559	162867.688	141042.507	2768889.365	280922.916	4891280.343
**Chagas disease**	0	44.735	118.006	0	0	0	0	0	15.008	63.693	0	0	0	0	0	92.975	301.307	0	0	0	0
***Chlamydia* spp.**	35284.259	6159.92	86869.621	219125.717	1426968.423	249296.69	2223828.177	27168.039	3428.622	58227.95	153635.194	1036617.532	182399.905	1690716.271	46120.43	10117.862	123191.52	301055.935	1910115.521	326697.758	2927350.709
***Citrobacter* spp.**	10461.361	6372.216	40784.871	50528.491	302683.467	107485.05	348601.396	5405.081	3493.499	25693.707	28478.547	176071.583	66361.665	204855.499	18603.967	10671.999	62953.326	81735.207	492440.594	165030.876	556661.762
Clostridioides difficile	11200.325	37107.993	55659.575	10339.463	14086.894	55543.952	26831.438	5801.129	23039.184	27937.301	4286.557	5876.898	30860.175	11905.133	18987.982	57798.249	97374.729	21047	29571.608	94383.029	53187.914

***Cryptosporidium* spp.**	8984.586	1560.443	39558.468	173931.945	676393.685	45646.699	4088131.991	1413.336	232.602	6152.959	29204.451	118876.078	6953.85	920252.946	28655.871	5171.14	130479.784	498404.683	1979296.68	153749.462	9346440.872
**Cutaneous and mucocutaneous leishmaniasis**	13.7	5.111	508.062	3998.424	184.981	3.364	116.644	5.016	2.567	296.068	2407.516	93.584	0.189	55.977	27.697	8.697	881.915	6091.449	335.421	13.16	208.322
**Cystic echinococcosis**	140.385	22.532	32.66	1489.222	2628.234	280.473	12606.765	20.36	0.989	0.25	396.818	558.897	45.758	4092.012	352.834	65.601	100.227	2915.177	5559.387	733.741	23000.167
**Cysticercosis**	0.105	0.041	90.354	0	13.576	8.087	1612.882	0	0	0	0	0	0.142	15.416	0.32	0.127	268.811	0	42.863	20.653	4514.075
**Dengue virus**	4.235	116.298	21541.18	2624.336	182919.673	413145.076	8117.84	1.133	25.375	15005.067	1874.426	35781.202	102868.674	1197.718	13.465	287.27	26623.329	3713.698	293533.918	601597.203	17309.552
**Diphtheria**	137.949	69.524	1118.561	5600.344	15299.144	6174.305	694456.54	68.286	55.227	620.827	2827.396	9542.78	4159.002	427686.54	257.609	86.325	1976.135	10181.662	23100.887	8718.123	1067476.219
**Ebola virus**	0	0	0	0	0	0	27954.516	0	0	0	0	0	0	22918.06	0	0	0	0	0	0	32991.828
Entamoeba histolytica	4591.858	840.21	58456.387	109187.716	414611.08	15958.95	821122.765	1138.455	202.542	16328.015	26698.105	109068.31	3814.146	205609.456	12127.75	2374.671	138486.496	295999.239	1016487.409	43350.034	2114002.167
***Enterobacter* spp.**	48826.884	33473.966	204362.332	278874.519	1751960.349	580832.636	1632176.041	33220.41	20545.639	134918.373	177178.806	1172016.164	387828.961	1160353.088	72642.546	52280.136	296772.427	414859.141	2579933.306	841634.813	2277381.308
Enterococcus faecalis	24624.825	20687.754	104317.941	106810.883	605488.749	148426.605	986856.838	16174.076	12929.733	72904.798	66102.809	382151.671	102437.151	678999.466	37684.418	32237.947	147902.026	165867.044	923749.421	215742.099	1420957.977
Enterococcus faecium	15564.728	18610.303	71569.848	66612.727	269851.845	94380.606	459667.22	9970.724	11261.208	48025.27	39315.292	168986.107	62312.373	303673.859	24435.351	29349.041	105831.964	105910.907	420451.954	141156.273	685529.823
**Enteropathogenic *Escherichia coli***	1513.147	861.815	5282.368	44616.309	180948.249	37214.361	764773.892	691.363	359.483	2264.589	18497.707	78657.104	15763.504	341169.588	2940.147	1796.943	10288.508	88817.267	354153.688	75470.006	1501006.82
**Enterotoxigenic *Escherichia coli***	5639.117	3951.108	7065.984	66006.621	376266.67	46126.903	434091.743	2264.91	1540.541	2667.415	24160.488	146510.317	16021.068	163941.569	12313.994	7955.519	15662.272	157410.248	793150.519	103063.823	988343.56

Escherichia coli	124979.263	75199.97	343970.082	593313.64	3047924.839	686348.122	5685065.142	94852.38	49283.889	238878.674	425716.891	2250675.769	519181.788	4256832.531	165186.672	112250.054	475665.485	809279.448	4061645.83	891926.33	7655459.968
**Food-borne trematodiases**	33.841	48.243	26.974	18.739	0	1546.091	0	16.591	15.615	9.87	5.158	0	883.687	0	60.402	95.514	52.429	37.554	0	2521.554	0
**Fungi**	59210.947	22579.614	367227.215	596831.367	4066173.952	671457.212	8472973.312	33297.995	12431.798	220539.638	312922.43	2147143.342	407184.762	5030740.109	97465.149	38135.083	579636.907	1001377.39	6905732.498	1060936.862	13239256.45
**Genital herpes**	0	0	0	0	0	0	0	0	0	0	0	0	0	0	0	0	0	0	0	0	0
**Group A Streptococcus (*Streptococcus pyogenes*)**	45966.199	44709.303	164979.615	167334.665	650085.723	218452.408	1146483.058	30188.494	28221.943	112736.396	102813.898	427941.201	148049.42	772298.782	69368.744	66274.173	232293.124	253047.461	982041.617	314468.119	1655884.319
**Group B Streptococcus (*Streptococcus agalactiae*)**	75504.723	43325.535	244815.589	400026.515	2213232.882	496857.655	3523971.118	55936.323	27924.58	166041.519	267216.625	1586545.219	361929.44	2666756.751	100235.371	63979.392	339018.311	558292.872	2946004.056	660620.439	4647582.135
**Guinea worm disease**	0	0	0	0	0	0	0	0	0	0	0	0	0	0	0	0	0	0	0	0	0
**HIV/AIDS**	44150.084	8037.395	170981.455	64523.507	259413.605	324526.482	4015615.588	43184.319	7639.133	119234.707	19873.408	141024.878	224768.822	3082448.509	45120.705	8564.343	286932.244	252278.229	779931.82	471191.693	5168552.158
Haemophilus influenzae	44064.17	8836.612	91349.898	184638.327	1025501.515	274933.239	2114644.434	35225.508	6562.317	66687.498	138036.629	749389.445	216576.683	1592260.659	55095.241	11795.295	120571.022	246455.657	1340775.529	342753.592	2767511.816
Helicobacter pylori	0	0	0	0	0	0	0	0	0	0	0	0	0	0	0	0	0	0	0	0	0
**Hepatitis A**	2048.113	702.494	5316	37345.024	623704.044	8293.473	59622.26	1135.52	511.273	3922.035	13743.309	352836.458	5894.503	30686.954	3063.705	959.101	7040.945	72051.762	997230.752	11174.294	115136.667
**Hepatitis B**	5797.885	644.455	3848.459	22818.863	275972.916	16981.733	103025.724	4241.027	474.902	2417.539	8830.964	94731.282	12758.677	65364.1	7827.653	789.602	5237.688	48002.138	502938.751	20898.375	165680.374
**Hepatitis C**	503.29	215.762	898.974	1855.333	43258.751	1952.828	12858.965	332.219	153.543	615.001	1029.779	12770.734	1345.102	5741.03	765.971	301.652	1382.184	3606.267	78504.901	2991.023	23389.173

**Hepatitis E**	411.806	123.683	563.261	1212.883	30191.554	1202.238	4246.821	119.499	61.234	352.965	373.748	14212.645	855.073	1608.123	854.528	158.055	853.455	2711.876	56750.164	1819.995	9187.845
**Hookworm disease**	100.682	54.491	1883.906	1414.029	10950.677	5992.03	51564.424	55.97	24.79	1128.079	855.002	6499.816	3558.569	32816.722	162.553	101.999	2978.198	2174.472	17104.363	9789.615	76300.678
**Human papillomavirus**	0	0	0	0	0	0	0	0	0	0	0	0	0	0	0	0	0	0	0	0	0
**Influenza virus**	155994.689	28592.042	324053.644	590206.822	3244195.671	766311.731	5803439.594	125502.759	21233.673	235449.837	440835.907	2424649.527	600477.689	4409891.47	195370.126	38587.371	426982.581	767356.845	4232843.926	956939.262	7569617.726
**Invasive Non-typhoidal *Salmonella* (iNTS)**	31689.167	20061.072	151220.027	326242.676	1400799.024	422138.621	7886586.523	16172.954	6161.166	82656.072	172229.31	899787.337	245636.703	4653362.917	57318.401	48755.6	252941.846	590853.472	2091738.354	681892.73	12128455.85
Klebsiella pneumoniae	176942.034	78914.498	606221.583	921660.38	4920981.536	1140842.879	9339169.113	134269.663	51093.271	422174.587	647117.737	3566547.006	857979.936	7048667.598	235479.527	116307.777	840300.12	1288780.562	6677506.811	1484739.1	12123349.68
***Legionella* spp.**	22980.54	7043.827	51404.583	103680.77	636716.077	132987.983	754150.829	15580.033	3547.447	29721.218	59130.516	370050.269	82262.334	438582.461	33285.639	12809.514	82803.723	168924.84	1032058.416	206146.444	1220253.655
**Leprosy**	0	0	0	0	0	0	0	0	0	0	0	0	0	0	0	0	0	0	0	0	0
Listeria monocytogenes	2751.916	2353.598	11062.3	21083.873	98866.972	30823.161	377225.317	1608.598	1440.336	6402.441	9665.842	60246.524	19140.351	217866.143	4392.347	3549.307	17192.377	40653.047	154297.533	46211.717	631385.925
**Lymphatic filariasis**	0	0	0	0	0	0	0	0	0	0	0	0	0	0	0	0	0	0	0	0	0
**Malaria**	3.27	3.351	16280.799	121719.563	1073748.349	30173.697	35919459.41	2.32	1.324	5799.069	43816.636	442582.699	9665.126	17105956.99	4.463	7.693	37304.21	265746.837	2286469.652	74167.382	62653808.77
**Measles**	1064.768	211.931	60.541	324879.994	820337.308	582458.475	6214454.538	794.829	151.84	42.495	105511.107	278648.949	218037.452	2183815.219	1377.913	272.682	113.14	730089.728	1862754.54	1228543.205	13685858.63
***Morganella* spp.**	80.882	54.51	425.681	436.436	2265.608	436.424	1339.531	36.378	25.688	230.378	130.526	1093.487	174.021	667.099	162.302	112.48	761.567	987.161	4038.25	939.845	2485.863
***Mycoplasma* spp.**	47648.407	8738.202	92072.294	187842.898	1056576.101	235639.676	1860810.603	38138.843	6084.538	66567.434	134859.875	752936.378	181248.361	1381356.529	59803.633	12332.12	123832.379	252549.22	1412479.893	300029.574	2472167.895
Neisseria	0	0	0	0	0	0	0	0	0	0	0	0	0	0	0	0	0	0	0	0	0

gonorrhoeae																					
Neisseria meningitidis	44410.017	23390.402	235907.026	336101.649	1535865.086	444441.473	3442710.575	28455.697	14527.709	155390.32	207501.81	1027668.917	305591.698	2357826.518	68089.951	34839.857	341291.387	521334.525	2269874.904	628950.817	4961477.406
**Norovirus**	19905.162	16599.335	125448.457	201925.489	563384.798	151391.914	2010091.561	5551.654	4297.308	37682.227	54233.13	160960.002	43376.175	524828.461	41870.461	33710.892	246489.975	451330.762	1212122.311	321262.772	4234973.52
**Onchocerciasis**	0	0	0	0.026	0	0	0	0	0	0	0.012	0	0	0	0	0	0	0.049	0	0	0
**Other *Klebsiella* spp.**	1338.174	1669.692	6460.329	6163.304	13143.816	12930.399	45634.19	527.079	590.87	2519.175	2287.048	4702.165	5203.462	16850.634	2872.86	4004.066	13688.966	13831.915	28722.361	26821.547	93108.054
**Other *Enterococcus* spp.**	12583.328	10840.599	62006.039	64856.593	381398.153	102980.335	506181.82	7342.776	6384.325	40070.052	36717.427	223204.504	65553.747	307426.604	20485.234	17185.257	93248.303	104925.535	611106.497	153324.709	795925.773
**Other neglected tropical diseases**	12730.273	8955.903	41729.428	42980.339	225432.947	44486.65	855322.98	8663.179	5997.363	29885.173	28715.188	156229.777	30601.484	488805.983	17997.666	13075.023	57388.047	62051.217	315227.294	62358.674	2457712.11
**Other unspecified infectious diseases**	46889.678	41406.184	62042.704	92963.472	565718.782	175911.967	831623.865	17108.962	31592.859	45439.614	57540.719	317826.94	112222.048	558769.44	74011.697	54237.573	88846.48	185662.4	851502.025	225000.496	1108990.062
**Polymicrobial infections**	81084.227	61332.91	371570.888	474746.358	3153456.879	822371.74	3240865.47	50287.504	35149.037	236069.303	281768.761	1933041.728	533301.781	2198982.465	124809.987	98012.375	539525.734	719866.7	4719712.67	1199300.294	4697370.838
***Proteus* spp.**	5417.223	4603.755	26406.814	26742.755	135802.927	36800.76	233543.279	3438.795	2852.129	17811.438	15854.131	86353.599	24032.246	153205.262	8241.265	7167.19	37858.949	41394.417	213542.133	53191.8	343297.71
***Providencia* spp.**	82.617	36.985	454.421	779.166	3417.44	580.651	2345.715	30.605	13.908	208.926	190.571	1451.402	201.447	999.301	187.512	87.363	918.641	2013.061	6876.06	1373.348	4566.054
Pseudomonas aeruginosa	117405.142	56971.232	361188.701	496316.336	2617456.322	669393.454	3889717.979	90083.249	36178.882	247587.422	336085.502	1862584.673	485312.128	2920864.057	154853.346	84930.721	502786.17	686461.033	3525836.107	881908.157	5090873.279
**Rabies**	94.62	68.099	78.675	507.569	44033.87	8928.833	122866.053	52.757	57.862	23.452	124.148	21646.809	2042.326	36514.998	181.942	80.112	158.368	1295.967	77922.744	16159.453	234487.196

**Respiratory syncytial virus**	254155.37	63870.539	476037.79	796501.27	4282667.388	1128555.323	6069017.529	203470.711	43138.022	332874.007	571029.69	3196956.431	872631.135	4673132.883	319674.826	94194.129	646511.019	1051380.173	5529566.983	1416853.681	7837596.713
**Rotavirus**	31846.436	21333.429	198259.264	681512.442	866644.211	724428.975	7803778.206	14050.234	8759.411	93037.674	302342.551	377495.067	376763.257	3831001.012	56894.824	40410.102	350689.858	1237000.147	1562651.32	1143362.618	12946328.38
***Salmonella* Paratyphi**	5.49	12.862	64.151	205.808	210801.415	14110.375	9981.112	2.357	7.748	23.921	53.862	65156.269	4016.184	3068.258	11.551	16.643	146.759	533.999	503579.765	34754.029	23932.189
***Salmonella* Typhi**	9661.072	2931.637	72878.14	188357.946	1976068.957	248457.479	3229921.861	5495.944	1657.031	46158.826	114041.69	1144013.298	147615.138	2114917.205	15852.933	4742.91	108001.776	283775.4	3039654.588	395080.734	4729394.117
**Schistosomiasis**	0	0	325.282	960.978	0	455.47	30496.854	0	0	102.003	319.43	0	194.965	19254.337	0	0	782.662	2202.478	0	964.754	48213.176
***Serratia* spp.**	12611.779	9033.702	83729.729	99817.441	661759.891	151497.685	988724.533	7741.402	5603.73	54843.489	59819.23	400439.062	99770.31	627675.463	19891.586	14016.661	120525.97	154066.277	1035998.12	222961.163	1491675.405
***Shigella* spp.**	15075.537	3308.456	103980.607	174305.482	824585.845	152898.764	4315043.809	5089.164	1128.564	42305.759	55980.294	305699.825	56394.729	1842253.623	32242.001	7676.428	198383.574	399671.172	1645555.843	308284.488	7881213.906
Staphylococcus aureus	195571.236	107271.429	547147.355	725464.729	3370076.671	1006876.853	6123264.3	153990.03	69608.59	385080.247	522042.624	2535914.904	769170.386	4691052.873	249071.594	156733.73	751383.694	981714.397	4379175.402	1293867.274	7974576.125
Streptococcus pneumoniae	309626.976	64093.565	627840.645	1177227.013	6580497.707	2007452.966	12240097.15	249189.085	44855.318	460430.704	905280.401	5133050.67	1635300.234	9423350.011	388901.654	91058.924	826487.03	1505652.832	8383326.402	2490424.184	15743139.47
**Syphilis**	14685.723	11977.821	225567.867	284660.025	1557921.963	949866.338	6198967.286	4926.911	4943.796	95935.48	85511.305	482682.835	293003.933	2180404.555	29929.397	23734.363	443685.744	635393.864	3430058.892	2108428.635	12838090.07
**Tetanus**	90.258	85.756	15222.464	72171.837	739482.171	83518.795	954657.535	62.568	67.651	7149.24	35235.128	456443.16	60342.305	654869.583	239.944	128.879	34996.288	129819.42	1097892.878	112392.503	1547844.28
**Trachoma**	0	0	0	0	0	0	0	0	0	0	0	0	0	0	0	0	0	0	0	0	0
**Trichomoniasis**	0	0	0	0	0	0	0	0	0	0	0	0	0	0	0	0	0	0	0	0	0
**Trichuriasis**	0	1.324	1186.865	41.451	3353.455	4298.445	4393.286	0	0.631	642.787	18.29	1793.321	2274.513	2466.999	0	2.43	2014.386	79.88	5934.127	7210.591	7111.785
**Tuberculosis**	42304.014	2409.299	58076.474	73622.386	1325447.598	419229.76	4330445.153	33989.033	2079.497	45104.399	49365.9	1059718.906	342439.228	3270660.684	52397.731	2786.086	73204.698	100720.49	1648530.053	506361.789	5666806.105
**Varicella and**	4205.939	4734.179	26057.214	26369.575	131667.936	41365.134	302196.832	2592.03	3443.184	18733.344	17406.832	99384.373	33252.929	224114.57	5685.842	8585.713	39911.597	39136.61	171346.645	51777.461	400744.254

**herpes zoster**																					
Vibrio cholerae	12783.248	0	38081.955	361667.486	243699.529	102652.362	2657637.209	6423.945	0	16849.851	159189.663	115746.303	52076.964	1187873.149	24261.416	0	76495.582	756213.259	440330.95	187729.582	4822641.049
**Viral meningitis**	4327.56	3710.221	16173.692	35170.707	146050.65	42211.996	592551.246	2819.111	2508.817	10299.168	17384.583	101968.058	29852.784	380088.797	6642.918	5176.603	23590.908	71511.014	210340.446	59583.263	876762.83
**Visceral leishmaniasis**	852.386	211.745	17685.144	10129.854	25918.44	23.642	83918.71	1.509	0.622	24.968	19.676	35.25	2.457	42604.646	8200.013	2019.734	70558.873	93599.727	144372.89	100.085	137743.526
**Yellow fever**	0	232.997	414.438	2249.79	0	0	41128.799	0	18.522	108.798	242.084	0	0	14445.355	0	1064.269	1144.428	9122.165	0	0	90560.807
**Zika virus**	0	2.413	105.055	0	0	0	11.132	0	0.743	62.037	0	0	0	2.444	0	5.929	174.427	0	0	0	30.717
**TOTAL**	**2366522.258**	**1060167.637**	**7702197.872**	**13563138.78**	**71270512.65**	**19143384.13**	**193341320.2**	**1896953.972**	**754325.504**	**5702475.892**	**10396241.69**	**55771055.06**	**15427699.04**	**157849128.8**	**2991580.973**	**1457356.095**	**10139173.69**	**17244786.47**	**90080106.31**	**23319033.95**	**237012515.2**

Section 7: Estimation Flowchart

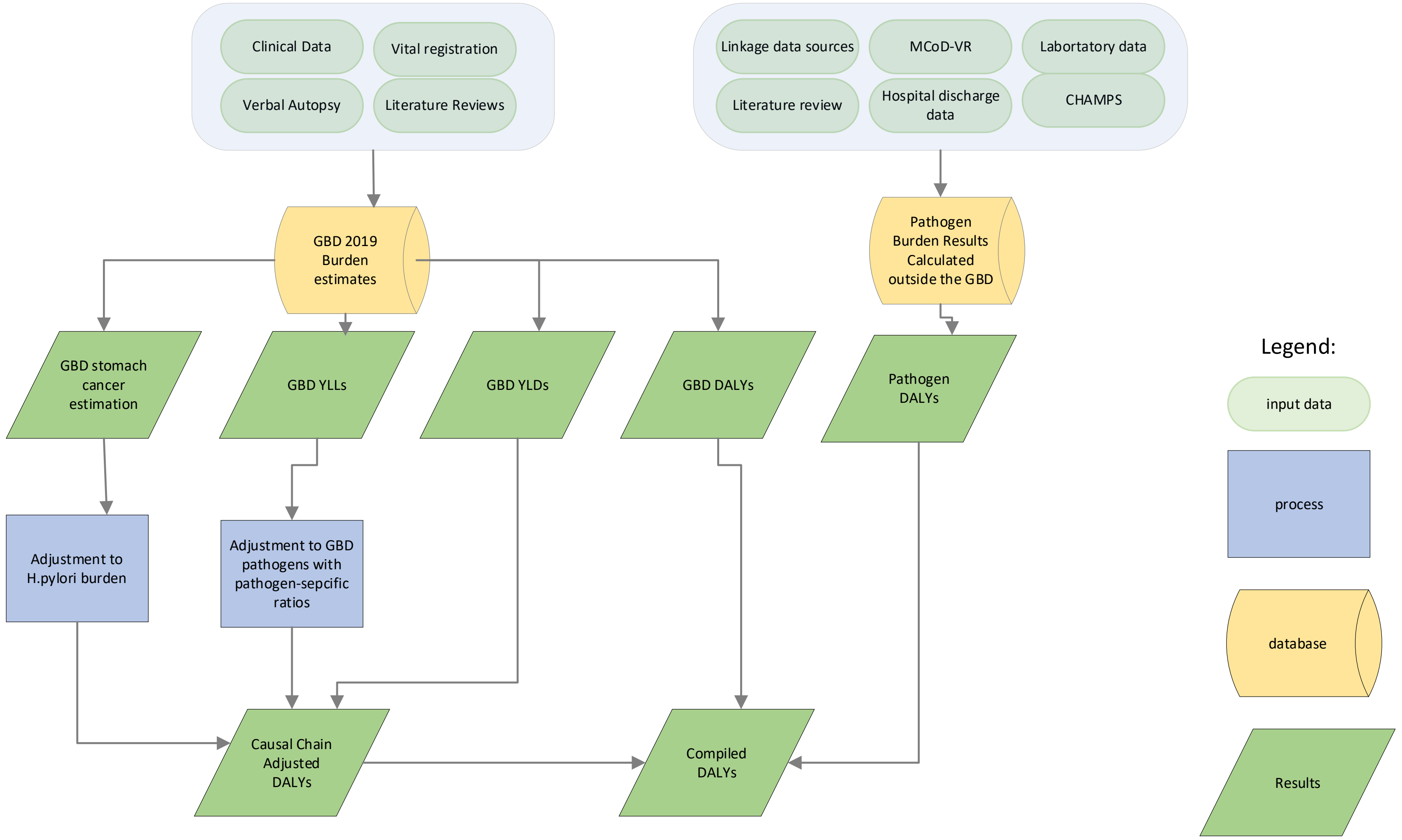

Section 8: GATHER Compliance: Guidelines for Accurate and Transparent Health Estimates Reporting

This study complies with GATHER recommendations. We have documented the steps in our analytical procedures and detailed the data sources used. The GATHER recommendations can be found on the GATHER website.

Checklist of information that should be included in new reports of global health estimates

Item #	Checklist item	Reported location
Objectives and funding		
1	Define the indicator(s), populations (including age, sex, and geographic entities), and time period(s) for which estimates were made.	This can be found in the methods section of the main text.
2	List the funding sources for the work.	This can be found in the Funding Section of the main text.
Data Inputs		
For all data inputs from multiple sources that are synthesized as part of the study:		
3	Describe how the data were identified and how the data were accessed.	This can be found in the Overview and Sources of Information and in the data sources in the appendix.
4	Specify the inclusion and exclusion criteria. Identify all ad-hoc exclusions.	This is relevant to some literature studies and can be found in the appendix.
5	Provide information on all included data sources and their main characteristics. For each data source used, report reference information or contact name/institution, population represented, data collection method, year(s) of data collection, sex and age range, diagnostic criteria or measurement method, and sample size, as relevant.	The full list of data sources is available here . The estimates from the GBD study can be found in the GBD results tool and the AMR estimates can be found here .
6	Identify and describe any categories of input data that have potentially important biases (e.g., based on characteristics listed in item 5).	Data limitations can be found in the Limitations section of the main text.
For data inputs that contribute to the analysis but were not synthesized as part of the study:		
7	Describe and give sources for any other data inputs.	The published estimates can be found in the GHDx.
For all data inputs:		
8	Provide all data inputs in a file format from which data can be efficiently extracted (e.g., a spreadsheet rather than a PDF), including all relevant meta-data listed in item 5. For any data inputs that cannot be shared because of ethical or legal reasons, such as third-party ownership, provide a contact name or the name of the institution that retains the right to the data.	The results of this study and the input data citations can be found in the GHDx.
Data analysis		
9	Provide a conceptual overview of the data analysis method. A diagram may be helpful.	This can be found in the methods section.
10	Provide a detailed description of all steps of the analysis, including mathematical formulae. This description should cover, as relevant, data cleaning, data pre-processing, data adjustments and weighting of data sources, and mathematical or statistical model(s).	This can be found in the methods section.
11	Describe how candidate models were evaluated and how the final model(s) were selected.	This can be found in the methods appendix in the section for pathogen distribution. The results pulled from the GBD have been extensively published previously.

12	Provide the results of an evaluation of model performance, if done, as well as the results of any relevant sensitivity analysis.	This can be found in the methods appendix for pathogen distribution, infectious syndrome, case fatality rate.
13	Describe methods for calculating uncertainty of the estimates. State which sources of uncertainty were, and were not, accounted for in the uncertainty analysis.	This can be found in the methods appendix in each modelling component section.
14	State how analytic or statistical source code used to generate estimates can be accessed.	Published code is available on github
Results and Discussion		
15	Provide published estimates in a file format from which data can be efficiently extracted.	The published estimates can be found in the GHDx.
16	Report a quantitative measure of the uncertainty of the estimates (e.g. uncertainty intervals).	All estimates reported have uncertainty intervals.
17	Interpret results in light of existing evidence. If updating a previous set of estimates, describe the reasons for changes in estimates.	The research in context section explains these results in light of existing evidence.
18	Discuss limitations of the estimates. Include a discussion of any modelling assumptions or data limitations that affect interpretation of the estimates.	The limitations section of the main text describes data and modeling limitations.

Section 9: References

1 Antimicrobial Resistance Collaborators. Global burden of bacterial antimicrobial resistance in 2019: a systematic analysis. *The Lancet* 2022; **399**: 629–55.

2 Antimicrobial Resistance Collaborators. The burden of antimicrobial resistance in the Americas in 2019: a cross-country systematic analysis. *Lancet Reg Health - Am* 2023; **25**: 100561.

3 GBD 2019 Diseases and Injuries Collaborators. Global burden of 369 diseases and injuries in 204 countries and territories, 1990–2019: a systematic analysis for the Global Burden of Disease Study 2019. *The Lancet* 2020; **396**: 1204–22.

4 GBD 2019 Demographics Collaborators. Global age-sex-specific fertility, mortality, healthy life expectancy (HALE), and population estimates in 204 countries and territories, 1950–2019: a comprehensive demographic analysis for the Global Burden of Disease Study 2019. *The Lancet* 2020; **396**: 1160–203.

5 Rudd KE, Johnson SC, Agesa KM, *et al.* Global, regional, and national sepsis incidence and mortality, 1990–2017: analysis for the Global Burden of Disease Study. *The Lancet* 2020; **395**: 200–11.

6 Mandrekar JN. Receiver operating characteristic curve in diagnostic test assessment. *J Thorac Oncol Off Publ Int Assoc Study Lung Cancer* 2010; **5**: 1315–6.

7 Zheng P, Barber R, Sorensen RJD, Murray CJL, Aravkin AY. Trimmed constrained mixed effects models: formulations and algorithms. *J Comput Graph Stat* 2021; **30**: 544–56.

8 Ewig S, Schlochtermeier M, Göke N, Niederman MS. Applying sputum as a diagnostic tool in pneumonia: limited yield, minimal impact on treatment decisions. *Chest* 2002; **121**: 1486–92.

9 Ogawa H, Kitsios GD, Iwata M, Terasawa T. Sputum gram stain for bacterial pathogen diagnosis in community-acquired pneumonia: a systematic review and bayesian meta-analysis of diagnostic accuracy and yield. *Clin Infect Dis Off Publ Infect Dis Soc Am* 2020; **71**: 499–513.

10 Feikin DR, Scott JAG, Gessner BD. Use of vaccines as probes to define disease burden. *Lancet Lond Engl* 2014; **383**: 1762–70.

11 GBD 2016 Lower Respiratory Infections Collaborators. Estimates of the global, regional, and national morbidity, mortality, and aetiologies of lower respiratory infections in 195 countries, 1990-2016: a systematic analysis for the Global Burden of Disease Study 2016. *Lancet Infect Dis* 2018; **18**: 1191–210.

12 Johnson HL, Deloria-Knoll M, Levine OS, *et al.* Systematic evaluation of serotypes causing invasive pneumococcal disease among children under five: The Pneumococcal Global Serotype Project. *PLOS Med* 2010; **7**: e1000348.

13 Grijalva CG, Nuorti JP, Arbogast PG, Martin SW, Edwards KM, Griffin MR. Decline in pneumonia admissions after routine childhood immunisation with pneumococcal conjugate vaccine in the USA: a time-series analysis. *Lancet Lond Engl* 2007; **369**: 1179–86.

14 European Antimicrobial Resistance Collaborators. The burden of bacterial antimicrobial resistance in the WHO European region in 2019: a cross-country systematic analysis. *Lancet Public Health* 2022; **7**: e897–913.

15 Nocedal J, Wright S. Numerical Optimization, 2nd edn. New York: Springer-Verlag, 2006 https://link.springer.com/book/10.1007/978-0-387-40065-5 (accessed Aug 8, 2022).

16 Martel C de, Georges D, Bray F, Ferlay J, Clifford GM. Global burden of cancer attributable to infections in 2018: a worldwide incidence analysis. *Lancet Glob Health* 2020; **8**: e180–90.

Section 10: Authors' Contributions

Managing the overall research enterprise

Ben S Cooper, Susanna Dunachie, Simon I Hay, Christopher J L Murray, Mohsen Naghavi, Amanda Novotney, and Eve E Wool.

Writing the first draft of the manuscript

Tomislav Mestrovic, Christopher J L Murray, and Mohsen Naghavi.

Primary responsibility for applying analytical methods to produce estimates

Rose Bender, Annie J Browne, Erin Chung, Christiane Dolecek, Matthew Doxey, Anna Gershberg Hayoon, Authia P Gray, Georgina Haines-Woodhouse, Chieh Han, Kevin S Ikuta, Jorge R Ledesma, Jianing Ma, Vincent Mougin, Gisela Robles Aguilar, Christopher Troeger, Magdalene K Walters, and Han Yong Wunrow.

Primary responsibility for seeking, cataloguing, extracting, or cleaning data; designing or coding figures and tables

Samuel B. Albertson, Daniel Araki, Matthew W Cunningham, Christiane Dolecek, Matthew Doxey, Susanna Dunachie, Anna Gershberg Hayoon, Sama Ghoba, Authia P Gray, Georgina Haines-Woodhouse, Chieh Han, Rebecca Hsu, Kevin S Ikuta, Jorge R Ledesma, Jianing Ma, Catrin E Moore, Vincent Mougin, Mohsen Naghavi, Gisela Robles Aguilar, and Avina Vongpradith.

Providing data or critical feedback on data sources

Erin Chung, Christiane Dolecek, Susanna Dunachie, Georgina Haines-Woodhouse, Chieh Han, Simon I Hay, Kevin S Ikuta, Kenneth C Iregbu, Hmwe H Kyu, Tomislav Mestrovic, Catrin E Moore, Christopher J L Murray, Mohsen Naghavi, Gisela Robles Aguilar, Victor Daniel Rosenthal, Benn Sartorius, Andy Stergachis, and Christopher Troeger.

Developing methods or computational machinery

Greg Bertolacci, Authia P Gray, Georgina Haines-Woodhouse, Chieh Han, Simon I Hay, Rebecca Hsu, Kevin S Ikuta, Christopher J L Murray, Mohsen Naghavi, Pirouz Naghavi, Gisela Robles Aguilar, Lucien R Swetschinski, Christopher Troeger, and Han Yong Wunrow.

Providing critical feedback on methods or results

Annie J Browne, Ben S Cooper, Christiane Dolecek, Susanna Dunachie, Georgina Haines-Woodhouse, Simon I Hay, Kevin S Ikuta, Kenneth C Iregbu, Hmwe H Kyu, Tomislav Mestrovic, Catrin E Moore, Jonathan Mosser, Christopher J L Murray, Mohsen Naghavi, Gisela Robles Aguilar, Victor Daniel Rosenthal, Benn Sartorius, Andy Stergachis, Lucien R Swetschinski, and Christopher Troeger.

Drafting the work or revising is critically for important intellectual content

Erin Chung, Nicole Davis Weaver, Christiane Dolecek, Susanna Dunachie, Georgina Haines-Woodhouse, Simon I Hay, Kevin S Ikuta, Kenneth C Iregbu, Hmwe H Kyu, Tomislav Mestrovic, Jonathan Mosser, Catrin E Moore, Christopher J L Murray, Mohsen Naghavi, Gisela Robles Aguilar, Victor Daniel Rosenthal, Lucien R Swetschinski, and Eve E Wool.

Managing the estimation or publications process

Nicole Davis Weaver, Christiane Dolecek, Simon I Hay, Christopher J L Murray, Mohsen Naghavi, Lucien R Swetschinski, and Eve E Wool.
